# Circumventing Mukaiyama oxidation: selective S–O bond formation via sulfenamide–alcohol coupling

**DOI:** 10.3762/bjoc.22.9

**Published:** 2026-01-20

**Authors:** Guoling Huang, Huarui Zhu, Shuting Zhou, Wanlin Zheng, Fangpeng Liang, Zhibo Zhao, Yifei Chen, Xunbo Lu

**Affiliations:** 1 School of Chemistry and Chemical Engineering, Lingnan Normal University, Zhanjiang, 524048, China; 2 Department of Chemistry, Southern University of Science and Technology, Shenzhen 518055, China

**Keywords:** asymmetric synthesis, late-stage functionalization, selective oxidation, sulfenamides, sulfinimidate esters

## Abstract

A selective and efficient method for the synthesis of sulfinimidate esters via an NBS-promoted oxidative coupling of sulfenamides with alcohols has been developed. This operationally simple, metal-free protocol uses inexpensive and readily available reagents, operates under mild conditions, exhibits a broad substrate scope and high chemoselectivity, and clearly distinguishes itself from classical Mukaiyama-type oxidations. The reaction is readily scalable to gram quantities and is applicable to late-stage functionalization of complex alcohols, including bioactive molecules such as RU58841. Moreover, chiral alcohols such as ʟ-menthol are well tolerated, affording diastereomeric sulfinimidate esters that can undergo stereospecific Grignard substitutions to furnish enantioenriched sulfilimines with up to 93% ee. These results demonstrate the potential of sulfinimidate esters as versatile intermediates for enantioselective S–C bond formation under mild and metal-free conditions.

## Introduction

**Figure 1:**
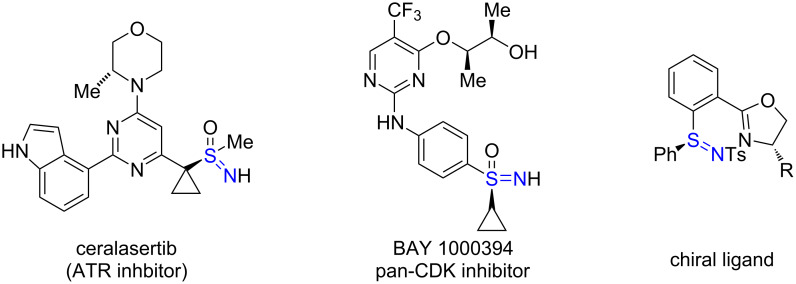
Representative molecules containing a sulfilimine moiety.

Sulfur is a privileged heteroatom in organic chemistry, celebrated for its multiple oxidation states and ability to form diverse bonds with carbon, nitrogen, and oxygen. This versatility underpins the pivotal roles of organosulfur compounds in pharmaceuticals, catalysis, and materials science [1–4]. Among these, sulfilimines (R_2_S=NR') have attracted growing interest due to their unique reactivity and value as intermediates in molecular design and medicinal chemistry [5–10] (Figure 1). In contrast, sulfinimidate esters, which feature a tetravalent sulfur–oxygen (S(=N)–O) motif, remain a relatively underexplored subclass of organosulfur compounds [11–14]. The highly polarized S–O bond imparts distinctive reactivity, making them promising modular electrophilic intermediates for the construction of complex and functionally rich sulfur–nitrogen architectures [14].

Despite their synthetic potential, general and efficient methods for the preparation of sulfinimidate esters remain scarce. For instance, Degennaro and Luisi reported a direct amination strategy using *N*,*N*’-disubstituted sulfenamides in alcoholic solvents; however, this method suffered from limited substrate scope [13]. Subsequently, Malacria and co-workers reported an oxidative transformation of sulfinamides with hypervalent iodine reagents to afford hexavalent sulfonimidates using volatile alcohols as both solvent and nucleophile. While distinct in oxidation state and substrate class, this study offers a valuable precedent for constructing sulfinimidate esters [15–17]. More recently, Wan et al. introduced a TCCA-mediated modular approach to effectively synthesize structurally diverse sulfinimidate esters from readily available sulfenamides and alcohols, significantly expanding this chemical space [18].

**Scheme 1:**
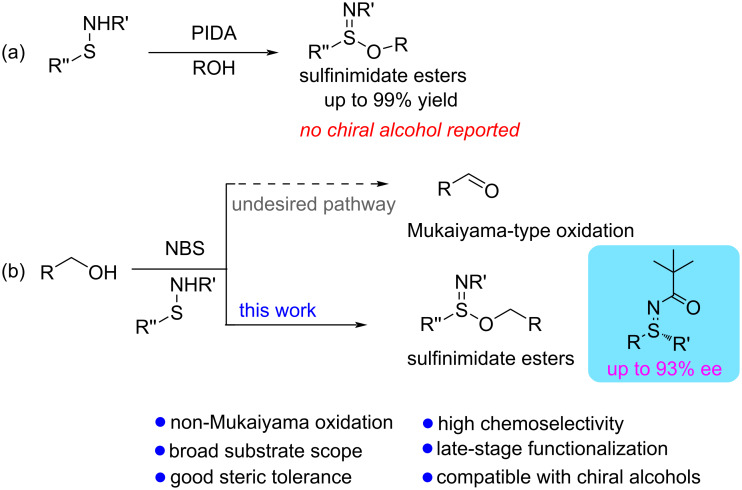
PIDA-mediated approach versus the present NBS-mediated approach to sulfinimidate esters.

Since 2022, sulfenamides have emerged as valuable intermediates for constructing S–C and S–N bonds, particularly in the synthesis of sulfilimines [19–31] and sulfinamidines [32–34]. Their tunable reactivity and modularity have positioned them as versatile scaffolds for sulfur–nitrogen architecture development. Our group previously disclosed a PIDA-mediated oxidative strategy for alcohol incorporation via activation of the S–NH bond (Scheme 1a) [14]. More recently, Wu and co-workers advanced the field by developing a dynamic kinetic resolution protocol for the enantioselective synthesis of sulfinimidate esters from racemic sulfenamide precursors [35]. These studies collectively underscore the synthetic potential of sulfenamides as central electrophilic platforms [36] and the advances regarding the synthesis and reactivity have been comprehensively reviewed very recently [37]. Nevertheless, existing protocols, including our own PIDA-mediated oxidative esterification of sulfenamides, rely on stoichiometric hypervalent iodine reagents and have not demonstrated broadly efficient reactivity with sterically demanding or chiral alcohols such as ʟ-menthol, where only modest conversion was observed in our hands. Motivated by these limitations, we envisioned that highly electrophilic sulfilimidoyl intermediates – generated in situ via *N*-halosuccinimide-mediated oxidative activation of sulfenamides – could be directly intercepted by alcohols under basic conditions to furnish structurally diverse sulfinimidate esters [32]. In particular, we anticipated that an NBS/NaHCO_3_-based protocol would provide a more practical and chiral-alcohol-compatible alternative to our previous PIDA system. A conceptual comparison between our previous PIDA-mediated protocol and the present NBS/NaHCO_3_-mediated protocol is summarized in Scheme 1.

However, such transformations remain synthetically challenging. This is primarily due to the well-known Mukaiyama-type oxidation, wherein sulfenamides serve as redox catalysts for NBS- or NCS-mediated oxidations of alcohols to aldehydes or ketones (Scheme 1b, top) [38–42]. Under such oxidative conditions, the selective S–O bond formation has been largely overlooked, rendering the direct synthesis of sulfinimidate esters from alcohols a nontrivial and underdeveloped transformation.

## Results and Discussion

To validate our hypothesis and identify optimal conditions for sulfilimidate ester formation, we selected the reaction between model sulfenamide **1a** and methanol (**2a**) as the benchmark system (Table 1). Initial attempts in the absence of base led to only trace product formation (Table 1, entry 1), highlighting the essential role of a base in facilitating the transformation. Among several bases screened, NaHCO_3_ emerged as the most effective (Table 1, entry 2), affording the desired product **3a** in 99% isolated yield. Na_2_CO_3_, K_2_CO_3_, and NaOH also gave excellent results, providing **3a** in 92%, 94%, and 93% yield, respectively (Table 1, entries 3–5). In contrast, the stronger base KOH furnished a slightly lower yield (88%, Table 1, entry 6). The organic base Et_3_N afforded **3a** in 89% yield (Table 1, entry 7). These data indicate that mild inorganic bases are generally more appropriate for this transformation, with NaHCO_3_ offering the best combination of efficiency and practicality. Moreover, decreasing the loading of NaHCO_3_ to 1.2 equiv resulted in a noticeably lower yield of **3a** (83%, Table 1, entry 8), underscoring the importance of using a sufficient amount of base. We next examined the effect of different oxidants. While NBS was optimal, yielding **3a** in 99%, the use of NCS or TCCA led to diminished yields (68% and 88%, respectively; Table 1, entries 9 and 10). We tentatively attribute the inferior performance of NCS to its lower halogen-transfer efficiency and oxidation potential under the present conditions, which likely result in a less efficient generation of the key electrophilic sulfur species and in increased competitive decomposition. In contrast, the more strongly oxidizing TCCA tends to promote non-productive overoxidation pathways, thus also failing to match the efficiency of NBS. These results underscore the critical role of halogen source and oxidative strength in modulating the efficiency of the S(=N)–O bond formation. Under the standard conditions (Table 1, entries 1–10), **1a** (0.15 mmol) was treated in MeOH (**2a**, 1.0 mL, ca. 24.6 mmol; ca. 160 equiv relative to **1a**) at room temperature. To improve the compatibility of the protocol with solid or high molecular weight alcohols, we explored the use of alternative solvents while reducing the amount of methanol to 10 or 20 equivalents. Among the solvents tested, including THF, toluene, MeCN, and DCM (Table 1, entries 11–15), DCM performed best, affording the product in up to 65% yield (Table 1, entry 14). In contrast, significantly lower yields were observed in THF and toluene (Table 1, entries 11 and 12), likely due to poor miscibility or reactivity under the reaction conditions. These findings provide a useful basis for further extending the method to structurally more complex or less soluble alcohol substrates.

**Table 1:** Reaction optimization for the synthesis of sulfinimidate esters.^a^

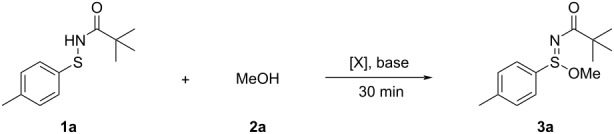				

Entry	Base	Oxidant [X]	Solvent	Yield (%)^b^

1^c^	–	NBS	MeOH	10
2	NaHCO_3_	NBS	MeOH	99
3	Na_2_CO_3_	NBS	MeOH	92
4	K_2_CO_3_	NBS	MeOH	94
5	NaOH	NBS	MeOH	93
6	KOH	NBS	MeOH	88
7	NEt_3_	NBS	MeOH	89
8^d^	NaHCO_3_	NBS	MeOH	83
9	NaHCO_3_	NCS	MeOH	68
10^e^	NaHCO_3_	TCCA	MeOH	88
11^f^	NaHCO_3_	NBS	THF	48
12^g^	NaHCO_3_	NBS	toluene	31
13^h^	NaHCO_3_	NBS	MeCN	60
14^i^	NaHCO_3_	NBS	DCM	65
15^j^	NaHCO_3_	NBS	DCM	63

^a^Unless otherwise specified, all reactions were performed using 0.15 mmol of **1a**, 1.2 equiv of oxidant, and 1.5 equiv of base in 1 mL of methanol at room temperature for 30 minutes; ^b^isolated yields; ^c^reaction performed without base; ^d^1.2 equiv of NaHCO_3_ was used as the base; ^e^0.5 equiv of TCCA was used as the oxidant; ^f–j^ Reactions conducted in 1 mL of the indicated solvent (THF for ^f^, toluene for ^g^, MeCN for ^h^, DCM for ^I,j^), with the amount of MeOH (**2a**) reduced to 20 equivalents^f–i^ or 10 equivalents^j^.

**Scheme 2:**
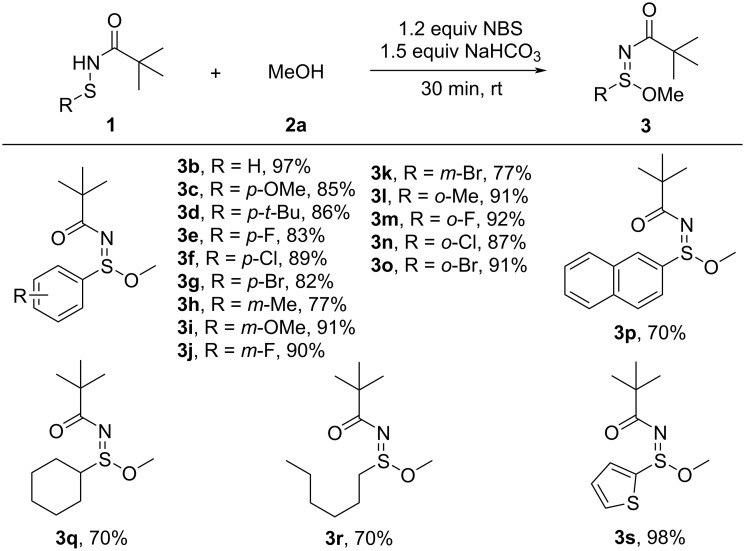
Substrate scope of sulfenamides derived from various thiophenols and thiols. Reaction conditions: sulfenamide **1** (0.15 mmol), NBS (1.2 equiv), NaHCO_3_ (1.5 equiv), MeOH (1.5 mL), room temperature, 30 min. Yields are of isolated products.

With the optimized conditions established, we investigated the substrate scope of sulfenamides derived from various thiophenols and thiols (Scheme 2). A wide range of phenyl sulfenamides bearing substituents at the *para*-, *meta*-, and *ortho*-positions of the aromatic ring, as well as the unsubstituted parent phenyl derivative, were compatible with the reaction (**3b**–**o**). Substrates with electron-donating groups such as methoxy (**3c**, **3i**) and methyl (**3h**, **3l**) as well as electron-withdrawing groups such as fluoro (**3e**, **3j**, **3m**), chloro (**3f**, **3n**), and bromo (**3g**, **3k**, **3o**) all afforded the desired sulfinimidate esters in good to excellent yields (77–92%). Notably, *ortho*-substituted sulfenamides gave consistently high yields (**3l**–**o**, 87–92%), indicating excellent tolerance toward steric hindrance. These results suggest that the reaction is largely insensitive to both steric and electronic effects, underscoring its broad functional group compatibility. The reaction was further applicable to naphthyl sulfenamides, as demonstrated by the successful conversion of a 2-naphthyl derivative to **3p** in 70% yield. In addition, alkyl sulfenamides derived from thiols, including cyclohexyl (**3q**) and linear alkyl chains (**3r**), gave the desired products in 70% yield. Finally, the transformation also accommodated heteroaryl sulfenamides, with a 2-thienyl substrate (**3s**) affording the product in excellent yield (98%), highlighting the broad substrate compatibility of this protocol.

**Scheme 3:**
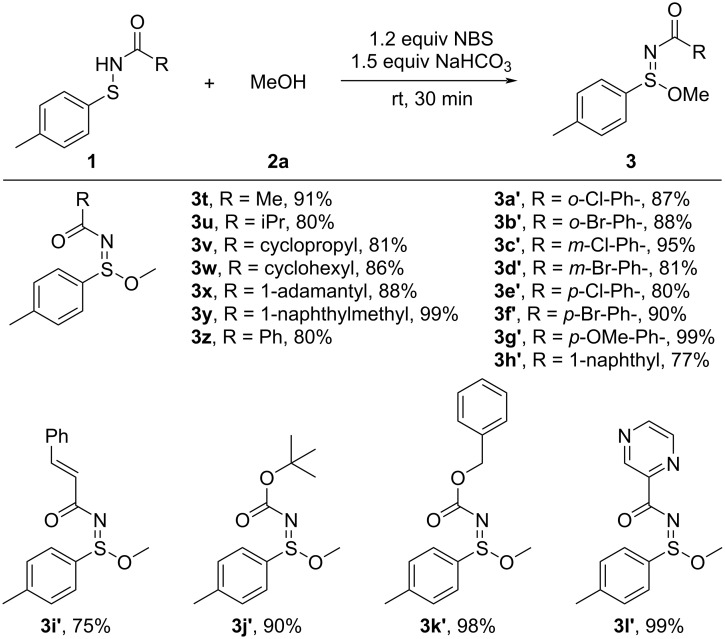
Substrate scope of sulfenamides derived from various amides. Reaction conditions: sulfenamide **1** (0.15 mmol), NBS (1.2 equiv), NaHCO_3_ (1.5 equiv), MeOH (1.5 mL), room temperature, 30 min. Yields are of isolated products.

We then examined the substrate scope of sulfenamides derived from various acylamides to further evaluate the versatility of this transformation (Scheme 3). The reaction was compatible with a wide range of acyl substituents, including methyl (**3t**, 91%), isopropyl (**3u**, 80%), cyclopropyl (**3v**, 81%), and cyclohexyl (**3w**, 86%), all of which delivered the corresponding sulfinimidate esters in good yields. Encouragingly, the method also tolerated bulky acyl groups such as adamantyl (**3x**, 88%) and 1-naphthylmethyl (**3y**, 99%), which underwent smooth conversion without significant steric hindrance. A series of benzamide-derived sulfenamides bearing various substituents on the aromatic ring were also evaluated. The unsubstituted benzamide-derived sulfenamide **3z** reacted smoothly to afford the desired product in 90% yield, serving as a representative standard for comparison. *Ortho*-substituted substrates were compatible, including 2-chloro (**3a’**) and 2-bromo (**3b’**) derivatives which were obtained with 87% and 88% yield, respectively, indicating that steric hindrance at the *ortho*-position does not adversely affect the transformation. *Meta*- and *para*-substituted derivatives, regardless of their electronic nature, also reacted smoothly to afford the corresponding products **3c’**–**g’** in good to excellent yields (up to 99%), suggesting broad functional group compatibility. The protocol also demonstrated excellent compatibility with structurally and electronically distinct amide substrates. The sterically hindered 2-naphthylamide-derived sulfenamide afforded the desired product **3h’** in 77% yield. The cinnamamide-derived sulfenamide, bearing a C–C double bond, also reacted smoothly under the oxidative conditions without noticeable side reactions, delivering the product **3i’** in 75% yield. Furthermore, carbamate-type sulfenamides were well tolerated under the standard conditions, affording the corresponding sulfinimidate esters **3j’** and **3k’** in excellent yields. Notably, a pyrazine-2-carboxamide-derived sulfenamide also underwent efficient transformation to **3l’**, further highlighting the method’s functional group compatibility and applicability to heterocyclic systems.

**Scheme 4:**
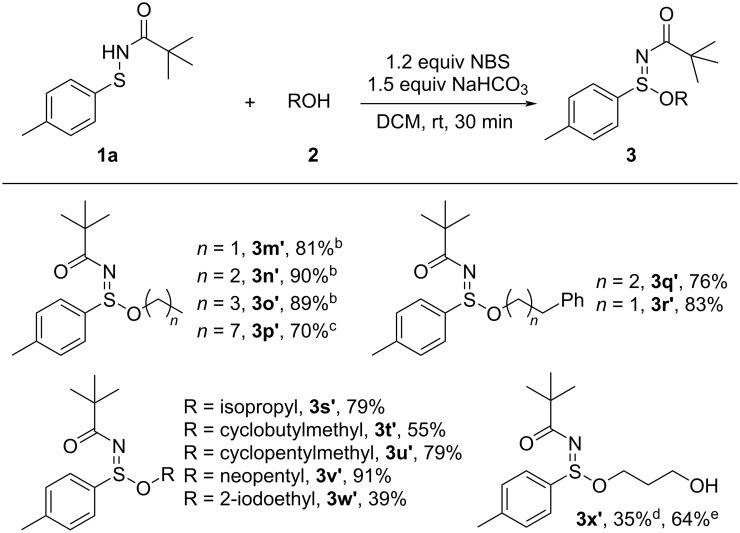
Substrate scope of reactions between sulfenamides **1a** and various alcohols. Reaction conditions: ^a^sulfenamide **1** (0.15 mmol), alcohol **2** (10 equiv), NBS (1.2 equiv), NaHCO_3_ (1.5 equiv), DCM (1.0 mL), room temperature, 30 min. Yields are of isolated products; ^b^alcohol (1.5 mL) was used as the reaction solvent; ^c^2.0 equiv of alcohol were used; ^d^the reaction was conducted on a 0.10 mmol scale of sulfenamide, with 2.5 equiv each of alcohol, NBS, and NaHCO_3_; ^e^the reaction was conducted on a 0.10 mmol scale of sulfenamide, using 2.0 equiv of alcohol, 1.2 equiv of NBS, and 1.5 equiv of NaHCO_3_.

We further investigated the scope of alcohols in the NBS-promoted coupling with sulfenamides under the optimized conditions (Scheme 4). A series of linear alcohols, including simple aliphatic alcohols such as ethanol, 1-propanol, 1-butanol, and 1-octanol, as well as aromatic-substituted alcohols like 3-phenyl-1-propanol and 2-phenylethanol, were well tolerated. These substrates afforded the corresponding sulfinimidate esters **3m’**–**q’** in 70–90% yields. In addition to linear alcohols, cycloalkyl-substituted primary alcohols such as cyclobutylmethanol and cyclopentylmethanol were also examined. These small-ring systems reacted efficiently, providing the corresponding products **3s’** and **3t’** in 79% and 55% yields, respectively. We also evaluated branched and secondary alcohols. Isopropanol as a prototypical secondary branched alcohol, gave an excellent 79% yield of **3u’**, indicating that the transformation proceeds efficiently even with secondary hydroxy groups. The method also accommodated sterically hindered and functionalized alcohols. *tert*-Butylmethanol in which the hydroxy group is attached to a methylene adjacent to a bulky *tert*-butyl group, gave the desired product **3v’** in 91% yield, while a halogenated alcohol bearing an iodoalkyl side chain provided the product **3w’** in 39% yield. Also a hydroxy-functionalized alcohol, derived from prop-1,3-diol, afforded the corresponding sulfinimidate ester **3x’** in 35% yield under the standard conditions (conditions d), where 2.5 equivalents of the alcohol, NBS and NaHCO_3_ were employed in an attempt to access the bis-substituted product. However, these more forcing conditions led to complex mixtures and only modest isolated yield of the monosubstituted product. In contrast, when the equivalents of the alcohol, NBS, and NaHCO_3_ were reduced (conditions e), the reaction proceeded with improved chemoselectivity toward monosubstitution and the yield of product **3x’** increased to 64%. Notably, although ester **3x’** contains two hydroxy groups, no bis-substituted product could be detected under either set of conditions.

**Scheme 5:**
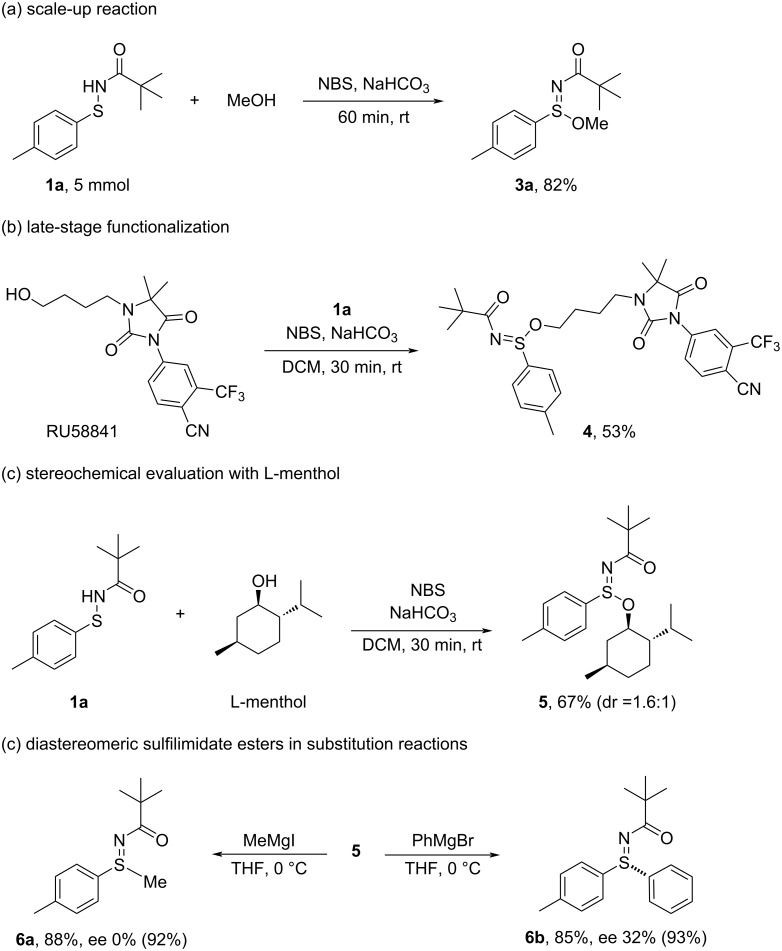
Scale-up synthesis, late-stage derivatization, and substitution of diastereomeric sulfinimidate esters.

To demonstrate the practicality and synthetic utility of this methodology, we carried out a scale-up reaction and applied the protocol to the late-stage modification of a complex alcohol (Scheme 5). The transformation between sulfenamide **1a** and methanol was successfully scaled up to a 3 mmol scale, affording the desired sulfilimidate ester **3a** in an excellent isolated yield of 82%. We further evaluated the protocol’s potential for complex molecule derivatization by modifying RU58841, a non-steroidal antiandrogen under investigation for the treatment of androgenic alopecia and acne [43–44]. Despite the molecule’s dense functionality and sensitive structural motifs, the sulfenamide-based oxidative coupling proceeded smoothly, delivering sulfilimidate ester **4** in 53% yield. In addition, the transformation was applied to a chiral secondary alcohol, ʟ-menthol, to generate the sulfilimidate ester **5** in 67% yield. A diastereomeric analysis revealed a modest dr of 1.6:1, indicating partial stereochemical transfer from the chiral alcohol. We next investigated the stereochemical outcome of the Grignard substitution using diastereomeric sulfilimidate ester **5** (dr = 1.6:1), derived from ʟ-menthol. When treated with MeMgI, product **6a** was obtained in 88% yield but with no enantioenrichment (0% ee), indicating that both diastereomers of **5** reacted at comparable rates to give enantiomeric products in equal amounts, thereby cancelling any net optical activity. In contrast, the reaction of **5** with the bulkier PhMgBr furnished sulfilimine **6b** in 85% yield with 32% ee. This observed ee exceeds the theoretical maximum of 23% expected from the starting diastereomeric ratio, suggesting that the two diastereomers of **5** did not react at identical rates. These results are consistent with a partial kinetic resolution, wherein the major diastereomer of **5** reacts preferentially, leading to the enrichment of one enantiomer in the product mixture.

To further probe this possibility, the two diastereomers of **5** were carefully separated and individually subjected to Grignard substitution. Reactions of the isolated diastereomers with MeMgI and PhMgBr afforded **6a-1** and **6b-1** with 92% ee and 93% ee, respectively (see the Supporting Information File 1 for more details). These results demonstrate that each diastereomer undergoes substitution with high stereospecificity. The moderate ee observed in the reaction of the diastereomeric mixture can therefore be rationalized by the differing intrinsic reactivities of the two diastereomers. Although these findings strongly support the occurrence of a partial kinetic resolution, further kinetic measurements would be required for definitive confirmation.

A plausible mechanism is outlined as follows. First, the sulfenamide **1** is oxidatively brominated by *N*-bromosuccinimide to generate a highly electrophilic sulfilimidoyl bromide intermediate R–S(Br)=N–C(O)R’, along with succinimide. The resulting S(IV)–Br species is then attacked by the alcohol **2**, and nucleophilic substitution at sulfur followed by proton transfer furnishes the sulfinimidate ester **3** and HBr. The latter is neutralized by NaHCO_3_, which acts as an acid scavenger and helps to maintain a mildly basic medium required for the transformation to proceed efficiently.

## Conclusion

In summary, we have developed a mild, metal-free, and operationally simple NBS/NaHCO_3_-promoted oxidative coupling of sulfenamides with alcohols to access sulfinimidate esters. The method uses inexpensive and readily available reagents and features a broad substrate scope, high chemoselectivity, and excellent tolerance toward sterically hindered and functionalized alcohols. It avoids typical Mukaiyama-type oxidation pathways and is applicable to gram-scale synthesis as well as late-stage functionalization of complex molecules such as RU58841. Preliminary studies with ʟ-menthol-derived sulfinimidate esters revealed partial kinetic resolution, and the individually isolated diastereomers underwent stereospecific Grignard substitution to give enantioenriched sulfilimines with up to 93% ee, underscoring the potential of this platform for the streamlined construction of chiral sulfur(IV)–nitrogen frameworks. Further optimization to enhance stereocontrol is currently underway.

## Supporting Information

File 1 Experimental procedures, characterization data and copies of spectra.

## Data Availability

All data that supports the findings of this study is available in the published article and/or the supporting information of this article.
