# Supplementary material for: Circumventing Mukaiyama oxidation: selective S–O bond formation via sulfenamide–alcohol coupling
Source: Beilstein J Org Chem. 2026 Jan 20;22:158–66. doi: 10.3762/bjoc.22.9 (PMC12834055; doi:10.3762/bjoc.22.9)

BEILSTEIN JOURNAL OF ORGANIC CHEMISTRY

Supporting Information

for

Circumventing Mukaiyama oxidation: selective S–O bond formation via sulfenamide–alcohol coupling

Guoling Huang, Huarui Zhu, Shuting Zhou, Wanlin Zheng, Fangpeng Liang, Zhibo Zhao, Yifei Chen and Xunbo Lu

Beilstein J. Org. Chem. **2026,** *22,* 158–166. doi:10.3762/bjoc.22.9

Experimental procedures, characterization data and copies of spectra

License and Terms: This is a supporting information file under the terms of the Creative Commons Attribution License (https://creativecommons.org/licenses/by/4.0). Please note that the reuse, redistribution and reproduction in particular requires that the author(s) and source are credited and that individual graphics may be subject to special legal provisions.
The license is subject to the *Beilstein Journal of Organic Chemistry* terms and conditions: (https://www.beilstein-journals.org/bjoc/terms)

Table of contents

(A) General information S2

(B) Procedures for the synthesis of substrates S2

(C) Procedures for the synthesis of products S3

(D) Scale-up reaction S4

(E) Analytical data of products S4

(F) References S28

(G) Copies of NMR spectra (^{1}H, ^{13}C, ^{19}F) S29

(A) General information

Chemicals and solvents were purchased from commercial suppliers and used as received unless noted. All products were purified by flash chromatography on silica gel. The chemical yields referred are isolated products.^{1}H NMR and ^{13}C NMR spectra were recorded on 400 MHz Bruker spectrometers. Chemical shifts of ^{1}H NMR spectra were reported in parts per million relative to the TMS peak (δ 0.0). Chemical shifts of ^{13}C NMR spectra were reported relative to $CDCl_3$ (δ 77.16). The used abbreviations are as follows: s (singlet), d (doublet), t (triplet), quart. (quartet), quint. (quintet), m (multiplet), br (broad). Multiplets which arise from accidental equality of coupling constants of magnetically non-equivalent protons are marked as virtual (*virt.*). High-resolution mass spectra (HRMS) data were measured on an ESI-microTOF Ⅱ. Melting points were measured on a SGW® X-4B and are not corrected. HPLC analyses were performed using a chiral stationary phase (ChiralPak IA, ChiralPak IB, ChiralPak IC, UV detection, Daicel Chemical Industries) employing *n*-hexane/iPrOH as eluents. Reactions were monitored by TLC analysis using silica gel 60 Å GF-254 thin layer plates and compounds were visualized with a UV light at 254 nm or 365 nm. Further visualization was achieved by staining with iodine, or $KMnO_4$ followed by heating on a hot plate. Flash column chromatography was performed on silica gel 60 Å, 10–40 μm.
The substrates were prepared following literature procedures [1-3] which are provided in section B.

(B) Procedures for the synthesis of substrates

The sulfenamides were prepared following the literature procedure reported by our groups.[1]
General scheme for the two-step synthesis of sulfenamides from *N*-thiosuccinimides.

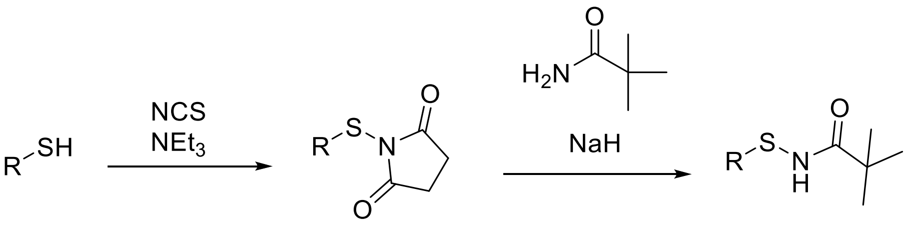

General procedure for the synthesis of *N*-thiosuccinimides: To a stirred solution of the indicated thiol (5.0 mmol,1.0 equiv) in DCM (0.25 M) under nitrogen was added *N*-chlorosuccinimide at room temperature. After stirring at room temperature for one hour, a solution of triethylamine (5.0 mmol,1.0 equiv) in DCM (0.7 M) was added dropwise over 30 minutes. The reaction mixture was heated to 40 °C overnight. After cooling to room temperature, the reaction mixture was diluted with H_2O, and extracted 3× with ethyl acetate. The combined organic layers were washed with brine, dried over Na_2SO_4, and concentrated. The crude residue was purified through column chromatography.

General procedure for the synthesis of sulfenamides: To a flame-dried round-bottomed flask under nitrogen and equipped with an addition funnel and stirring bar was added pivalamide (1.0 mmol,1.0 equiv). THF (0.15 M) was added and the solution was cooled to 0 °C. NaH (2.5 equiv) was added, then the solution was warmed to room temperature and stirred for 30 minutes. The addition funnel was charged with a solution of the indicated thiosuccinimide (1.0 mmol, 1.0 equiv) in THF (0.3 M), which was added dropwise over one hour. Upon completion of the addition, the reaction mixture was stirred at room temperature for 30 minutes, then quenched with saturated aqueous ammonium chloride and extracted 3× with ethyl acetate. The combined organic layers were washed with brine, dried over Na_2SO_4, and concentrated. The crude residue was purified through column chromatography.

(C) Procedures for the synthesis of products

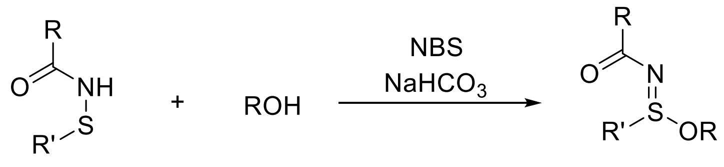

1 **2** **3**

General procedure 1: A solution of sulfenamides **1** (0.15 mmol) and $NaHCO_3$ (0.225 mmol, 1.5 equiv) was prepared in ROH **2** (1.5 mL). To this, NBS (0.18 mmol, 1.2 equiv) was added at room temperature. The mixture was stirred at room temperature for 0.5 hours. Following this, the reaction was quenched using a saturated NH_4Cl solution. The resulting mixture was then extracted with ethyl acetate (EtOAc). The combined organic phases were subsequently washed with brine, dried over Na_2SO_4, and the solvents were evaporated. The residual crude material was then subjected to column chromatography, yielding the desired product.

General procedure 2: A solution of sulfenamides **1** (0.15 mmol), ROH **2** (10 equiv) and $NaHCO_3$ (0.225 mmol, 1.5 equiv) was prepared in DCM (1.5 mL). To this, NBS (0.18 mmol, 1.2 equiv) was added at room temperature. The mixture was stirred at room temperature for 0.5 hours. Following this, the reaction was quenched using a saturated NH_4Cl solution. The resulting mixture was then extracted with ethyl acetate (EtOAc). The combined organic phases were subsequently washed with brine, dried over Na_2SO_4, and the solvents were evaporated. The residual crude material was then subjected to column chromatography, yielding the desired product.

(D) Scale-up reaction

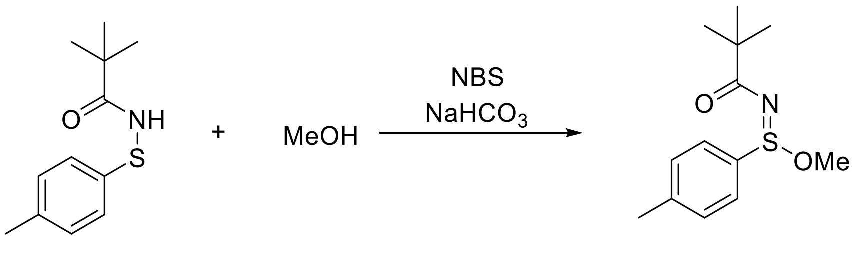

A solution of sulfenamides **1a** (670.0 mg, 3 mmol) and $NaHCO_3$ (378.0 mg, 1.5 equiv, 4.5 mmol) was prepared in MeOH (25 mL). To this, NBS (640.7 mg, 1.2 equiv, 3.6 mmol) was added at room temperature. The mixture was stirred at room temperature for 1.0 hour. The reaction was quenched with saturated aqueous NH_4Cl, and the resulting mixture was extracted with EtOAc (3 × 20 mL). The combined organic layers were washed with brine, dried over anhydrous Na_2SO_4, filtered, and concentrated under reduced pressure. The crude product was purified by column chromatography to afford compound **3a** (623.3 mg, 82% yield).

(E) Analytical data of products

Methyl *N*–(pivaloyl)-4-methylphenylsulfinimidate (3a)

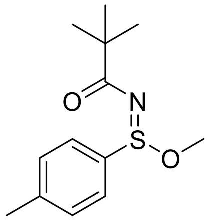

This compound was synthesized following *General procedure 1*, in which MeOH served as both the solvent and the alcohol reactant.

A colorless oil, 37.6 mg, 99% yield.

TLC: R_f = 0.52 (PE/EA = 3:1).

^{1}H NMR (400 MHz, $CDCl_3$) δ 7.79 – 7.69 (m, 2H), 7.33 – 7.23 (m, 2H), 3.47 (s, 3H), 2.37 (s, 3H), 1.23 (s, 9H).

^{13}C NMR (101 MHz, $CDCl_3$) δ 193.0, 143.6, 133.0, 130.1, 128.0, 51.8, 41.3, 28.4, 21.7. Analytical data in agreement with the literature.[1]

Methyl *N*–(pivaloyl)phenylsulfinimidate (3b)

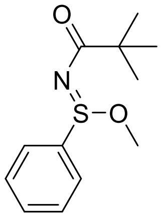

This compound was synthesized following *General procedure 1*, in which MeOH served as both the solvent and the alcohol reactant.

A colorless oil, 34.8 mg, 97% yield.

TLC: R_f = 0.65 (PE/EA = 3:1).

^{1}H NMR (400 MHz, $CDCl_3$) δ 7.91 – 7.84 (m, 2H), 7.57 – 7.45 (m, 3H), 3.50 (s, 3H), 1.24 (s, 9H). Analytical data in agreement with the literature.[1]

Methyl *N*–(pivaloyl)-4-methoxyphenylsulfinimidate (3c)

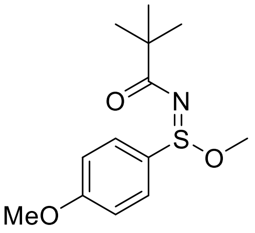

This compound was synthesized following *General procedure 1*, in which MeOH served as both the solvent and the alcohol reactant.

A colorless oil, 34.3 mg, 85% yield.

TLC: R_f = 0.42 (PE/EA = 3:1).

^{1}H NMR (400 MHz, $CDCl_3$) δ 7.85 – 7.78 (m, 2H), 7.01 – 6.95 (m, 2H), 3.81 (s, 3H), 3.45 (s, 3H), 1.23 (s, 9H).

^{13}C NMR (101 MHz, $CDCl_3$) δ 192.8, 163.3, 130.0, 127.2, 114.8, 55.8, 51.5, 41.3, 28.4.

HRMS (ESI+): m/z calcd for $C_{13}H_{19}NO_3SNa^+$ $[M+Na]^+$:292.0978, found: 292.0977. Analytical data in agreement with the literature.[1]

Methyl *N*–(pivaloyl)-4-*tert*-butylphenylsulfinimidate (3d)

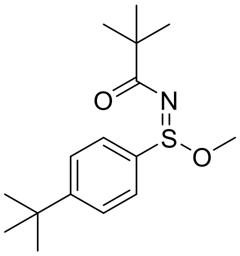

This compound was synthesized following *General procedure 1*, in which MeOH served as both the solvent and the alcohol reactant.

A colorless oil, 38.1 mg, 86% yield.

TLC: R_f = 0.52 (PE/EA = 3:1).

^{1}H NMR (400 MHz, $CDCl_3$) δ 7.91 – 7.85 (m, 2H), 7.63 – 7.57 (m, 2H), 3.59 (s, 3H), 1.37 (s, 9H), 1.32 (s, 9H).

^{13}**C NMR** (101 MHz, $CDCl_3$) δ 193.0, 156.6, 133.0, 127.9, 126.5, 52.1, 41.3, 35.3, 31.3, 28.4.
HRMS (ESI+): m/z calcd for $C_{16}H_{25}NO_2SNa^+$ $[M+Na]^+$:318.1498, found: 318.1498.

Methyl *N*–(pivaloyl)-4-fluorohenylsulfinimidate (3e)

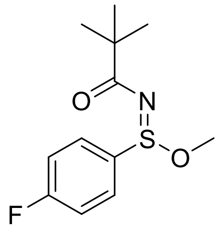

This compound was synthesized following *General procedure 1*, in which MeOH served as both the solvent and the alcohol reactant.
A colorless oil, 32.0 mg, 83% yield.
TLC: R_f = 0.66 (PE/EA = 3:1).
^{1}**H NMR** (400 MHz, $CDCl_3$) δ 7.93 – 7.85 (m, 2H), 7.23 – 7.14 (m, 2H), 3.50 (s, 3H), 1.23 (s, 9H). Analytical data in agreement with the literature.[1]

Methyl *N*–(pivaloyl)-4-chlorohenylsulfinimidate (3f)

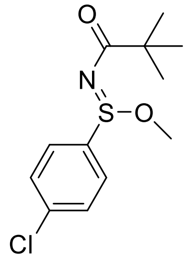

This compound was synthesized following *General procedure 1*, in which MeOH served as both the solvent and the alcohol reactant.
A colorless oil, 36.5 mg, 89% yield.
TLC: R_f = 0.63 (PE/EA = 3:1).
^{1}**H NMR** (400 MHz, $CDCl_3$) δ 7.86 – 7.77 (m, 2H), 7.50 – 7.45 (m, 2H), 3.51 (s, 3H), 1.23 (s, 9H). Analytical data in agreement with the literature.[1]

Methyl *N*–(pivaloyl)-4-brophenylsulfinimidate (3g)

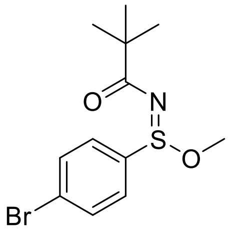

This compound was synthesized following *General procedure 1*, in which MeOH served as both the solvent and the alcohol reactant.
A colorless oil, 39.1 mg, 82% yield.
TLC: R_f = 0.55 (PE/EA = 3:1).
^{1}**H NMR** (400 MHz, $CDCl_3$) δ 7.77 – 7.71 (m, 2H), 7.66 – 7.60 (m, 2H), 3.51 (s, 3H), 1.23 (s, 9H). Analytical data in agreement with the literature.[1]

Methyl *N*–(pivaloyl)-3-methylphenylsulfinimidate (3h)

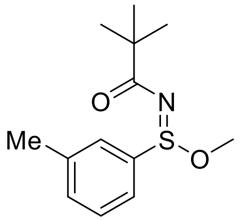

This compound was synthesized following *General procedure 1*, in which MeOH served as both the solvent and the alcohol reactant.

A colorless oil, 29.3 mg, 77% yield.

TLC: R_f = 0.65 (PE/EA = 3:1).

^{1}H NMR (400 MHz, $CDCl_3$) δ 7.71 – 7.61 (m, 2H), 7.41 – 7.29 (m, 2H), 3.50 (s, 3H), 2.39 (s, 3H), 1.24 (s, 9H). Analytical data in agreement with the literature.[1]

Methyl *N*–(pivaloyl)-3-methoxyphenylsulfinimidate (3i)

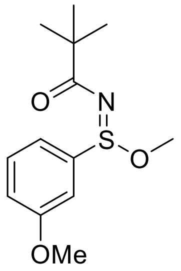

This compound was synthesized following *General procedure 1*, in which MeOH served as both the solvent and the alcohol reactant.

A colorless oil, 36.8 mg, 91% yield.

TLC: R_f = 0.65 (PE/EA = 3:1).

^{1}H NMR (400 MHz, $CDCl_3$) δ 7.47 – 7.44 (m, 1H), 7.42 – 7.38 (m, 2H), 7.08 – 7.02 (m, 1H), 3.81 (s, 3H), 3.49 (s, 3H), 1.24 (s, 9H). Analytical data in agreement with the literature.[1]

Methyl *N*–(pivaloyl)-3-fluorophenylsulfinimidate (3j)

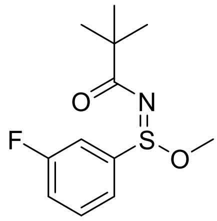

This compound was synthesized following *General procedure 1*, in which MeOH served as both the solvent and the alcohol reactant.

A colorless oil, 34.7 mg, 90% yield.

TLC: R_f = 0.68 (PE/EA = 3:1).

^{1}H NMR (400 MHz, $CDCl_3$) δ 7.68 – 7.59 (m, 2H), 7.53 – 7.45 (m, 1H), 7.27 – 7.20 (m, 1H), 3.52 (s, 3H), 1.24 (s, 9H). Analytical data in agreement with the literature.[1]

Methyl *N*–(pivaloyl)-3-bromophenylsulfinimidate (3k)

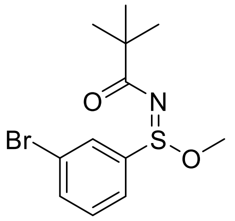

This compound was synthesized following *General procedure 1*, in which MeOH served as both the solvent and the alcohol reactant.

A colorless oil, 36.8 mg, 77% yield.

TLC: R_f = 0.55 (PE/EA = 3:1).

^{1}H NMR (400 MHz, $CDCl_3$) δ 7.77 – 7.72 (m, 2H), 7.66 – 7.61 (m, 2H), 3.51 (s, 3H), 1.23 (s, 9H). Analytical data in agreement with the literature. [1]

Methyl *N*–(pivaloyl)-2-methylphenylsulfinimidate (3l)

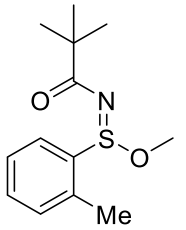

This compound was synthesized following *General procedure 1*, in which MeOH served as both the solvent and the alcohol reactant.

A colorless oil, 34.6 mg, 91% yield.

TLC: R_f = 0.65 (PE/EA = 3:1).

^{1}H NMR (400 MHz, $CDCl_3$) δ 8.42 – 8.35 (m, 1H), 7.54 – 7.48 (m, 1H), 7.46 – 7.39 (m, 1H), 7.36 – 7.31 (m, 1H), 3.58 (s, 3H), 2.61 (s, 3H), 1.32 (s, 5H). Analytical data in agreement with the literature.[1]

Methyl *N*–(pivaloyl)-2-fluorophenylsulfinimidate (3m)

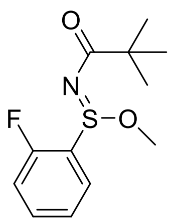

This compound was synthesized following *General procedure 1*, in which MeOH served as both the solvent and the alcohol reactant.

A colorless oil, 35.5 mg, 92% yield.

TLC: R_f = 0.73 (PE/EA = 3:1).

^{1}H NMR (400 MHz, $CDCl_3$) δ 8.27 – 8.20 (m, 1H), 7.57 – 7.50 (m, 1H), 7.34 – 7.27 (m, 1H), 7.19 – 7.11 (m, 1H), 3.66 (s, 3H), 1.23 (s, 9H). Analytical data in agreement with the literature.[1]

Methyl *N*–(pivaloyl)-2-chlorophenylsulfinimidate (3n)

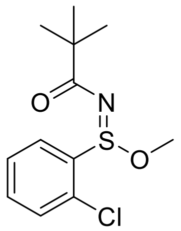

This compound was synthesized following *General procedure 1*, in which MeOH served as both the solvent and the alcohol reactant.

A colorless oil, 35.7 mg, 87% yield.

TLC: R_f = 0.67 (PE/EA = 3:1).

^{1}H NMR (400 MHz, $CDCl_3$) δ 8.51 – 8.44 (m, 1H), 7.60 – 7.48 (m, 3H), 3.81 (s, 3H), 1.31 (s, 9H). Analytical data in agreement with the literature.[1]

Methyl *N*–(pivaloyl)-2-bromophenylsulfinimidate (3o)

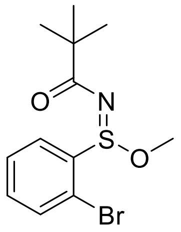

This compound was synthesized following *General procedure 1*, in which MeOH served as both the solvent and the alcohol reactant.

A colorless oil, 43.4 mg, 91% yield.

TLC: R_f = 0.78 (PE/EA = 3:1).

^{1}H NMR (400 MHz, $CDCl_3$) δ 8.40 – 8.34 (m, 1H), 7.66 – 7.59 (m, 1H), 7.50 – 7.45 (m, 1H), 7.42 – 7.35 (m, 1H), 3.74 (s, 3H), 1.22 (s, 9H). Analytical data in agreement with the literature.[1]

Methyl *N*–(pivaloyl)-2-naphthylsulfinimidate (3p)

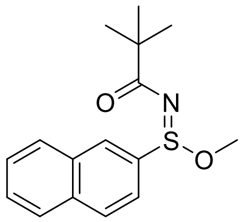

This compound was synthesized following *General procedure 1*, in which MeOH served as both the solvent and the alcohol reactant.

A colorless oil, 30.4 mg, 70% yield.

TLC: R_f = 0.65 (PE/EA = 3:1).

^{1}H NMR (400 MHz, $CDCl_3$) δ 8.43 – 8.38 (m, 1H), 7.96 – 7.89 (m, 2H), 7.88 – 7.81 (m, 2H), 7.61 – 7.51 (m, 2H), 3.52 (s, 3H), 1.28 (s, 9H).

^{13}C NMR (101 MHz, $CDCl_3$) δ 193.0, 135.2, 133.3, 132.7, 129.7, 129.4, 129.3, 128.9, 128.1, 127.6, 123.1, 52.2, 41.4, 28.4.

HRMS (ESI+): m/z calcd for $C_{16}H_{19}NO_2SNa^+$ $[M+Na]^+$:312.1029, found: 312.1029.

Methyl *N*–(pivaloyl)-cyclohexylsulfinimidate (3q)

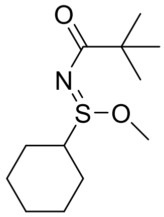

This compound was synthesized following *General procedure 1*, in which MeOH served as both the solvent and the alcohol reactant.

A colorless oil, 25.8 mg, 70% yield.

TLC: R_f = 0.68 (PE/EA = 3:1).

^{1}H NMR (400 MHz, $CDCl_3$) δ 3.82 (s, 3H), 3.00–2.88 (m, 1H), 2.10–1.95 (m, 2H), 1.88–1.74 (m, 2H), 1.67–1.59 (m, 1H), 1.46–1.21 (m, 5H), 1.15 (s, 9H). Analytical data in agreement with the literature.[1]

Methyl *N*–(pivaloyl)-hexylsulfinimidate (3r)

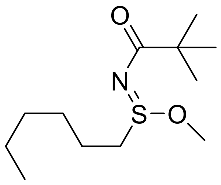

This compound was synthesized following *General procedure 1*, in which MeOH served as both the solvent and the alcohol reactant.

A colorless oil, 26.0 mg, 70% yield.

TLC: R_f = 0.67 (PE/EA = 3:1).

^{1}H NMR (400 MHz, $CDCl_3$) δ 3.82 (s, 3H), 3.05–2.87 (m, 2H), 1.71–1.59 (m, 2H), 1.41–1.32 (m, 2H), 1.28–1.22 (m, 4H), 1.14 (s, 9H), 0.87–0.79 (m, 4H). Analytical data in agreement with the literature.[1]

Methyl *N*–(pivaloyl)-thiophensulfinimidate (3s)

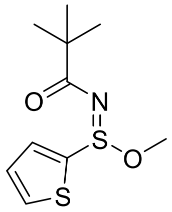

This compound was synthesized following *General procedure 1*, in which MeOH served as both the solvent and the alcohol reactant.

A colorless oil, 36.1 mg, 98% yield.

TLC: R_f = 0.53 (PE/EA = 3:1).

^{1}H NMR (400 MHz, $CDCl_3$) δ 7.76 – 7.70 (m, 1H), 7.65 – 7.59 (m, 1H), 7.27 – 7.19 (m, 1H), 3.55 (s, 3H), 1.30 (s, 9H). Analytical data in agreement with the literature.[1]

Methyl *N*–(acetyl)-4-methylphenylsulfinimidate (3t)

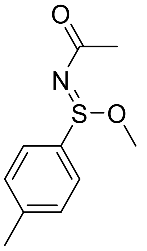

This compound was synthesized following *General procedure 1*, in which MeOH served as both the solvent and the alcohol reactant.

A colorless oil, 28.8 mg, 91% yield.

TLC: R_f = 0.29 (PE/EA = 3:1).

^{1}H NMR (400 MHz, $CDCl_3$) δ 7.76–7.69 (m, 2H), 7.33–7.27 (m, 2H), 3.52 (s, 3H), 2.37 (s, 3H), 2.19 (s, 3H).

^{13}C NMR (101 MHz, $CDCl_3$) δ 185.0, 143.9, 132.3, 130.3, 127.9, 52.6, 25.9, 21.7. Analytical data in agreement with the literature. [1]

Methyl *N*–(isobutyryl)-4-methylphenylsulfinimidate (3u)

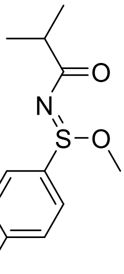

This compound was synthesized following *General procedure 1*, in which MeOH served as both the solvent and the alcohol reactant.

A colorless oil, 28.7 mg, 80% yield.

TLC: R_f = 0.54 (PE/EA = 3:1).

^{1}H NMR (400 MHz, $CDCl_3$) δ 7.85–7.80 (m, 2H), 7.41–7.35 (m, 2H), 3.60 (s, 3H), 2.86–2.70 (m, 1H), 2.46 (s, 3H), 1.27 (d, J = 2.8 Hz, 3H), 1.25 (d, J = 2.9 Hz, 3H). Analytical data in agreement with the literature.[1]

Methyl *N*–(cyclopropanecarbonyl)-4-methylphenylsulfinimidate (3v)

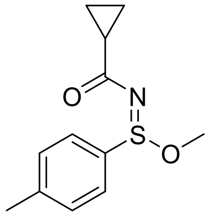

This compound was synthesized following *General procedure 1*, in which MeOH served as both the solvent and the alcohol reactant.

A colorless oil, 28.8 mg, 81% yield.

TLC: R_f = 0.36 (PE/EA = 3:1).

^{1}H NMR (400 MHz, $CDCl_3$) δ 7.79 – 7.70 (m, 2H), 7.35 – 7.25 (m, 2H), 3.52 (s, 3H), 2.37 (s, 3H), 1.92 – 1.80 (m, 1H), 1.10 – 0.92 (m, 2H), 0.83 – 0.70 (m, 2H). Analytical data in agreement with the literature.[1]

Methyl *N*–(cyclohexanecarbonyl)-4-methylphenylsulfinimidate (3w)

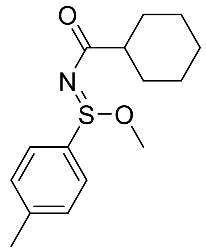

This compound was synthesized following *General procedure 1*, in which MeOH served as both the solvent and the alcohol reactant.

A colorless oil, 36.0 mg, 86% yield.

TLC: R_f = 0.57 (PE/EA = 3:1).

^{1}H NMR (400 MHz, $CDCl_3$) δ 7.86 – 7.78 (m, 2H), 7.41 – 7.35 (m, 2H), 3.58 (s, 3H), 2.54 – 2.46 (m, 1H), 2.45 (s, 3H), 2.09 – 1.98 (m, 2H), 1.85 – 1.75 (m, 2H), 1.72 – 1.65 (m, 1H), 1.61 – 1.48 (m, 2H), 1.40 – 1.20 (m, 4H). Analytical data in agreement with the literature.[1]

Methyl *N*–(adamantane-1-carbonyl)-4-methylphenylsulfinimidate (3x)

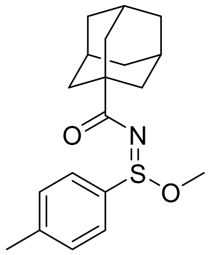

This compound was synthesized following *General procedure 1*, in which MeOH served as both the solvent and the alcohol reactant.

A colorless oil, 43.8 mg, 88% yield.

TLC: R_f = 0.64 (PE/EA = 3:1).

^{1}H NMR (400 MHz, $CDCl_3$) δ 7.87 – 7.80 (m, 2H), 7.41 – 7.35 (m, 2H), 3.55 (s, 3H), 2.46 (s, 3H), 2.09 – 2.01 (m, 9H), 1.79 – 1.73 (m, 6H). Analytical data in agreement with the literature.[1]

Methyl *N*–[2-(naphthalen-1-yl)acetyl]-4-methylphenylsulfinimidate (3y)

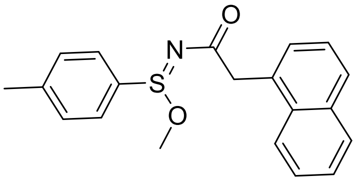

This compound was synthesized following *General procedure 1*, in which MeOH served as both the solvent and the alcohol reactant.

A colorless oil, 50.1 mg, 99% yield.

TLC: R_f = 0.38 (PE/EA = 3:1).

^{1}H NMR (400 MHz, $CDCl_3$) δ 8.16 – 8.11 (m, 1H), 7.79 – 7.74 (m, 1H), 7.70 – 7.66 (m, 1H), 7.57 – 7.50 (m, 2H), 7.44 – 7.40 (m, 2H), 7.40 – 7.32 (m, 2H), 7.21 – 7.16 (m, 3H), 4.20 (s, 2H), 3.38 (s, 3H), 2.32 (s, 3H). Analytical data in agreement with the literature.[1]

Methyl *N*–(benzoyl)-4-methylphenylsulfinimidate (3z)

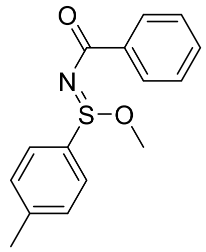

This compound was synthesized following *General procedure 1*, in which MeOH served as both the solvent and the alcohol reactant.

A colorless oil, 32.8 mg, 80% yield.

TLC: R_f = 0.51 (PE/EA = 3:1).

^{1}H NMR (400 MHz, $CDCl_3$) δ 8.27 – 8.19 (m, 2H), 7.90 – 7.84 (m, 2H), 7.48 – 7.41 (m, 1H), 7.40 – 7.32 (m, 4H), 3.56 (s, 3H), 2.40 (s, 3H). Analytical data in agreement with the literature.[1]

Methyl *N*–(2-chlorobenzoyl)-4-methylphenylsulfinimidate (3a')

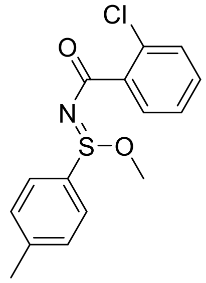

This compound was synthesized following *General procedure 1*, in which MeOH served as both the solvent and the alcohol reactant.

A colorless oil, 40.2 mg, 87% yield.

TLC: R_f = 0.43 (PE/EA = 3:1).

^{1}H NMR (400 MHz, $CDCl_3$) δ 7.96 – 7.86 (m, 3H), 7.47 – 7.43 (m, 1H), 7.42 – 7.39 (m, 2H), 7.38 – 7.29 (m, 2H), 3.68 (s, 3H), 2.47 (s, 3H).

^{13}C NMR (101 MHz, $CDCl_3$) δ 178.9, 144.2, 136.9, 132.4, 131.9, 130.9, 130.9, 130.6, 130.3, 128.2, 126.5, 52.6, 21.7.

HRMS (ESI+): m/z calcd for $C_{15}H_{14}ClNO_2SNa^+$ $[M+Na]^+$: 330.0326, found: 330.0326.

Methyl *N*–(2-bromobenzoyl)-4-methylphenylsulfinimidate (3b')

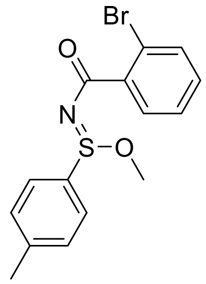

This compound was synthesized following *General procedure 1*, in which MeOH served as both the solvent and the alcohol reactant.

A colorless oil, 46.5 mg, 88% yield.

TLC: R_f = 0.37 (PE/EA = 3:1).

^{1}H NMR (400 MHz, $CDCl_3$) δ 7.96 – 7.89 (m, 2H), 7.88 – 7.82 (m, 1H), 7.67 – 7.62 (m, 1H), 7.44 – 7.39 (m, 2H), 7.39 – 7.33 (m, 1H), 7.28 – 7.22 (m, 1H), 3.69 (s, 3H), 2.47 (s, 3H).
^{13}C NMR (101 MHz, $CDCl_3$) δ 179.5, 144.2, 139.0, 133.8, 131.8, 131.0, 130.7, 130.3, 128.2, 127.1, 120.6, 52.7, 21.7.
HRMS (ESI+): m/z calcd for $C_{15}H_{14}{}^{81}BrNO_2SNa^+$ $[M+Na]^+$: 375.9800, found: 375.9801.
HRMS (ESI+): m/z calcd for $C_{15}H_{14}{}^{79}BrNO_2SNa^+$ $[M+Na]^+$: 373.9821, found: 373.9823.

Methyl *N*–(3-chlorobenzoyl)-4-methylphenylsulfinimidate (3c')

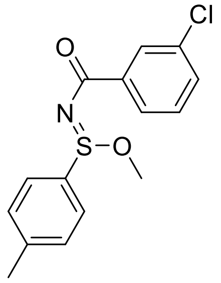

This compound was synthesized following *General procedure 1*, in which MeOH served as both the solvent and the alcohol reactant.
A colorless oil, 43.9 mg, 95% yield.
TLC: R_f = 0.55 (PE/EA = 3:1).
^{1}H NMR (400 MHz, $CDCl_3$) δ 8.29 (t, J = 1.8 Hz, 1H), 8.20 – 8.15 (m, 1H), 7.99 – 7.89 (m, 2H), 7.52 – 7.48 (m, 1H), 7.47 – 7.42 (m, 2H), 7.42 – 7.36 (m, 1H), 3.65 (s, 3H), 2.49 (s, 3H).
^{13}C NMR (101 MHz, $CDCl_3$) δ 177.1, 144.3, 137.8, 134.1, 132.2, 131.8, 130.4, 129.7, 129.4, 128.1, 127.7, 52.5, 21.7.
HRMS (ESI+): m/z calcd for $C_{15}H_{14}ClNO_2SNa^+$ $[M+Na]^+$: 330.0326, found: 330.0327.

Methyl *N*–(3-bromobenzoyl)-4-methylphenylsulfinimidate (3d')

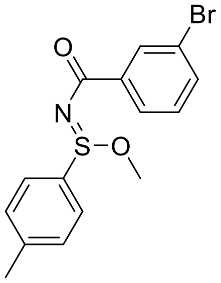

This compound was synthesized following *General procedure 1*, in which MeOH served as both the solvent and the alcohol reactant.
A colorless oil, 42.8 mg, 81% yield.
TLC: R_f = 0.54 (PE/EA = 3:1).
^{1}H NMR (400 MHz, $CDCl_3$) δ 8.48 – 8.41 (m, 1H), 8.27 – 8.18 (m, 1H), 7.99 – 7.89 (m, 2H), 7.69 – 7.61 (m, 1H), 7.49 – 7.41 (m, 2H), 7.38 – 7.30 (m, 1H), 3.65 (s, 3H), 2.49 (s, 3H).
^{13}C NMR (101 MHz, $CDCl_3$) δ 176.9, 144.3, 138.0, 134.7, 132.6, 132.2, 130.4, 129.7, 128.1, 128.1, 122.2, 52.5, 21.7.
HRMS (ESI+): m/z calcd for $C_{15}H_{14}{}^{81}BrNO_2SNa^+$ $[M+Na]^+$: 375.9800, found: 375.9800.
HRMS (ESI+): m/z calcd for $C_{15}H_{14}{}^{79}BrNO_2SNa^+$ $[M+Na]^+$: 373.9821, found: 373.9822.

Methyl *N*–(4-chlorobenzoyl)-4-methylphenylsulfinimidate (3e')

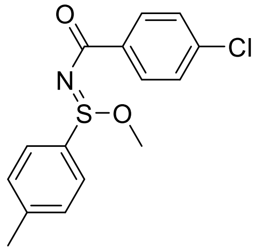

This compound was synthesized following *General procedure 1*, in which MeOH served as both the solvent and the alcohol reactant.

A colorless oil, 36.9 mg, 80% yield.

TLC: R_f = 0.50 (PE/EA = 3:1).

^{1}**H NMR** (400 MHz, $CDCl_3$) δ 8.34 – 8.16 (m, 2H), 7.99 – 7.87 (m, 2H), 7.47 – 7.38 (m, 4H), 3.65 (s, 3H), 2.49 (s, 3H).

^{13}**C NMR** (101 MHz, $CDCl_3$) δ 177.4, 144.2, 138.1, 134.4, 132.4, 131.0, 130.3, 128.3, 128.0, 52.5, 21.7.

HRMS (ESI+): m/z calcd for $C_{15}H_{14}ClNO_2SNa^+$ $[M+Na]^+$: 330.0326, found: 330.0326.

Methyl *N*–(4-bromobenzoyl)-4-methylphenylsulfinimidate (3f')

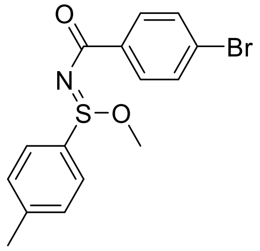

This compound was synthesized following *General procedure 1*, in which MeOH served as both the solvent and the alcohol reactant.

A colorless oil, 47.6 mg, 90% yield.

TLC: R_f = 0.41 (PE/EA = 3:1).

^{1}**H NMR** (400 MHz, $CDCl_3$) δ 8.12 – 8.05 (m, 1H), 7.88 – 7.81 (m, 1H), 7.52 – 7.47 (m, 1H), 7.39 – 7.31 (m, 3H), 3.56 (s, 3H), 2.40 (s, 3H).

^{13}**C NMR** (101 MHz, $CDCl_3$) δ 177.6, 144.2, 134.9, 132.4, 131.3, 131.2, 130.3, 128.1, 126.8, 52.5, 21.7.

HRMS (ESI+): m/z calcd for $C_{15}H_{14}{}^{81}BrNO_2SNa^+$ $[M+Na]^+$: 375.9800, found: 375.9800.

HRMS (ESI+): m/z calcd for $C_{15}H_{14}{}^{79}BrNO_2SNa^+$ $[M+Na]^+$: 373.9821, found: 373.9821.

Methyl *N*–(4-methoxybenzoyl)-4-methylphenylsulfinimidate (3g')

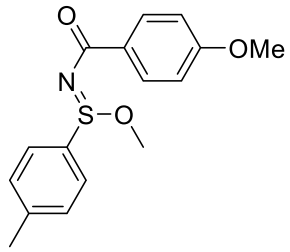

This compound was synthesized following *General procedure 1*, in which MeOH served as both the solvent and the alcohol reactant.

A colorless oil, 45.0 mg, 99% yield.

TLC: R_f = 0.39 (PE/EA = 3:1).

^{1}H NMR (400 MHz, $CDCl_3$) δ 8.32 – 8.25 (m, 2H), 7.98 – 7.91 (m, 2H), 7.45 – 7.39 (m, 2H), 6.99 – 6.90 (m, 2H), 3.88 (s, 3H), 3.64 (s, 3H), 2.48 (s, 3H). Analytical data in agreement with the literature.[1]

Methyl *N*–(2-Naphthoyl)–4–methylphenylsulfinimidate (3h')

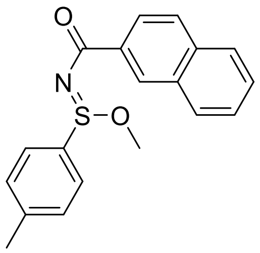

This compound was synthesized following *General procedure 1*, in which MeOH served as both the solvent and the alcohol reactant.

A colorless oil, 37.4 mg, 77% yield.

TLC: R_f = 0.38 (PE/EA = 3:1).

^{1}H NMR (400 MHz, $CDCl_3$) δ 8.83 – 8.73 (m, 1H), 8.31 – 8.24 (m, 1H), 7.95 – 7.87 (m, 3H), 7.85 – 7.76 (m, 2H), 7.50 – 7.40 (m, 2H), 7.38 – 7.31 (m, 2H), 3.60 (s, 3H), 2.40 (s, 3H).

^{13}C NMR (101 MHz, $CDCl_3$) δ 178.6, 144.0, 135.3, 133.3, 132.8, 132.7, 130.5, 130.3, 129.5, 128.1, 127.8, 127.7, 127.6, 126.3, 126.1, 52.5, 21.7.

HRMS (ESI+): m/z calcd for $C_{19}H_{17}NO_2SNa^+$ $[M+Na]^+$: 346.0872, found: 346.0872.

Methyl *N*–(cinnamoyl)-4-methylphenylsulfinimidate (3i')

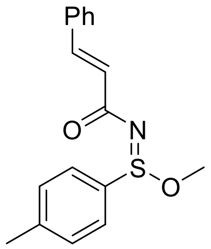

This compound was synthesized following *General procedure 1*, in which MeOH served as both the solvent and the alcohol reactant.

A colorless oil, 33.7 mg, 75% yield.

TLC: R_f = 0.52 (PE/EA = 3:1).

^{1}H NMR (400 MHz, $CDCl_3$) δ 7.99 – 7.87 (m, 2H), 7.81 (d, J = 15.9 Hz, 1H), 7.64 – 7.53 (m, 2H), 7.45 – 7.31 (m, 5H), 6.85 (d, J = 15.9 Hz, 1H), 3.67 (s, 3H), 2.48 (s, 3H). Analytical data in agreement with the literature.[1]

Methyl *N*–(*tert*-butyloxycarbonyl)–4–methylphenylsulfinimidate (3j')

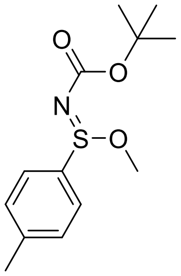

This compound was synthesized following *General procedure 1*, in which MeOH served as both the solvent and the alcohol reactant.

A colorless oil, 36.4 mg, 90% yield.

TLC: R_f = 0.63 (PE/EA = 3:1).

^{1}H NMR (400 MHz, $CDCl_3$) δ 7.87 – 7.78 (m, 2H), 7.39 – 7.32 (m, 2H), 3.51 (s, 3H), 2.44 (s, 3H), 1.55 (s, 9H).

^{13}C NMR (101 MHz, $CDCl_3$) δ 164.0, 143.8, 132.3, 130.1, 128.0, 80.4, 50.8, 28.4, 21.7.

HRMS (ESI+): m/z calcd for $C_{13}H_{19}NO_3SNa^+$ $[M+Na]^+$: 292.0978, found: 292.0978.

Methyl *N*–[(benzyloxy)carbonyl]–4–methylphenylsulfinimidate (3k')

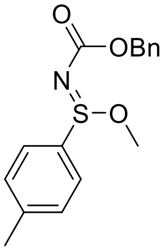

This compound was synthesized following *General procedure 1*, in which MeOH served as both the solvent and the alcohol reactant.

A colorless oil, 44.6 mg, 98% yield.

TLC: R_f = 0.50 (PE/EA = 3:1).

^{1}H NMR (400 MHz, $CDCl_3$) δ 7.49 – 7.44 (m, 2H), 7.40 – 7.34 (m, 5H), 7.34 – 7.31 (m, 1H), 5.26 (d, *J* = 12.4 Hz, 1H), 5.24 (d, *J* = 12.4 Hz, 1H), 3.51 (s, 3H), 2.45 (s, 3H). Analytical data in agreement with the literature.[1]

Methyl *N*–(pyrazine-2-carbonyl)-4-methylphenylsulfinimidate (3l')

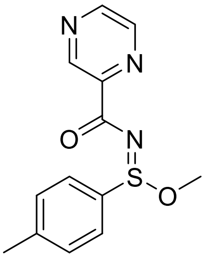

This compound was synthesized following *General procedure 1*, in which MeOH served as both the solvent and the alcohol reactant.

A colorless oil, 40.9 mg, 99% yield.

TLC: R_f = 0.59 (DCM/MeOH = 20:1).

^{1}H NMR (400 MHz, $CDCl_3$) δ 9.61 – 9.49 (m, 1H), 8.78 – 8.67 (m, 2H), 8.02 – 7.89 (m, 2H), 7.49 – 7.38 (m, 2H), 3.72 (s, 3H), 2.48 (s, 3H). Analytical data in agreement with the literature.[1]

Ethyl *N*–(pivaloyl)-4-methylphenylsulfinimidate (3m')

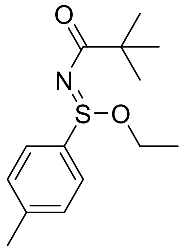

This compound was synthesized following *General procedure 1*, in which EtOH served as both the solvent and the alcohol reactant.
A colorless oil, 32.5 mg, 81% yield.
TLC: R_f = 0.33 (PE/EA = 3:1).
^{1}H NMR (400 MHz, $CDCl_3$) δ 7.79 – 7.71 (m, 2H), 7.34 – 7.21 (m, 2H), 4.28 – 4.06 (m, 1H), 3.96 – 3.70 (m, 1H), 2.37 (s, 3H), 1.22 (s, 9H). Analytical data in agreement with the literature.[1]

Propyl *N*–(pivaloyl)-4-methylphenylsulfinimidate (3n')

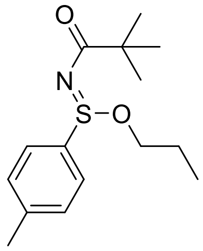

This compound was synthesized following *General procedure 1*, in which *n*-PrOH served as both the solvent and the alcohol reactant.
A colorless oil, 38.0 mg, 90% yield.
TLC: R_f = 0.87 (PE/EA = 3:1).
^{1}H NMR (400 MHz, $CDCl_3$) δ 7.90 – 7.77 (m, 2H), 7.46 – 7.31 (m, 2H), 4.13 (dt, J = 9.9, 6.6 Hz, 1H), 3.79 (dt, J = 9.9, 6.6 Hz, 1H), 2.45 (s, 3H), 1.71 – 1.55 (m, 2H), 1.31 (s, 9H), 0.89 (t, J = 7.4 Hz, 3H). Analytical data in agreement with the literature.[1]

Butyl *N*–(pivaloyl)-4-methylphenylsulfinimidate (3o')

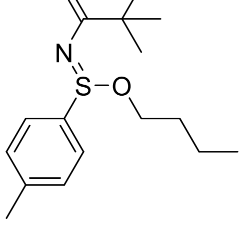

This compound was synthesized following *General procedure 1*, in which *n*-BuOH served as both the solvent and the alcohol reactant.
A colorless oil, 39.4 mg, 89% yield.

TLC: R_f = 0.83 (PE/EA = 3:1).
^{1}H NMR (400 MHz, $CDCl_3$) δ 7.85 – 7.80 (m, 2H), 7.39 – 7.34 (m, 2H), 4.17 (dt, J = 9.9, 6.6 Hz, 1H), 3.82 (dt, J = 9.9, 6.6 Hz, 1H), 2.46 (s, 3H), 1.66 – 1.56 (m, 2H), 1.31 (s, 9H), 0.88 (t, J = 7.4 Hz, 3H). Analytical data in agreement with the literature.[1]

Octyl *N*–(pivaloyl)-4-methylphenylsulfinimidate (3p')

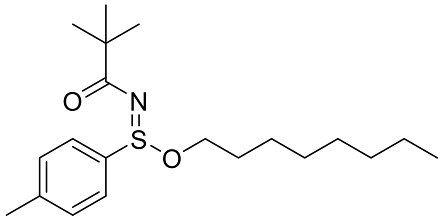

This compound was synthesized following *General procedure 2*, with 2.0 equiv of 1-octanol as the alcohol substrate.
A colorless oil, 36.9 mg, 70% yield.
TLC: R_f = 0.85 (PE/EA = 3:1).
^{1}H NMR (400 MHz, $CDCl_3$) δ 7.81 – 7.69 (m, 2H), 7.33 – 7.22 (m, 2H), 4.07 (s, 1H), 3.77 – 3.68 (m, 1H), 2.37 (s, 3H), 1.58 – 1.48 (m, 2H), 1.22 (s, 9H), 1.21 – 1.04 (m, 10H), 0.79 (t, J = 6.8 Hz, 3H). Analytical data in agreement with the literature.[1]

3-Phenylpropyl *N*–(pivaloyl)-4-methylphenylsulfinimidate (3q')

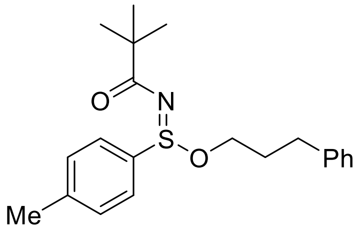

This compound was synthesized following *General procedure 2*, with 10.0 equiv of 3-phenyl-1-propanol as the alcohol substrate.
A colorless oil, 40.8 mg, 76% yield.
TLC: R_f = 0.76 (PE/EA = 3:1).
^{1}H NMR (400 MHz, $CDCl_3$) δ 7.78 – 7.71 (m, 2H), 7.31 – 7.25 (m, 2H), 7.19 – 7.15 (m, 2H), 7.13 – 7.06 (m, 1H), 7.06 – 7.01 (m, 2H), 4.13 – 4.04 (m, 1H), 3.76 (dt, J = 10.0, 6.3 Hz, 1H), 2.58 – 2.52 (m, 2H), 2.37 (s, 3H), 1.90 – 1.81 (m, 2H), 1.22 (s, 9H). Analytical data in agreement with the literature.[1]

Phenethyl *N*–(pivaloyl)-4-methylphenylsulfinimidate (3r')

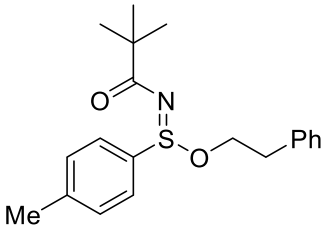

This compound was synthesized following *General procedure 2*, with 10.0 equiv of phenethyl alcohol as the alcohol substrate.
A colorless oil, 42.8 mg, 83% yield.

TLC: R_f = 0.80 (PE/EA = 3:1).
^{1}H NMR (400 MHz, $CDCl_3$) δ 7.59 – 7.52 (m, 2H), 7.22 – 7.14 (m, 6H), 7.10 – 7.04 (m, 2H), 4.34 – 4.26 (m, 1H), 4.00 – 3.92 (m, 1H), 2.84 (t, J = 7.0 Hz, 2H), 2.34 (s, 3H), 1.22 (s, 9H). Analytical data in agreement with the literature.[1]

Isopropyl *N*–(pivaloyl)-4-methylphenylsulfinimidate (3s')

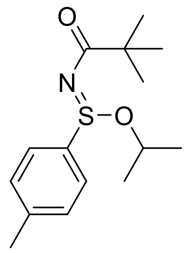

This compound was synthesized following *General procedure 2*, with 10.0 equiv of iPrOH as the alcohol substrate.
A colorless oil, 33.3 mg, 79% yield.
TLC: R_f = 0.83 (PE/EA = 3:1).
^{1}H NMR (400 MHz, $CDCl_3$) δ 7.91 – 7.79 (m, 2H), 7.44 – 7.33 (m, 2H), 5.05 – 4.85 (m, 1H), 2.45 (s, 2H), 1.41 (d, J = 6.3 Hz, 3H), 1.30 (s, 9H), 1.22 (d, J = 6.3 Hz, 3H).
^{13}C NMR (101 MHz, $CDCl_3$) δ 193.1, 143.1, 134.8, 130.0, 127.7, 75.3, 41.0, 28.4, 23.8, 23.5, 21.6.
Analytical data in agreement with the literature.[1]

Cyclobutylmethyl *N*–(pivaloyl)-4-methylphenylsulfinimidate (3t')

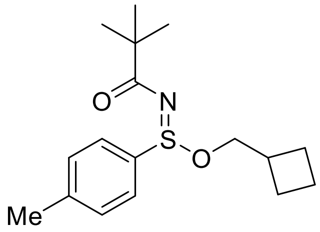

This compound was synthesized following *General procedure 2*, with 10.0 equiv of cyclobutanemethanol as the alcohol substrate.
A colorless oil, 25.4 mg, 55% yield.
TLC: R_f = 0.56 (PE/EA = 3:1).
^{1}H NMR (400 MHz, $CDCl_3$) δ 7.76 – 7.70 (m, 2H), 7.31 – 7.24 (m, 2H), 4.10 – 4.04 (m, 1H), 3.72 – 3.65 (m, 1H), 2.56 – 2.46 (m, 1H), 2.37 (s, 3H), 2.00 – 1.87 (m, 2H), 1.85 – 1.71 (m, 2H), 1.69 – 1.56 (m, 2H), 1.23 (s, 9H). Analytical data in agreement with the literature.[1]

Cyclopentylmethyl *N*–(pivaloyl)-4-methylphenylsulfinimidate (3u')

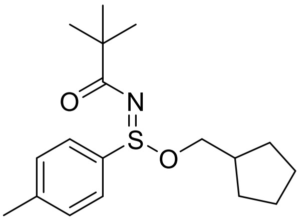

This compound was synthesized following *General procedure 2*, with 10.0 equiv of cyclopentanemethanol as the alcohol substrate.

A colorless oil, 38.1 mg, 79% yield.

TLC: R_f = 0.37 (PE/EA = 10:1).

^{1}H NMR (400 MHz, $CDCl_3$) δ 7.84 – 7.77 (m, 2H), 7.38 – 7.31 (m, 2H), 4.04 (dd, *J* = 9.7, 7.2 Hz, 1H), 3.64 (dd, *J* = 9.7, 7.1 Hz, 1H), 2.43 (s, 3H), 2.24 – 2.09 (m, 1H), 1.79 – 1.62 (m, 2H), 1.59 – 1.44 (m, 4H), 1.29 (s, 9H), 1.25 – 1.08 (m, 2H). Analytical data in agreement with the literature.[1]

Neopentyl *N*–(pivaloyl)-4-methylphenylsulfinimidate (3v')

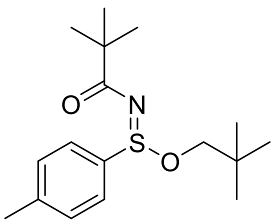

This compound was synthesized following *General procedure 2*, with 10.0 equiv of neopentyl alcohol as the alcohol substrate.

A colorless oil, 42.2 mg, 91% yield.

TLC: R_f = 0.86 (PE/EA = 3:1).

^{1}H NMR (400 MHz, $CDCl_3$) δ 7.92 – 7.74 (m, 2H), 7.43 – 7.28 (m, 2H), 3.81 (d, *J* = 9.4 Hz, 1H), 3.37 (d, *J* = 9.4 Hz, 1H), 2.43 (s, 3H), 1.28 (s, 9H), 0.86 (s, 9H). Analytical data in agreement with the literature.[1]

2-Iodoethyl *N*–(pivaloyl)-4-methylphenylsulfinimidate (3w')

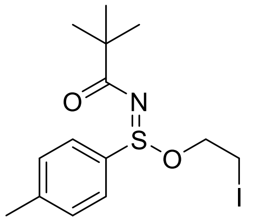

This compound was synthesized following *General procedure 2*, with 10.0 equiv of 2-iodoethanol as the alcohol substrate.

A colorless oil, 23.0 mg, 39% yield.

TLC: R_f = 0.74 (PE/EA = 3:1).

^{1}H NMR (400 MHz, $CDCl_3$) δ 7.92 – 7.83 (m, 2H), 7.40 – 7.32 (m, 2H), 4.44 – 4.34 (m, 1H), 4.14 – 4.04 (m, 1H), 3.33 – 3.19 (m, 2H), 2.45 (s, 3H), 1.29 (s, 9H). Analytical data in agreement with the literature.[1]

3-Hydroxypropyl *N*–(pivaloyl)-4-methylphenylsulfinimidate (3x')

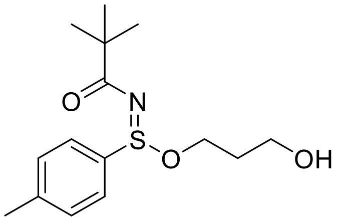

This compound was synthesized following *General procedure 2*.

When the reaction was conducted on a 0.10 mmol scale of sulfenamide using 2.5 equiv each of alcohol, NBS, and $NaHCO_3$, the product was obtained as a colorless oil (10.4 mg, 35% yield).

Under modified conditions with 2.0 equiv of alcohol, 1.2 equiv of NBS, and 1.5 equiv of $NaHCO_3$, the product was isolated as a colorless oil (19.0 mg, 64% yield).

TLC: R_f = 0.34 (PE/EA = 3:1).

^{1}H NMR (400 MHz, $CDCl_3$) δ 7.79 – 7.72 (m, 2H), 7.33 – 7.26 (m, 2H), 4.23 – 4.13 (m, 1H), 4.12 – 4.04 (m, 1H), 3.66 – 3.50 (m, 2H), 2.37 (s, 3H), 1.83 – 1.66 (m, 3H), 1.23 (s, 9H).

^{13}C NMR (101 MHz, $CDCl_3$) δ 194.1, 143.7, 133.8, 130.2, 127.7, 64.9, 58.3, 41.2, 32.6, 28.4, 21.7.

Analytical data in agreement with the literature.[1]

4-(3-(4-Cyano-3-(trifluoromethyl)phenyl)-5,5-dimethyl-2,4-dioxoimidazolidin-1-yl)butyl *N*–(pivaloyl)-4-methylphen-ylsulfinimidate (4)

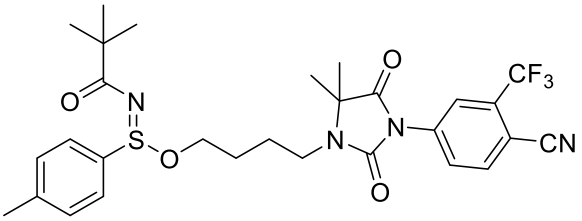

Following *General procedure 2*, the reaction was carried out on a 0.1 mmol scale with RU58841 (1.2 equiv) as the alcohol substrate.

A colorless oil, 31.2 mg, 53% yield.

TLC: R_f = 0.41 (PE/EA = 3:1).

^{1}H NMR (400 MHz, $CDCl_3$) δ 8.17 – 8.10 (m, 1H), 8.01 – 7.96 (m, 1H), 7.92 – 7.88 (m, 1H), 7.84 – 7.78 (m, 2H), 7.38 – 7.31 (m, 2H), 4.18 – 4.09 (m, 1H), 3.86 – 3.74 (m, 1H), 3.39 – 3.30 (m, 2H), 2.43 (s, 3H), 1.51 (d, J = 5.5 Hz, 6H), 1.27 (s, 9H).

^{19}F NMR (376 MHz, $CDCl_3$) δ -62.0. Analytical data in agreement with the literature.[1]

(1*R*,2*S*,5*R*)-2-Isopropyl-5-methylcyclohexyl *N*–(pivaloyl)-4-methylphenylsulfinimidate (5)

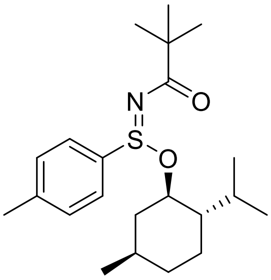

Following *General procedure 2*, the reaction was carried out on a 1.34 mmol scale with L-menthol (1.0 equiv) as the alcohol substrate in DCM (5.0 mL).

A colorless oil, 337.0 mg, 67% yield.

TLC: R_f = 0.61 (PE/EA = 10:1).

Major diastereomer (5-1)

^{1}H NMR (400 MHz, $CDCl_3$) δ 7.80 – 7.75 (m, 2H), 7.35 – 7.30 (m, 2H), 4.63 – 4.54 (m, 1H), 2.52 – 2.45 (m, 1H), 2.42 (s, 3H), 2.09 – 1.97 (m, 1H), 1.71 – 1.51 (m, 4H), 1.27 (s, 9H), 1.23 – 1.14 (m, 1H), 1.03 – 1.00 (m, 1H), 0.95 (d, *J* = 6.5 Hz, 3H), 0.91 – 0.83 (m, 1H), 0.81 (d, *J* = 7.1 Hz, 3H), 0.69 (d, *J* = 6.9 Hz, 3H).

^{13}C NMR (101 MHz, $CDCl_3$) δ 193.2, 142.9, 135.1, 130.0, 127.8, 82.5, 48.4, 43.0, 40.9, 34.2, 31.7, 25.5, 23.4, 22.3, 21.6, 21.0, 16.1.

HRMS (ESI+): m/z calcd for $C_{22}H_{35}NO_2SNa^+$ $[M+Na]^+$: 400.2281, found: 400.2281.

Minor diastereomer (5-2)

^{1}H NMR (400 MHz, $CDCl_3$) δ 7.86 – 7.77 (m, 2H), 7.37 – 7.30 (m, 2H), 4.57 – 4.47 (m, 1H), 2.43 (s, 3H), 2.09 – 1.98 (m, 2H), 1.70 – 1.60 (m, 4H), 1.57 – 1.44 (m, 1H), 1.27 (s, 9H), 1.13 – 1.02 (m, 2H), 0.92 – 0.81 (m, 9H).

^{13}C NMR (101 MHz, $CDCl_3$) δ 192.9, 143.2, 135.6, 130.0, 127.4, 84.0, 48.6, 42.9, 41.0, 34.0, 32.0, 28.3, 25.7, 23.1, 22.2, 21.7, 21.2, 15.9.

HRMS (ESI+): m/z calcd for $C_{22}H_{35}NO_2SNa^+$ $[M+Na]^+$: 400.2281, found: 400.2280.

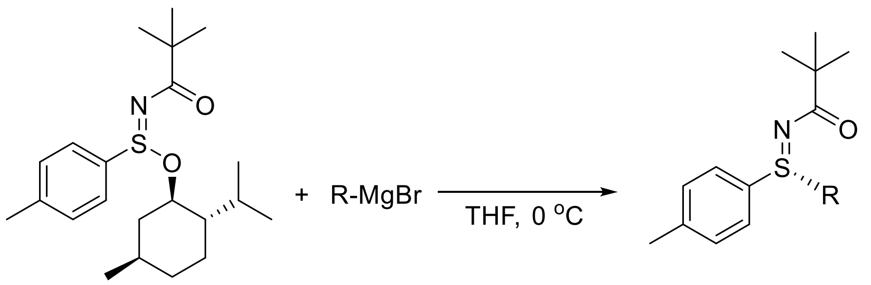

5 **6**

To a flame-dried round-bottomed flask under argon atmosphere were added sulfilimidate ester **5** (0.1 mmol, 1.0 equiv) and anhydrous THF (sufficient to achieve 0.02 M concentration). The mixture was cooled to 0 °C, and a solution of the corresponding R–MgBr (0.2 mmol, 2.0 equiv) was added dropwise under argon. The reaction was stirred at 0 °C overnight. Upon completion, the reaction was quenched with saturated aqueous NH_4Cl, extracted with EtOAc, dried over Na_2SO_4, filtered, and concentrated under reduced pressure. The crude product was purified by column chromatography to afford compound **6**.

N-(Methyl(*p*-tolyl)-λ^4-sulfanylidene)pivalamide (6a)

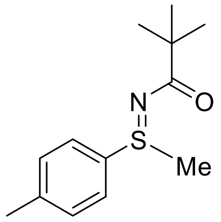

A colorless oil, 20.9 mg, 88% yield.
TLC: R_f = 0.41 (DCM/MeOH = 20:1).
^{1}H NMR (400 MHz, $CDCl_3$) δ 7.58 – 7.52 (m, 2H), 7.27 – 7.22 (m, 2H), 2.68 (s, 3H), 2.33 (s, 3H), 1.16 (s, 9H). Analytical data in agreement with the literature.[2]

Chiral HPLC chromatogram of *rac*-**6a**: HPLC using a Chiralpak IA column (*n*-hexane/iPrOH 90:10, flow rate = 1.0 mL/min, λ = 254 nm, 25 °C):

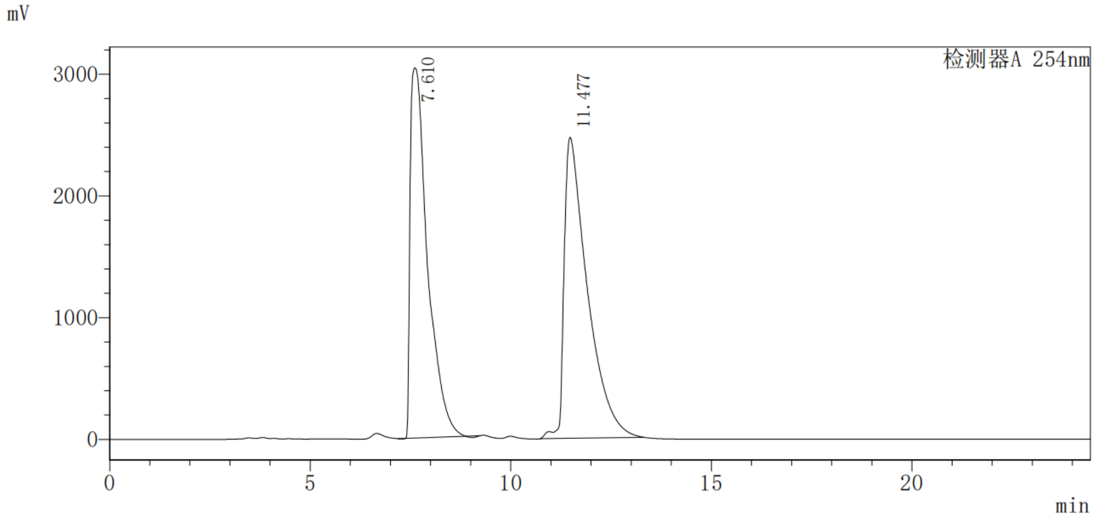

A 254nm

Peak No.	Ret. time	Area	Hight	Area%			
1	7.610	91199794	3042351	47.757			
2	11.477	99765367	2472662	52.243			
total		190965161	5515013				

Figure S1: HPLC chromatogram of racemic compound 6a

Chiral HPLC chromatogram of **6a-1** derived from **major diastereomer 5-1**: HPLC using a Chiralpak IA column (*n*-hexane/iPrOH 90:10, flow rate = 1.0 mL/min, λ = 254 nm, 25 °C).

The enantiomeric excess (ee) of compound **6a-1** is 92%.

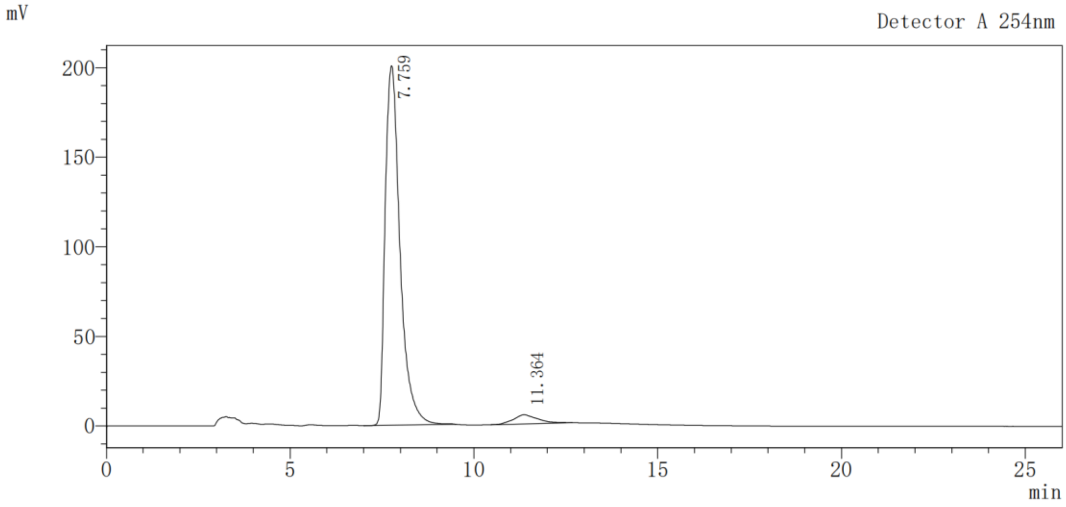

Detector A 254nm

prak no.	Ret. time	area	hight	area%			
1	7.759	5614530	200661	96.030			
2	11.364	232092	5055	3.970			
total		5846622	205716				

Figure S2: HPLC chromatogram of compound 6a-1

Chiral HPLC chromatogram of **6a-2** derived from **minor diastereomer 5-2**: HPLC using a Chiralpak IA column (*n*-hexane/iPrOH 90:10, flow rate = 1.0 mL/min, λ = 254 nm, 25 °C).

The enantiomeric excess (ee) of compound **6a-2** is 66%.

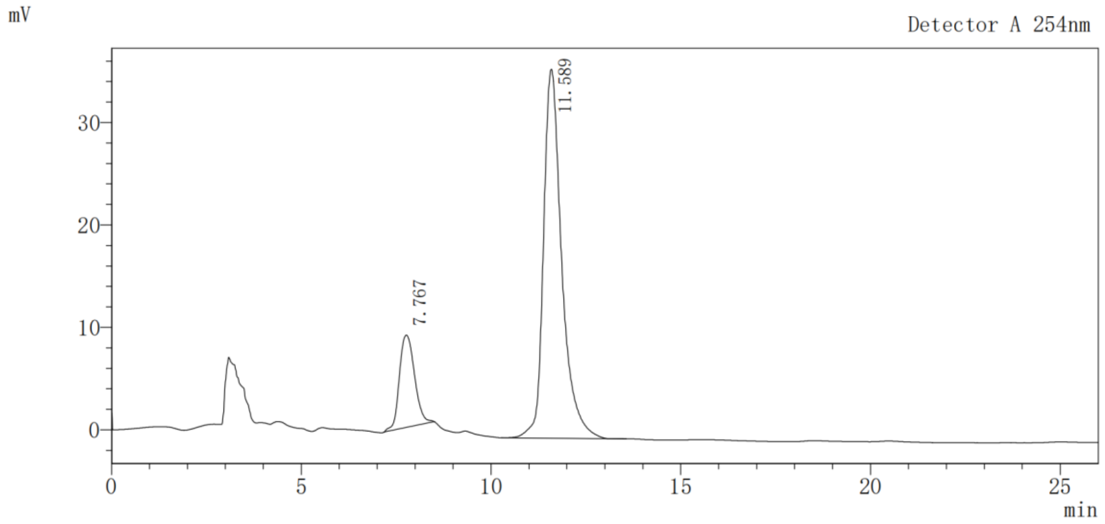

Detector A 254nm

peak No.	ret. time	area	hight	area%			
1	7.767	250173	9006	17.212			
2	11.589	1203321	36022	82.788			
total		1453494	45028				

Figure S3: HPLC chromatogram of compound 6a-2

(*R*)-*N*-(Phenyl(*p*-tolyl)-λ^4-sulfanylidene)pivalamide (6b)

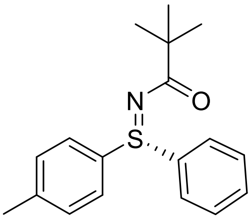

A colorless oil, 25.5 mg, 85% yield.

TLC: R_f = 0.75 (DCM/MeOH = 20:1).

^{1}H NMR (400 MHz, $CDCl_3$) δ 7.67 – 7.60 (m, 2H), 7.58 – 7.50 (m, 2H), 7.42 – 7.31 (m, 3H), 7.23 – 7.11 (m, 2H), 2.28 (s, 3H), 1.21 (s, 9H). Analytical data in agreement with the literature.[3]

Chiral HPLC chromatogram of *rac*-**6b**: HPLC using a Chiralpak IA column (*n*-hexane/iPrOH 90:10, flow rate = 1.0 mL/min, λ = 254 nm, 25 °C).

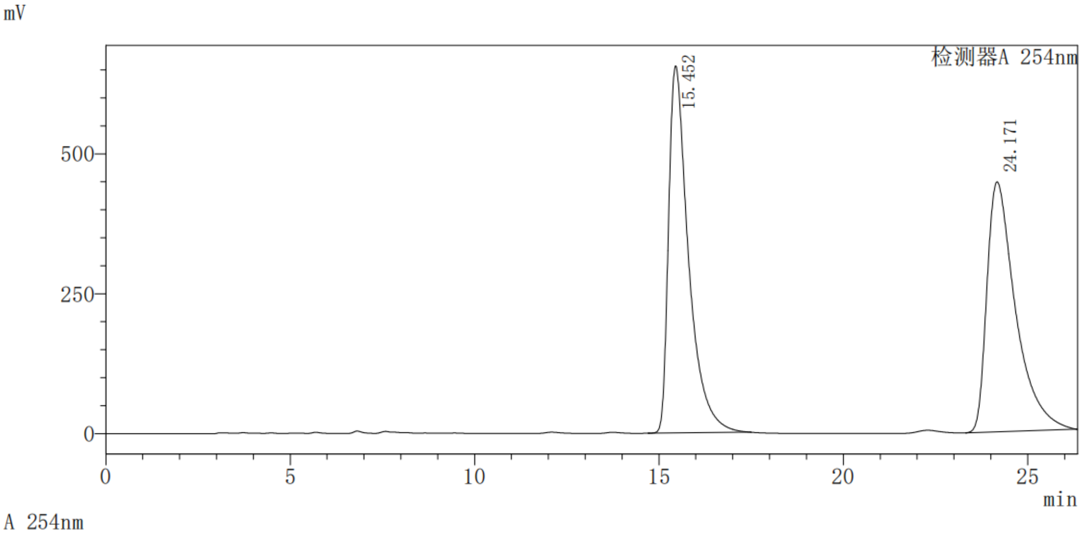

A 254nm

Peak No.	Ret. time	Area	Hight	Area%			
1	15.452	24654545	655890	50.529			
2	24.171	24138561	446719	49.471			
total		48793105	1102608				

Figure S4: HPLC chromatogram of racemic compound 6b

Chiral HPLC chromatogram of **6b-1** derived from **Major diastereomer 5-1**: HPLC using a Chiralpak IA column (*n*-hexane/iPrOH 90:10, flow rate = 1.0 mL/min, λ = 254 nm, 25 °C).

The enantiomeric excess (ee) of compound **6b-1** is 93%.

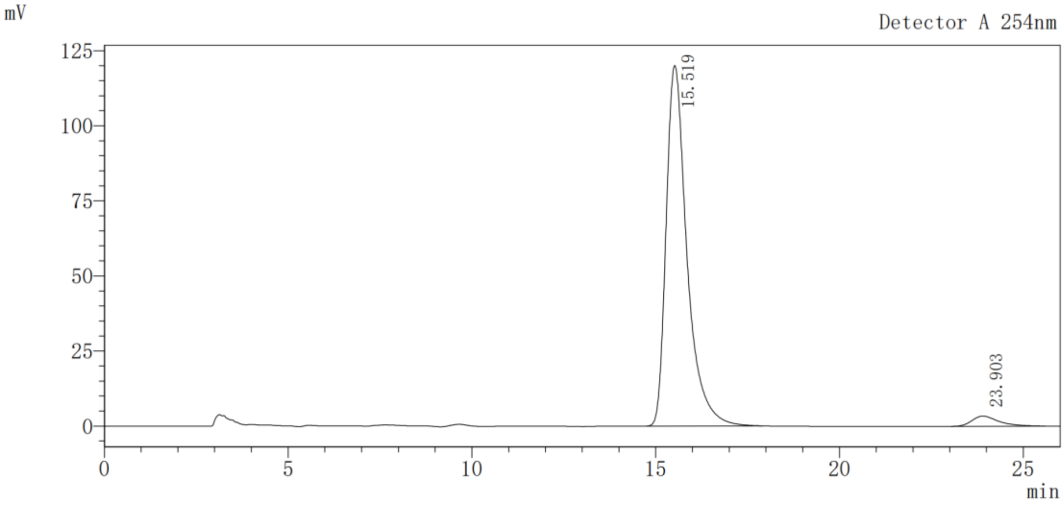

Detector A 254nm

peak no	Ret. time	area	hight	area%			
1	15.519	4727314	120072	96.519			
2	23.903	170477	3390	3.481			
total		4897791	123463				

Figure S5: HPLC chromatogram of compound 6b-1

Chiral HPLC chromatogram of **6b-2** derived from **Minor diastereomer 5-2**: HPLC using a Chiralpak IA column (*n*-hexane/iPrOH 90:10, flow rate = 1.0 mL/min, λ = 254 nm, 25 °C).

The enantiomeric excess (ee) of compound **6b-2** is 65%.

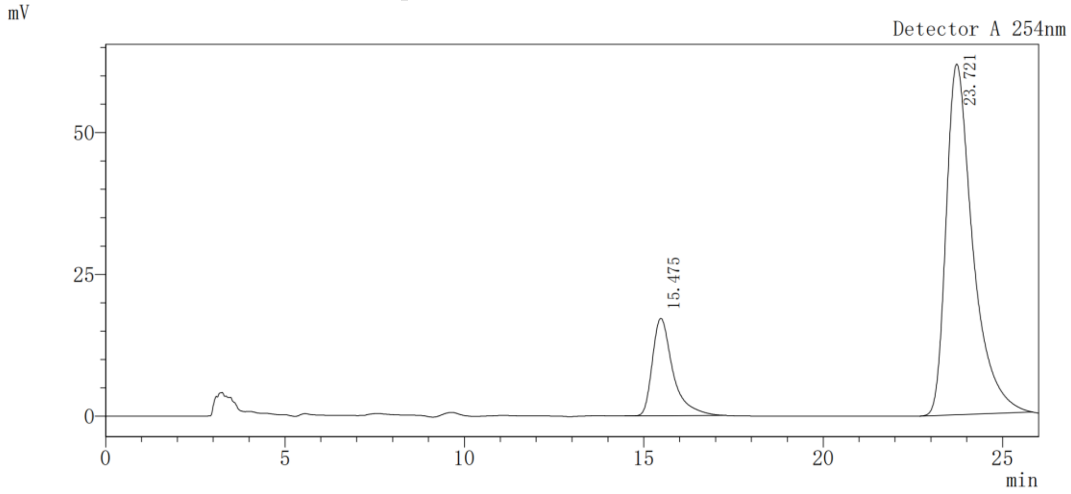

Detector A 254nm

peak NO.	Ret. time	Area	Hight	area%			
1	15.475	685218	17190	17.643			
2	23.721	3198556	61810	82.357			
total		3883774	79001				

Figure S6: HPLC chromatogram of compound 6b-2

(F) References

1. X. Lu, G. Huang, J. Ye, M. A. Bashir, J. Su, K. Yang, F. Liang, X. Xu, *Org. Lett.* **2023**, *25*, 2151.
2. G. Huang, X. Lu, K. Yang, X. Xu, *Org. Lett.* **2023**, *25*, 3173.
3. G. Huang, X. Lu, F. Liang, *Org. Lett.* **2023**, *25*, 3179.

(G) Copies of NMR spectra (^{1}H, ^{13}C, ^{19}F)

^{1}H NMR spectrum for compound **3a** (in CDCl3, 400 MHz)

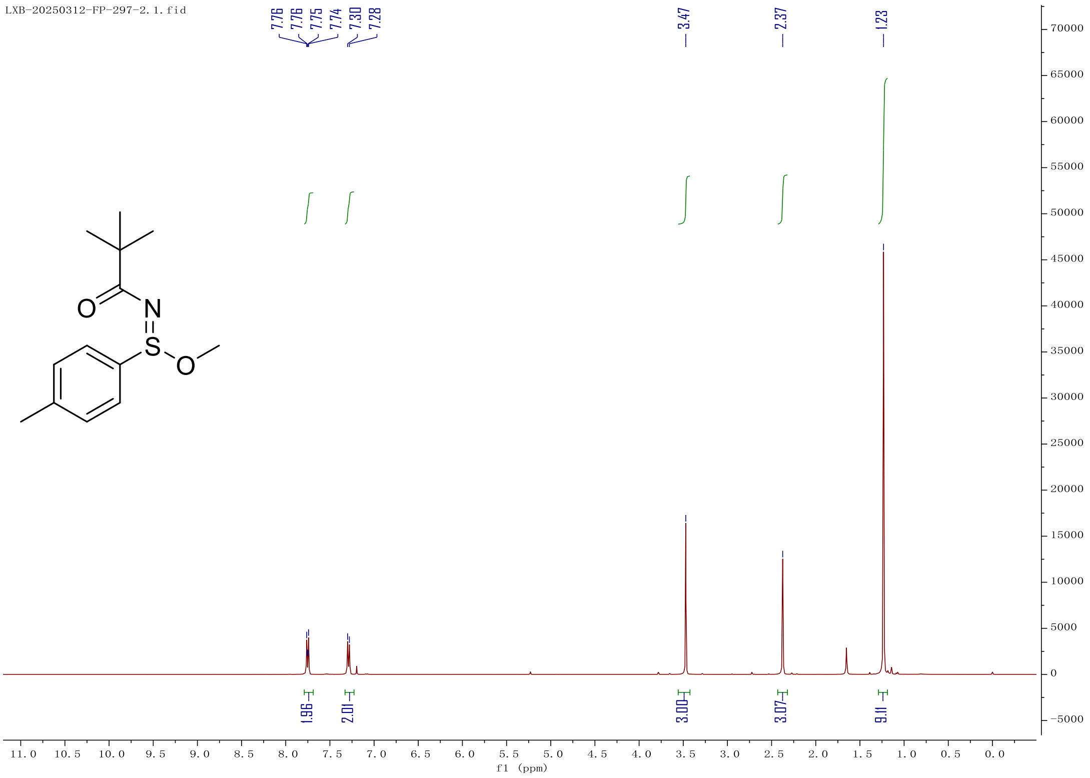

^{13}C NMR spectrum for compound **3a** (in CDCl3, 101 MHz)

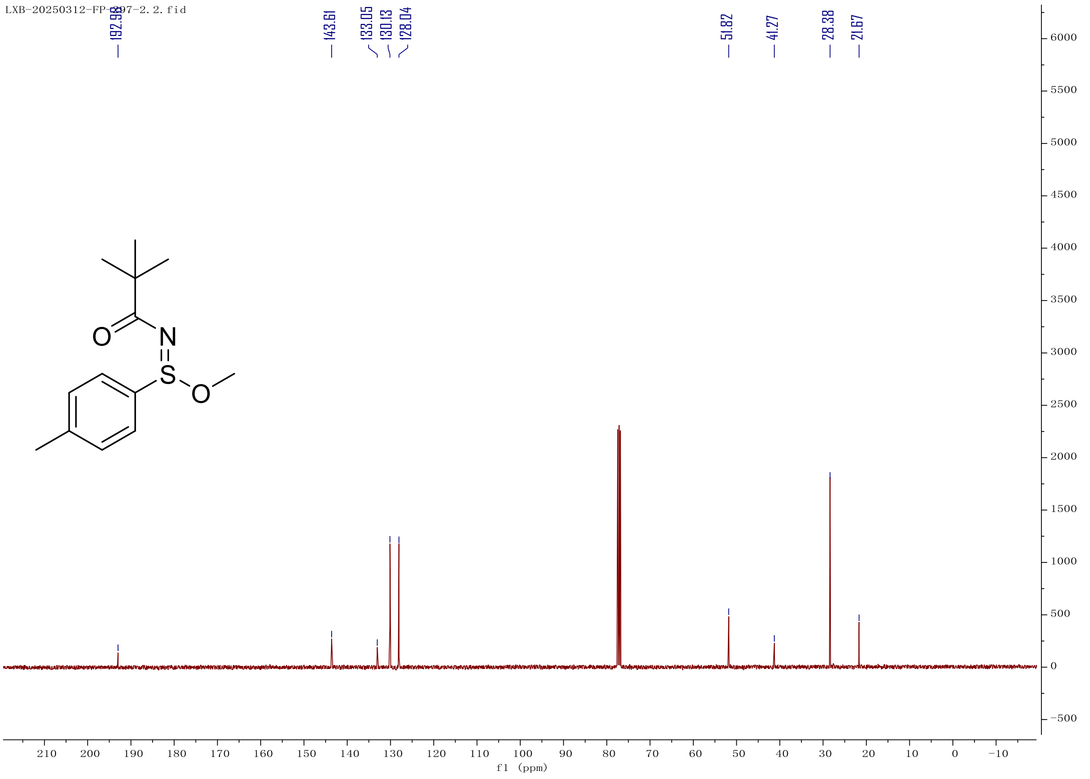

^{1}H NMR spectrum for compound **3b** (in CDCl3, 400 MHz)

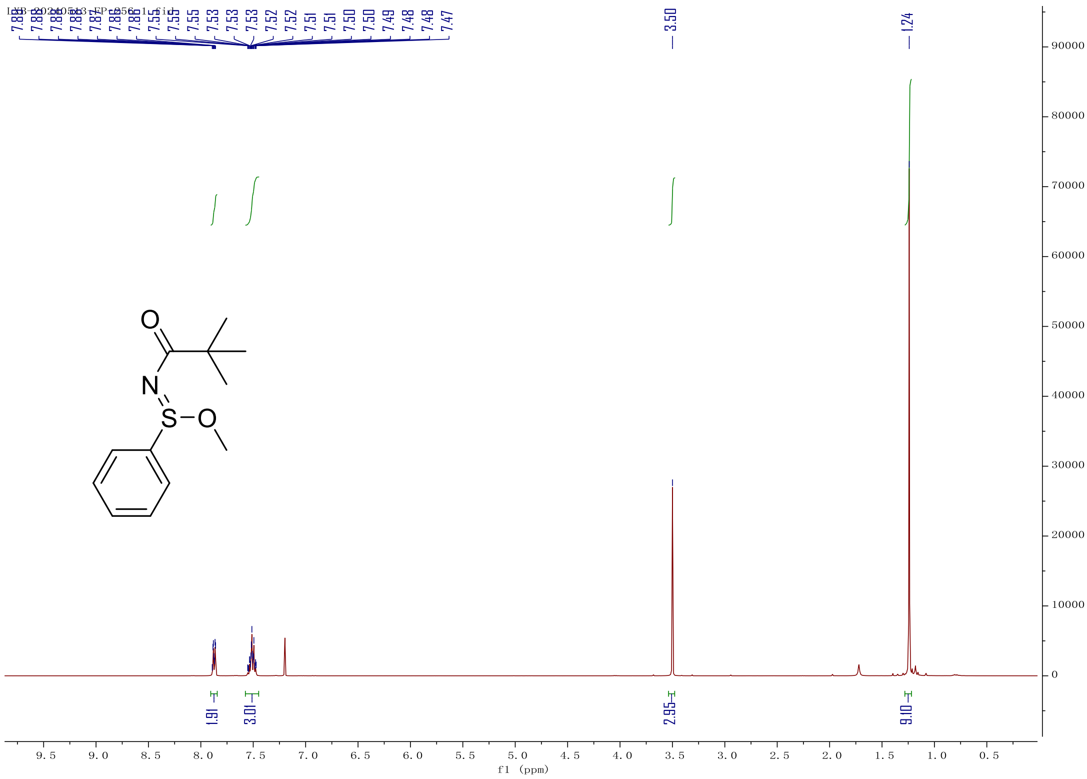

^{1}H NMR spectrum for compound **3c** (in $CDCl_3$, 400 MHz)

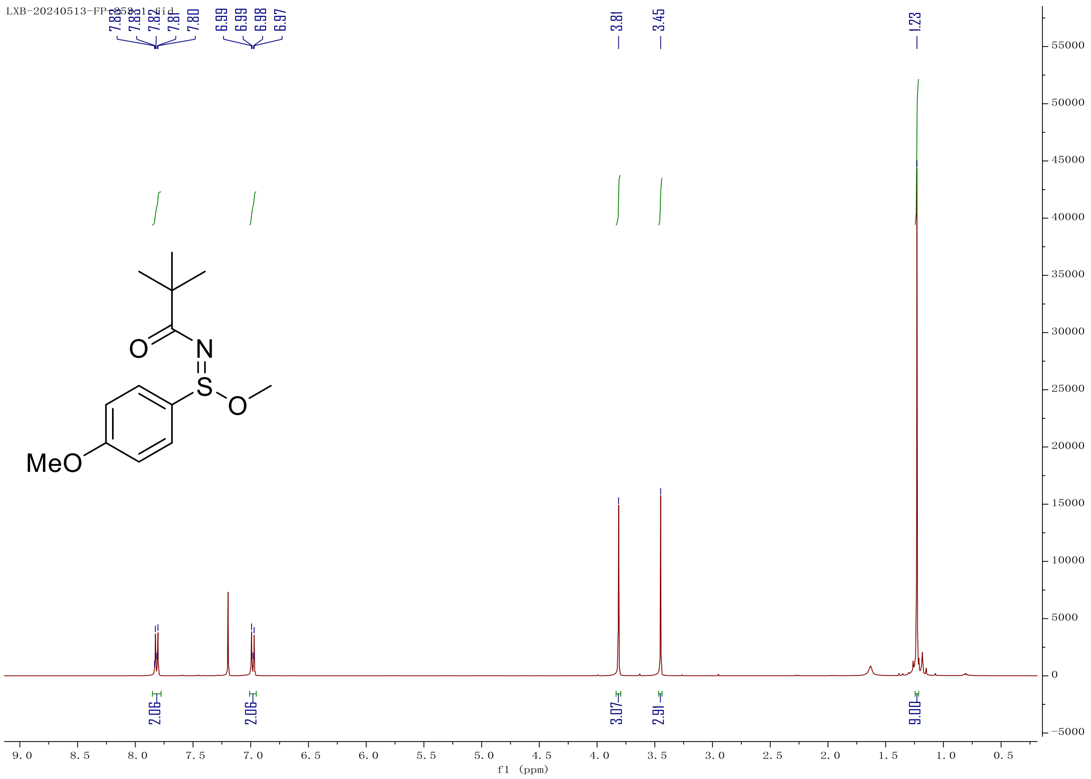

^{13}C NMR spectrum for compound **3c** (in $CDCl_3$, 101 MHz)

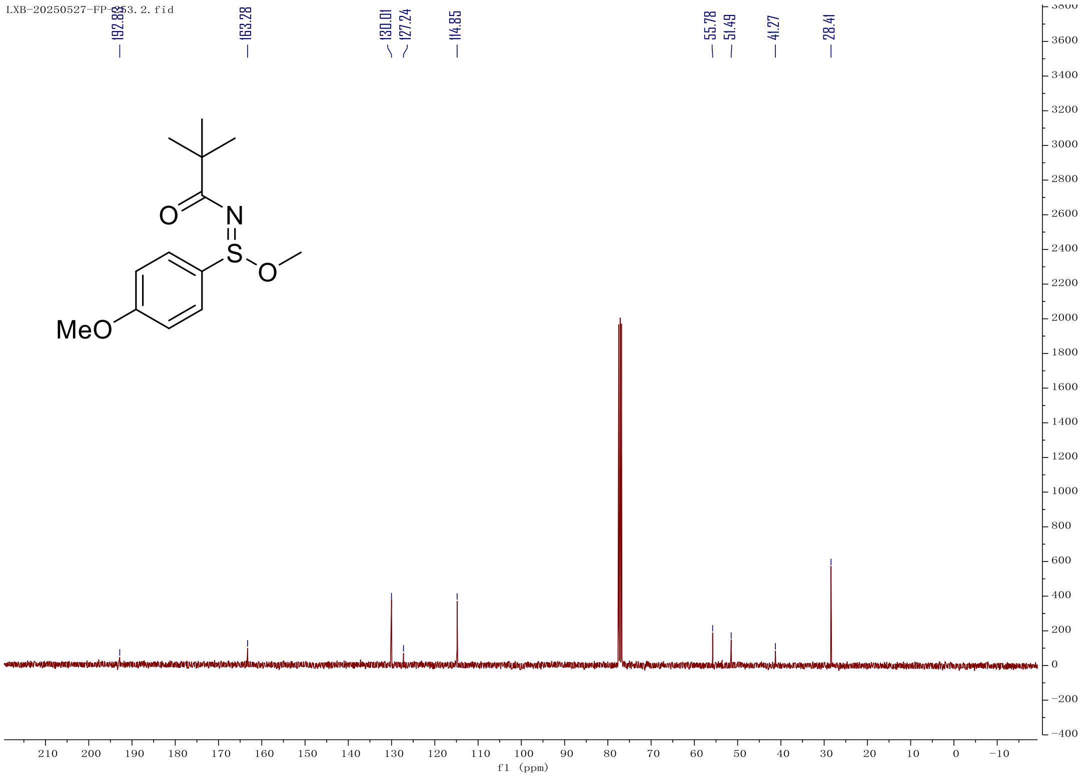

^{1}H NMR spectrum for compound **3d** (in CDCl3, 400 MHz)

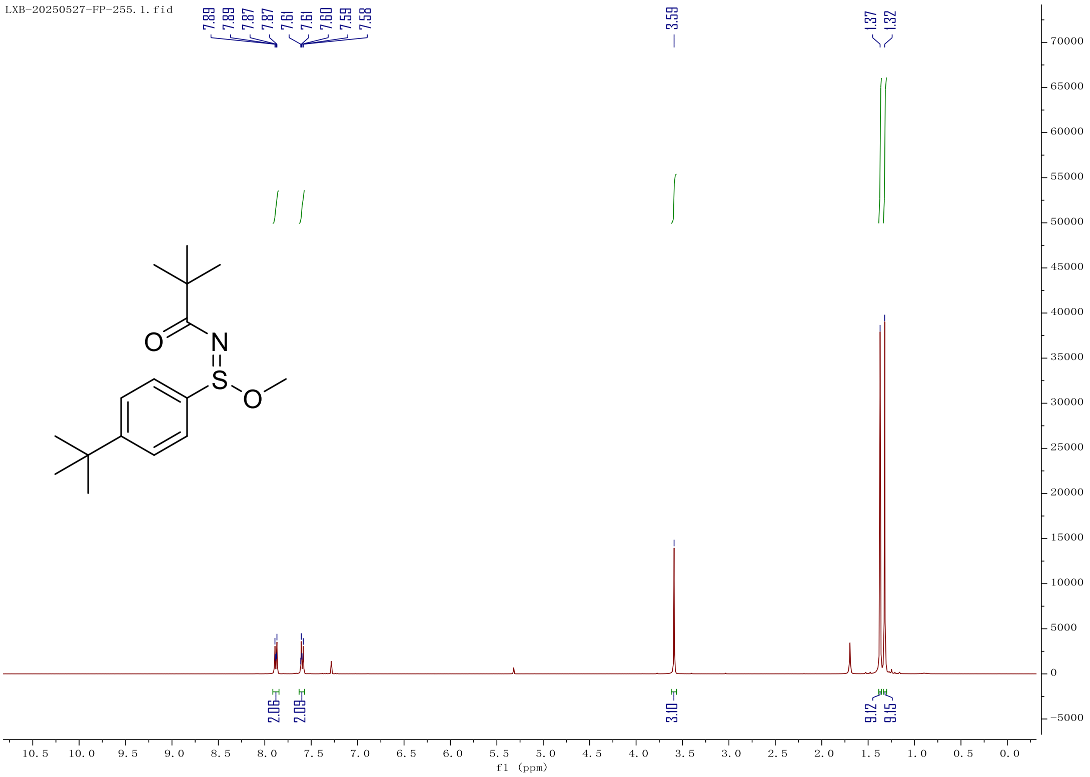

^{13}C NMR spectrum for compound **3d** (in CDCl3, 101 MHz)

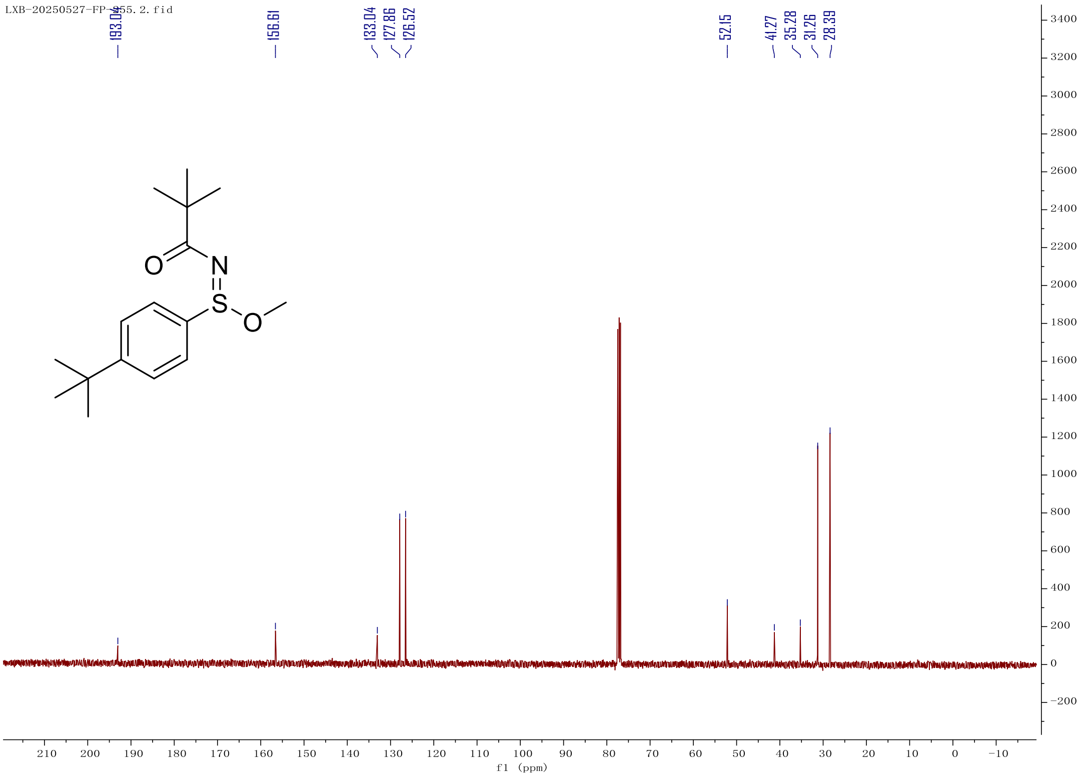

^{1}H NMR spectrum for compound **3e** (in $CDCl_3$, 400 MHz)

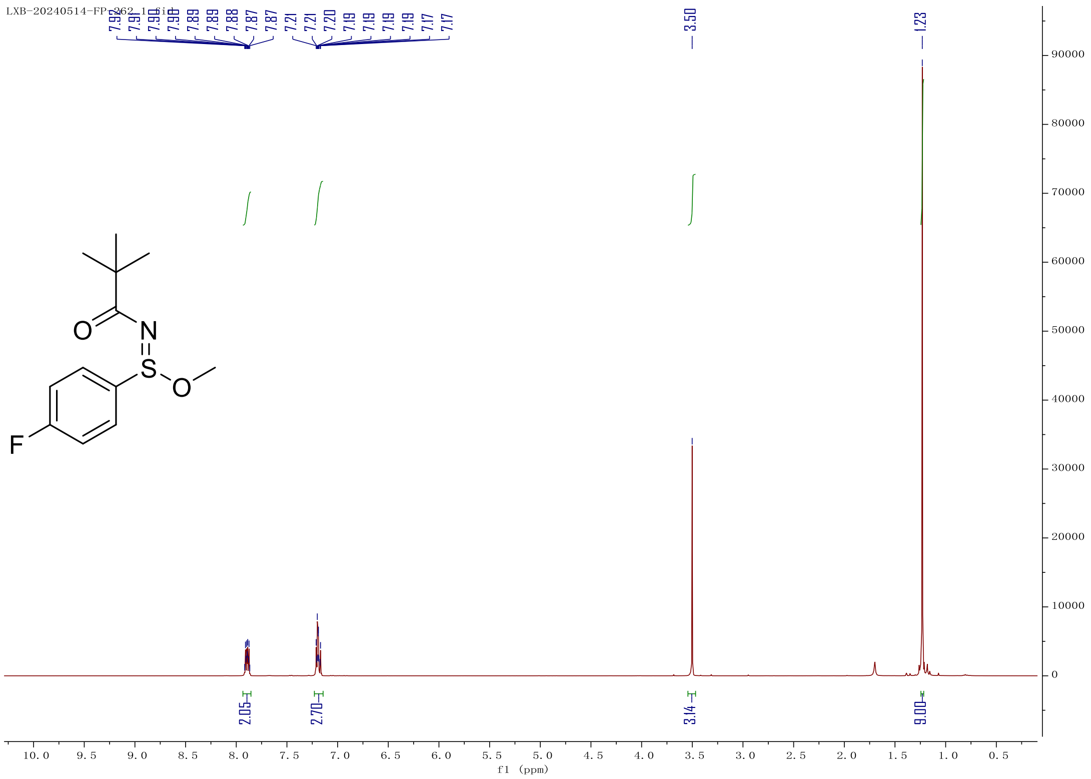

^{1}H NMR spectrum for compound **3f** (in $CDCl_3$, 400 MHz)

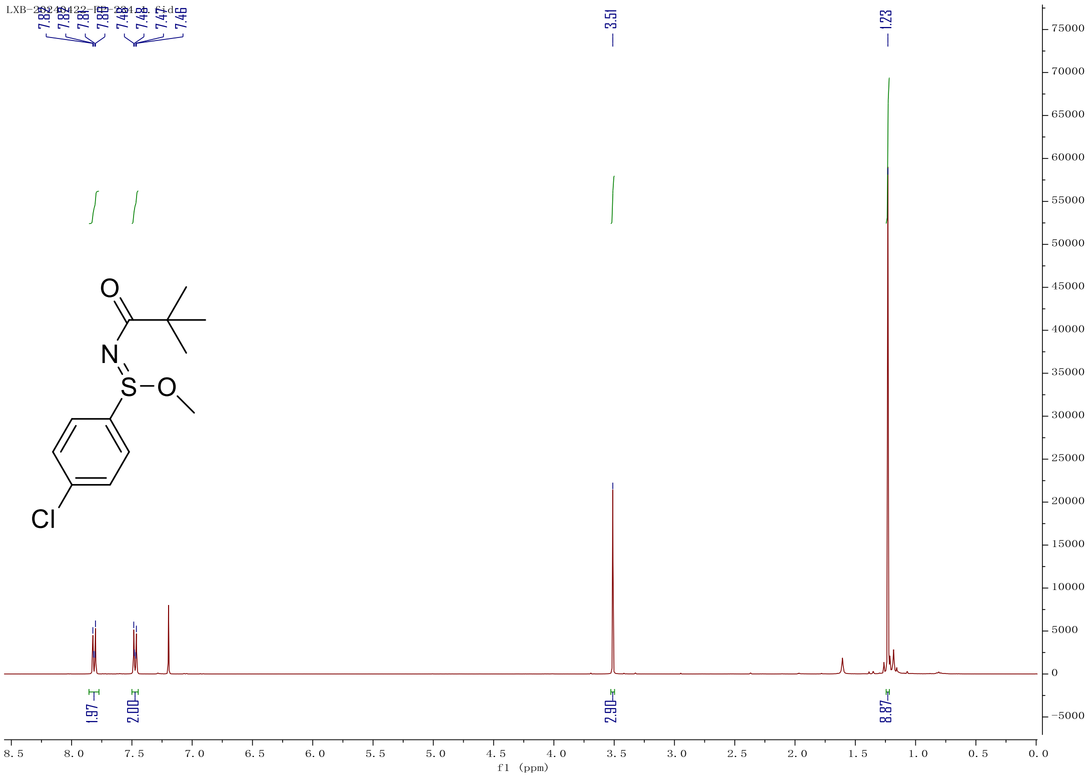

^{1}H NMR spectrum for compound **3g** (in CDCl3, 400 MHz)

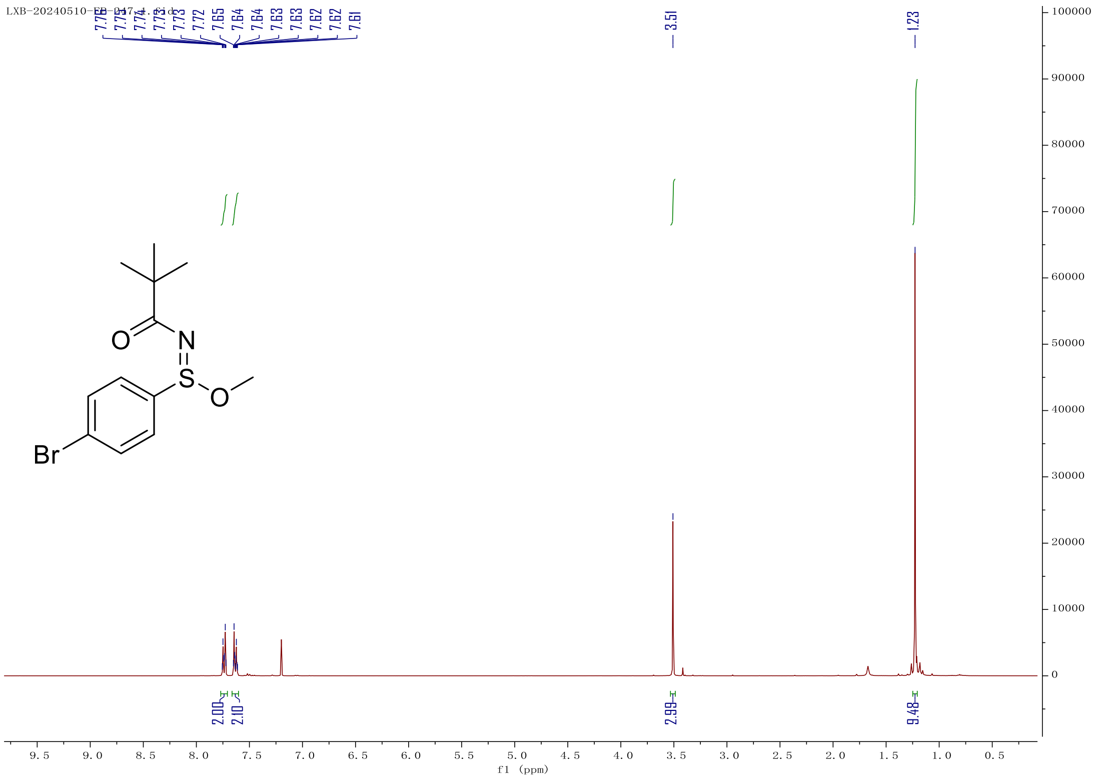

^{1}H NMR spectrum for compound **3h** (in CDCl3, 400 MHz)

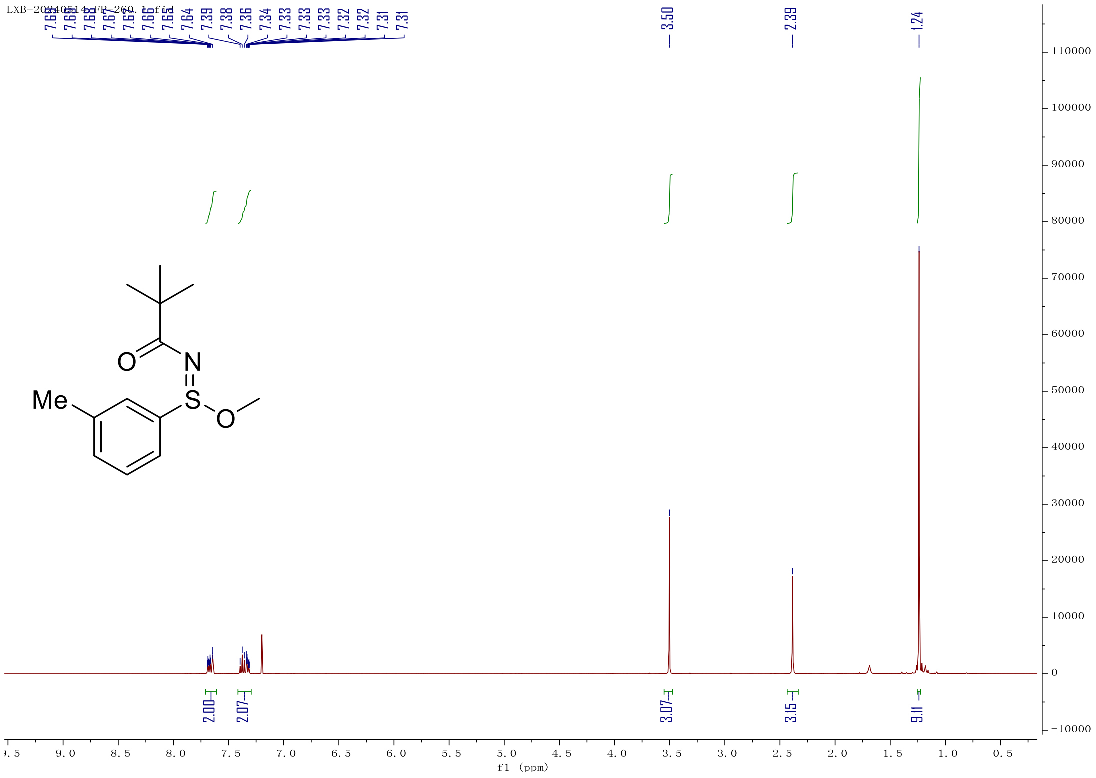

^{1}H NMR spectrum for compound **3i** (in CDCl3, 400 MHz)

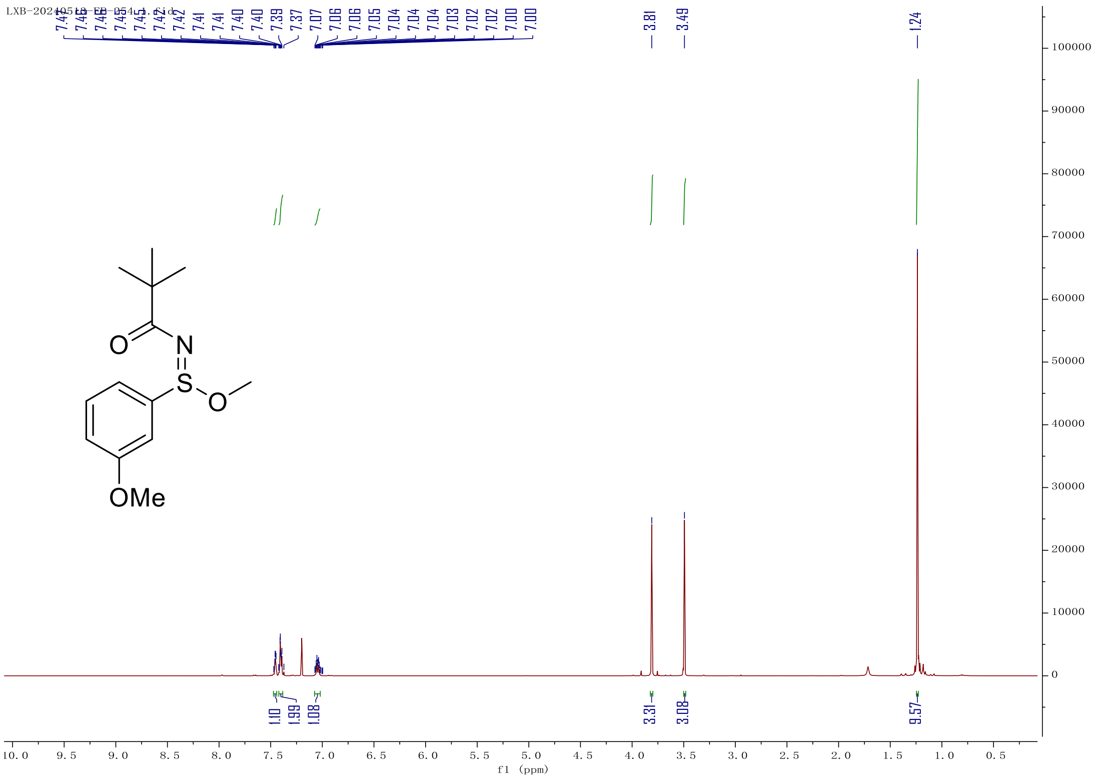

^{13}C NMR spectrum for compound **3i** (in CDCl3, 101 MHz)

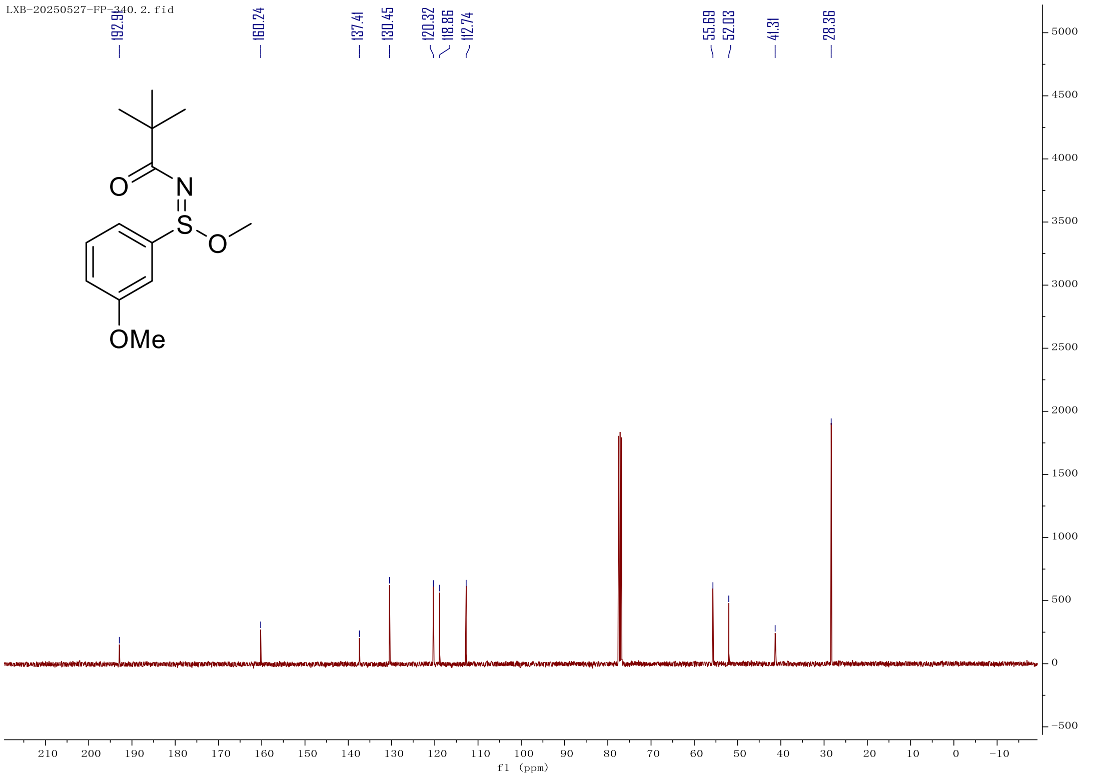

^{1}H NMR spectrum for compound **3j** (in $CDCl_3$, 400 MHz)

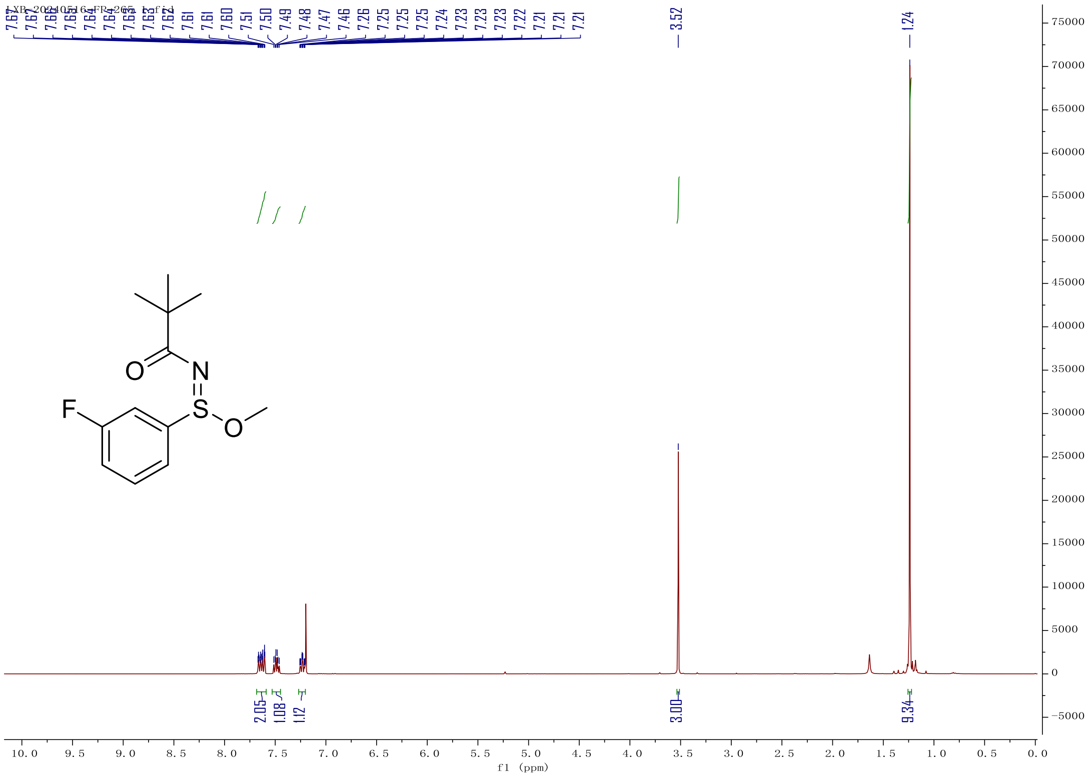

^{1}H NMR spectrum for compound **3k** (in $CDCl_3$, 400 MHz)

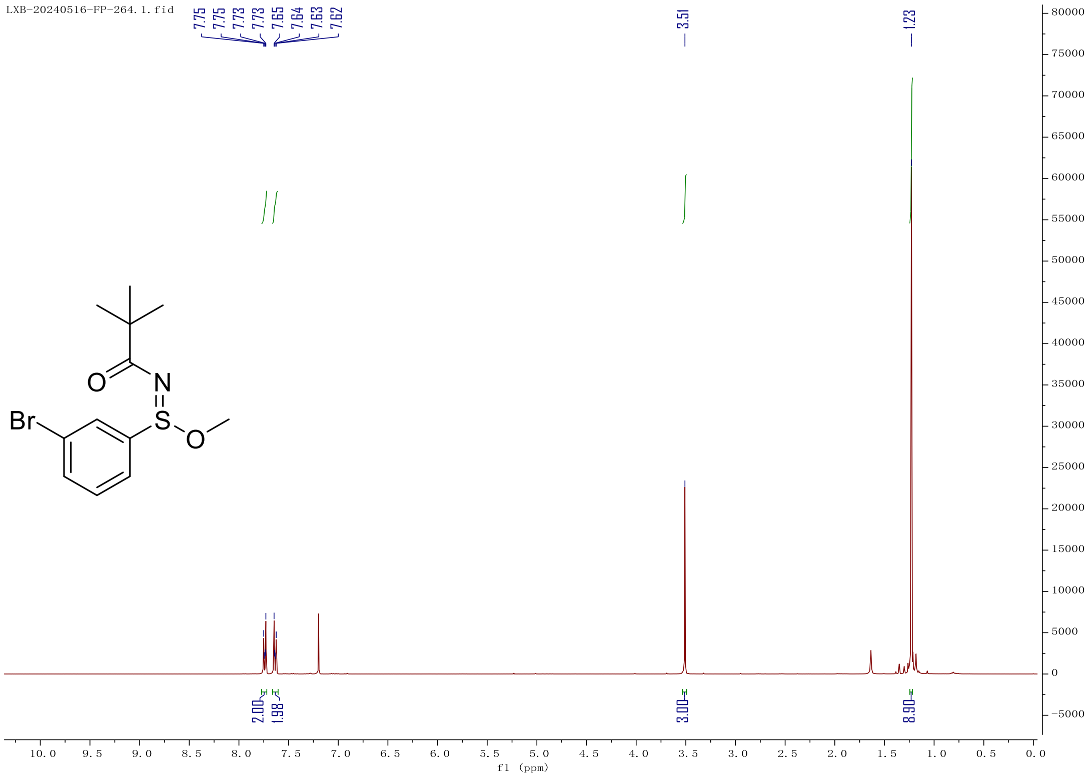

^{1}H NMR spectrum for compound **3l** (in $CDCl_3$, 400 MHz)

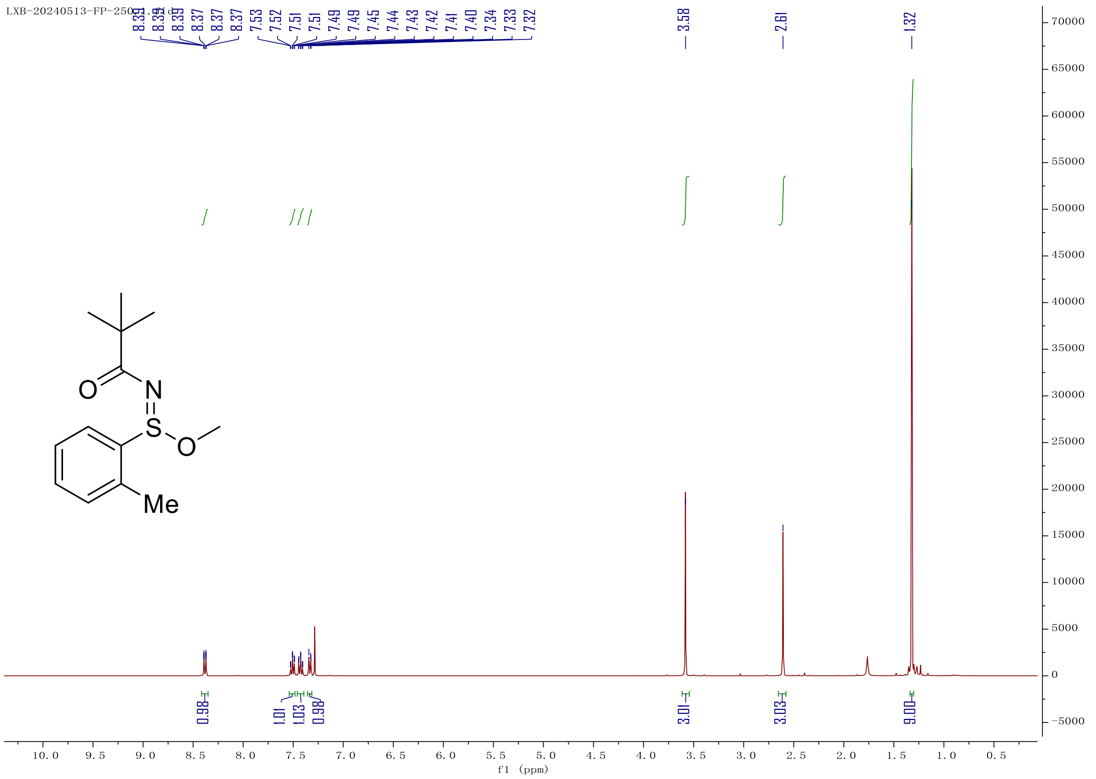

^{1}H NMR spectrum for compound **3m** (in $CDCl_3$, 400 MHz)

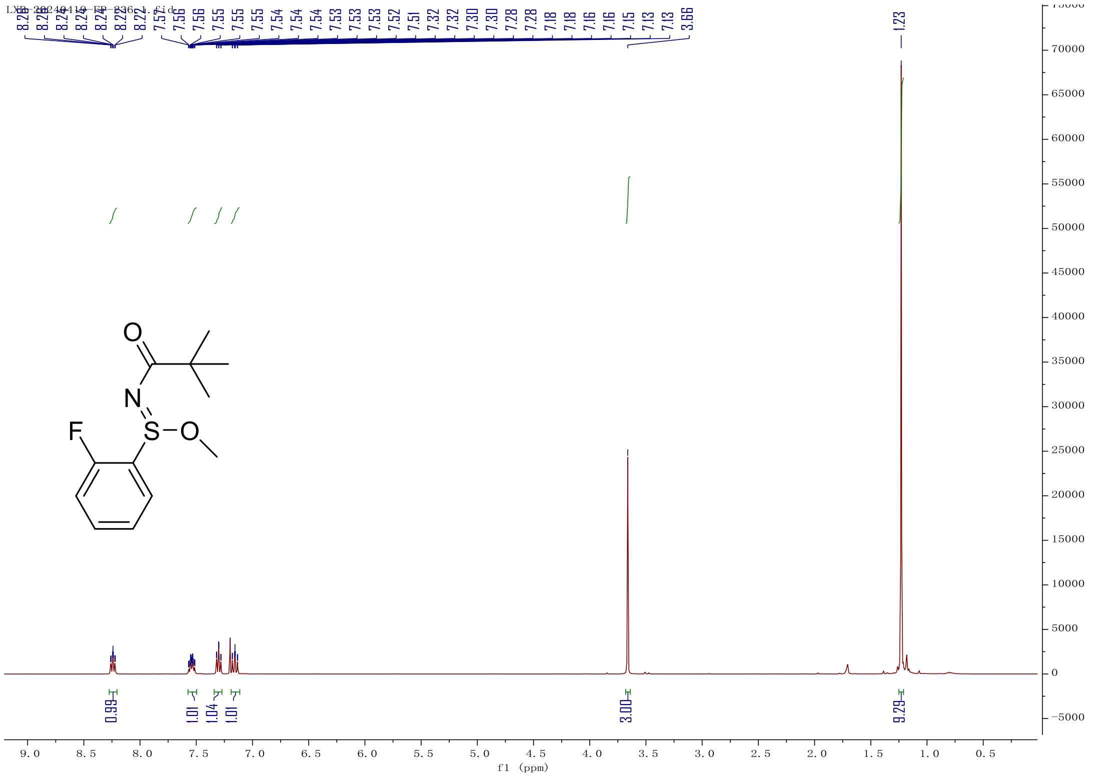

^{1}H NMR spectrum for compound **3n** (in CDCl3, 400 MHz)

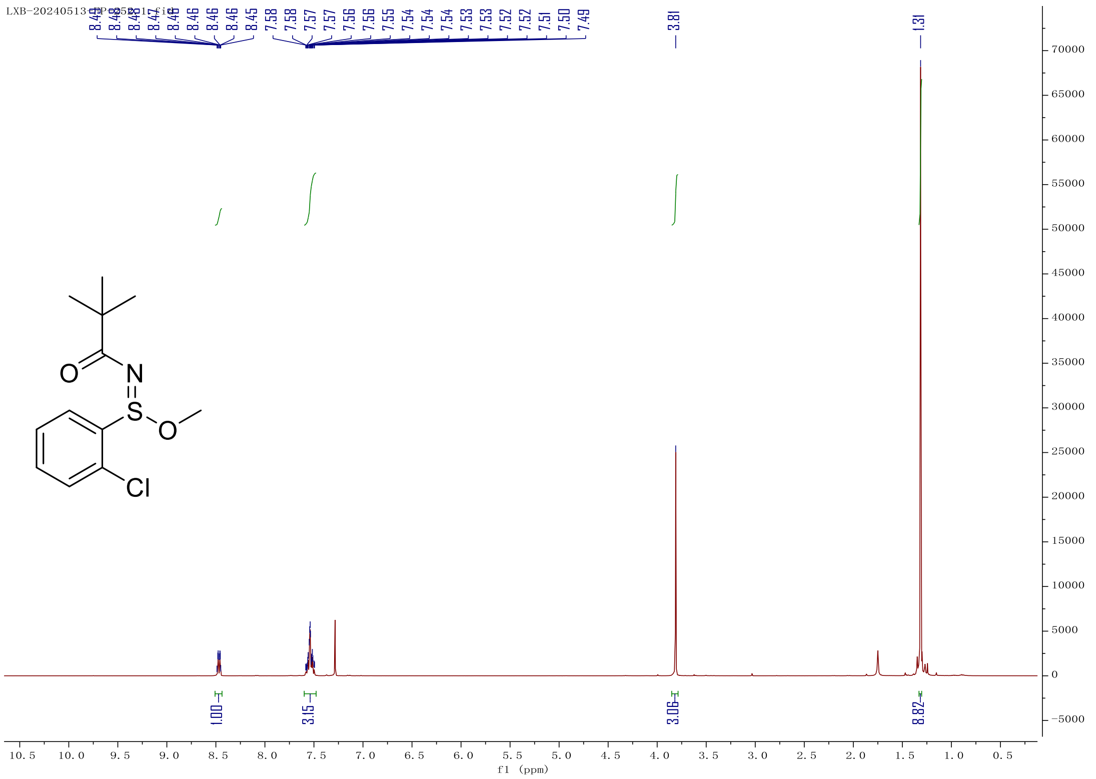

^{1}H NMR spectrum for compound **3o** (in CDCl3, 400 MHz)

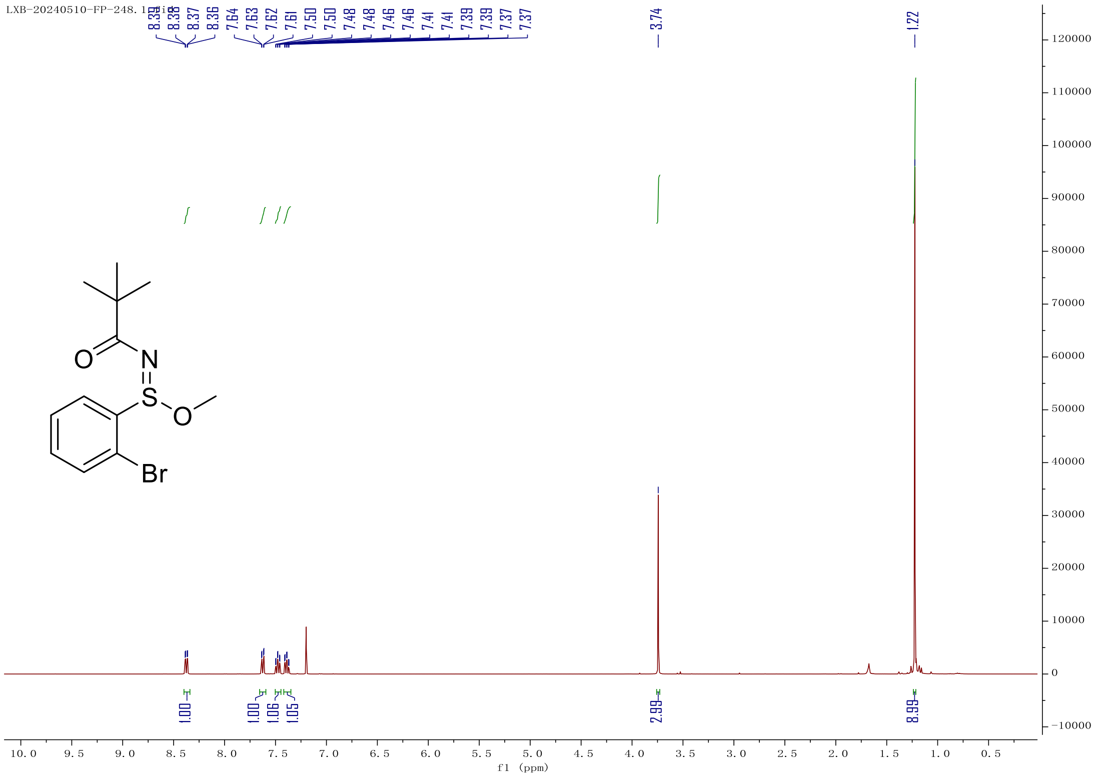

^{1}H NMR spectrum for compound **3p** (in $CDCl_3$, 400 MHz)

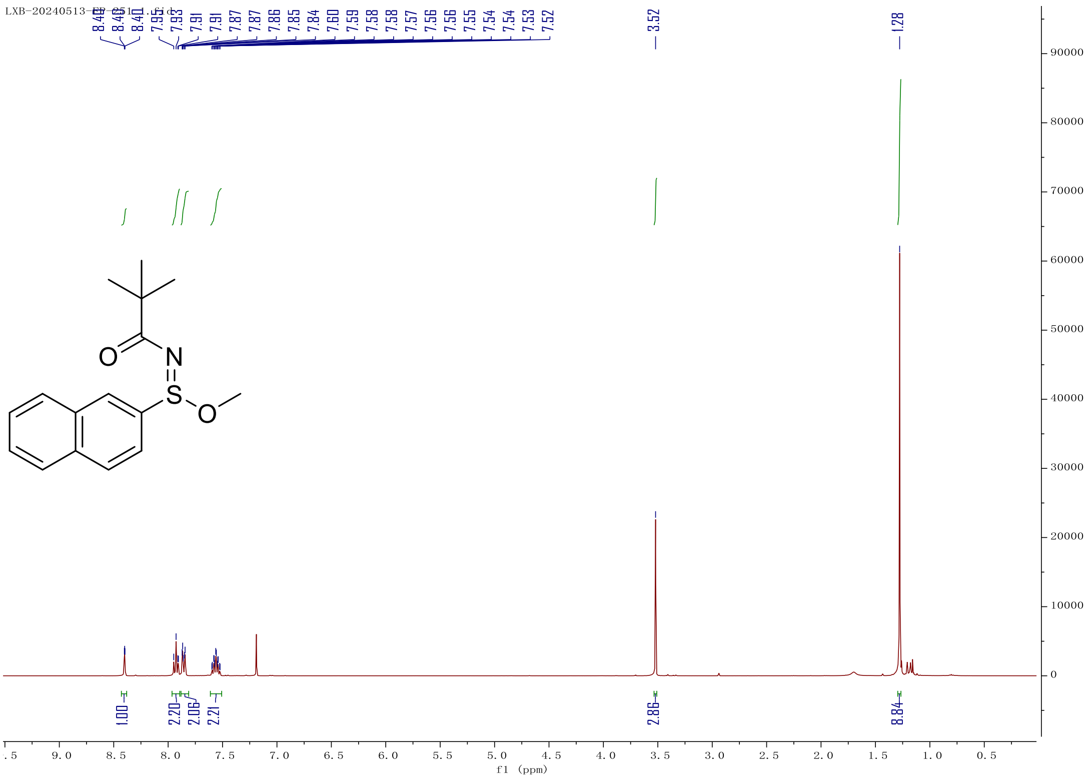

^{13}C NMR spectrum for compound **3p** (in $CDCl_3$, 101 MHz)

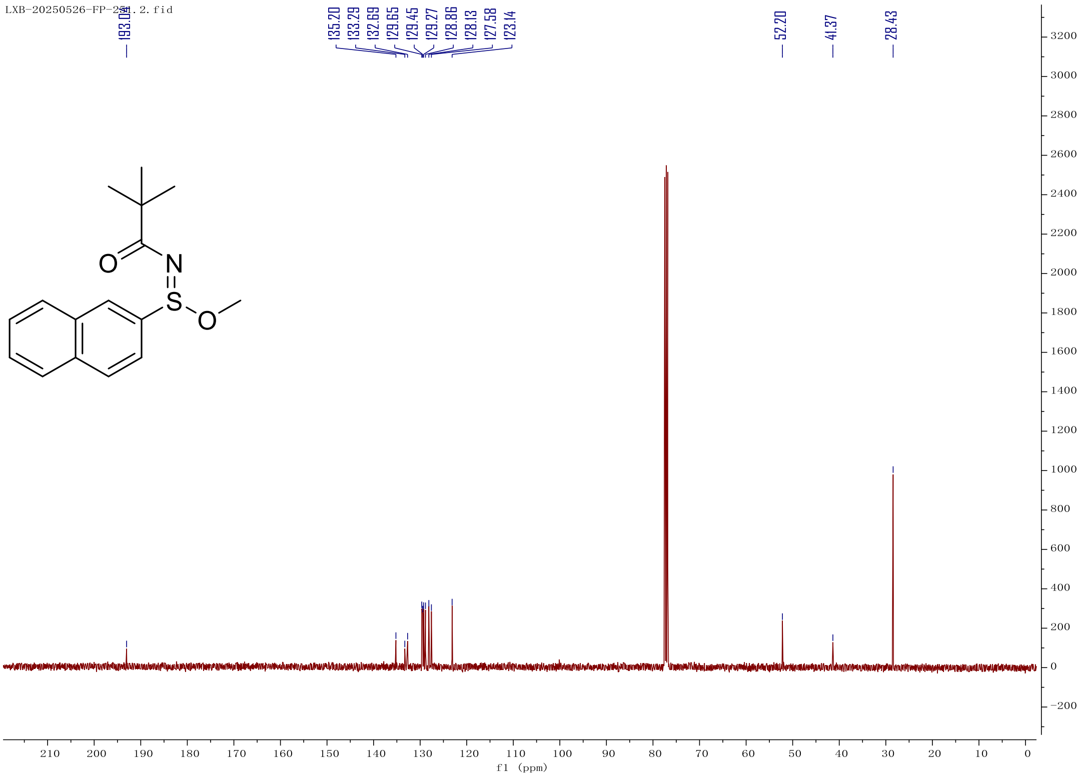

^{1}H NMR spectrum for compound **3q** (in $CDCl_3$, 400 MHz)

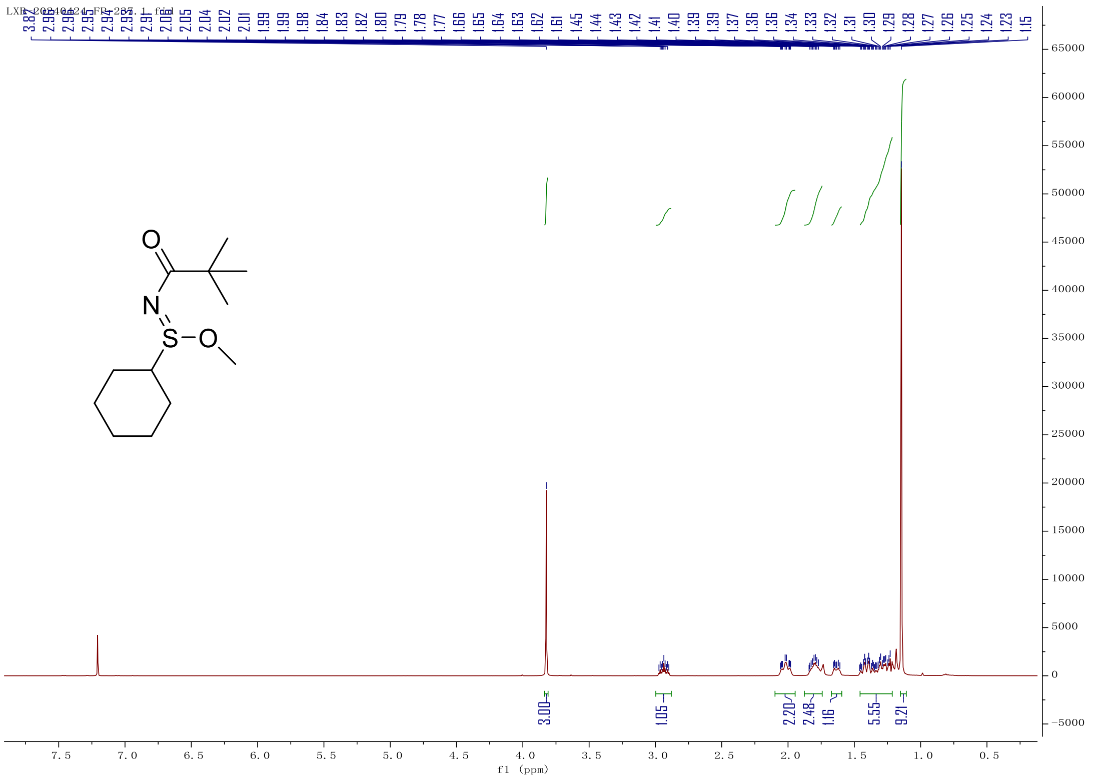

^{1}H NMR spectrum for compound **3r** (in $CDCl_3$, 400 MHz)

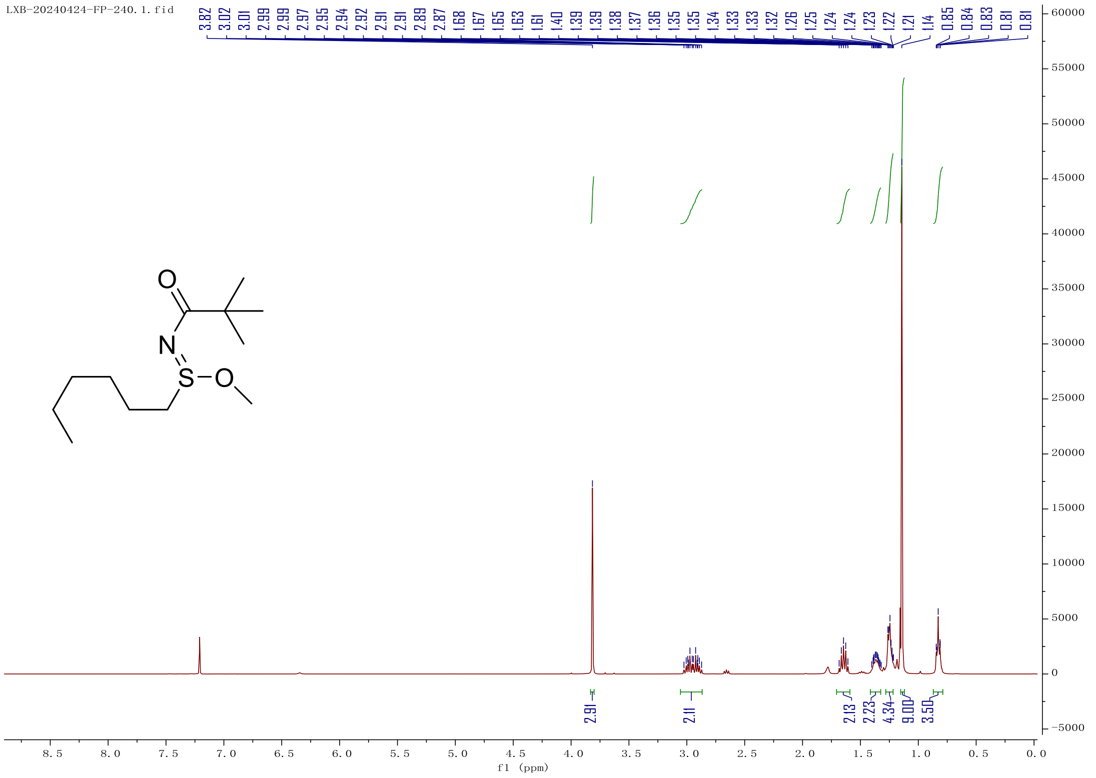

^{1}H NMR spectrum for compound **3s** (in $CDCl_3$, 400 MHz)

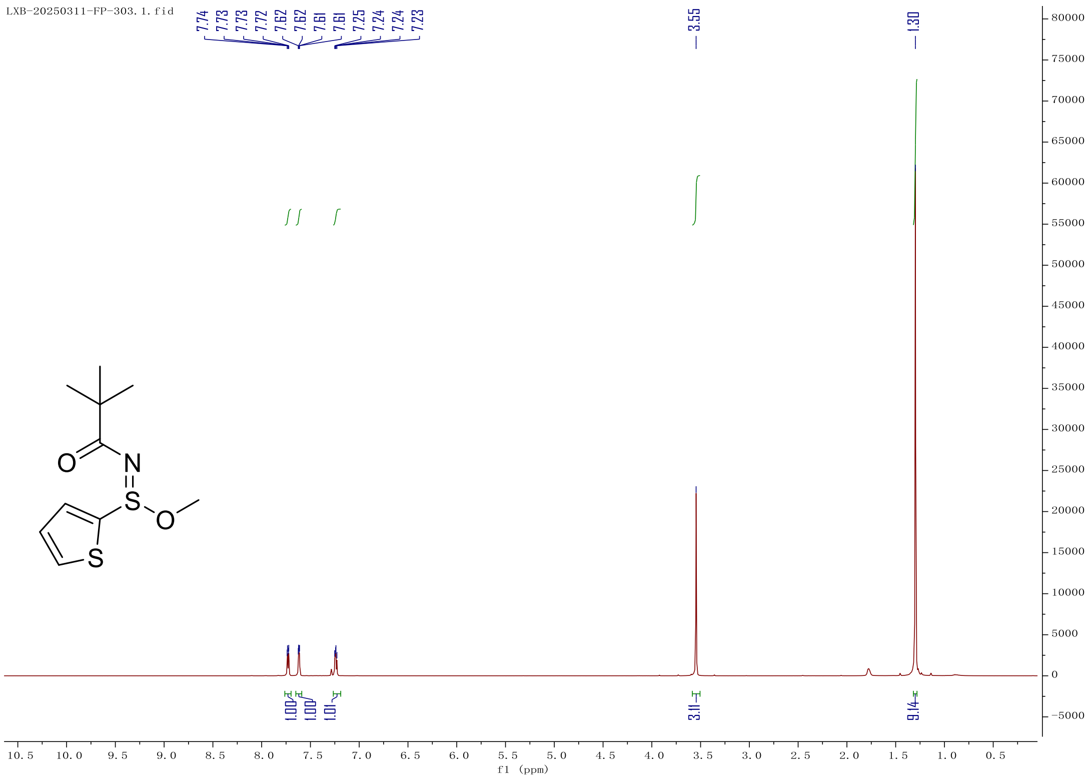

^{1}H NMR spectrum for compound **3t** (in $CDCl_3$, 400 MHz)

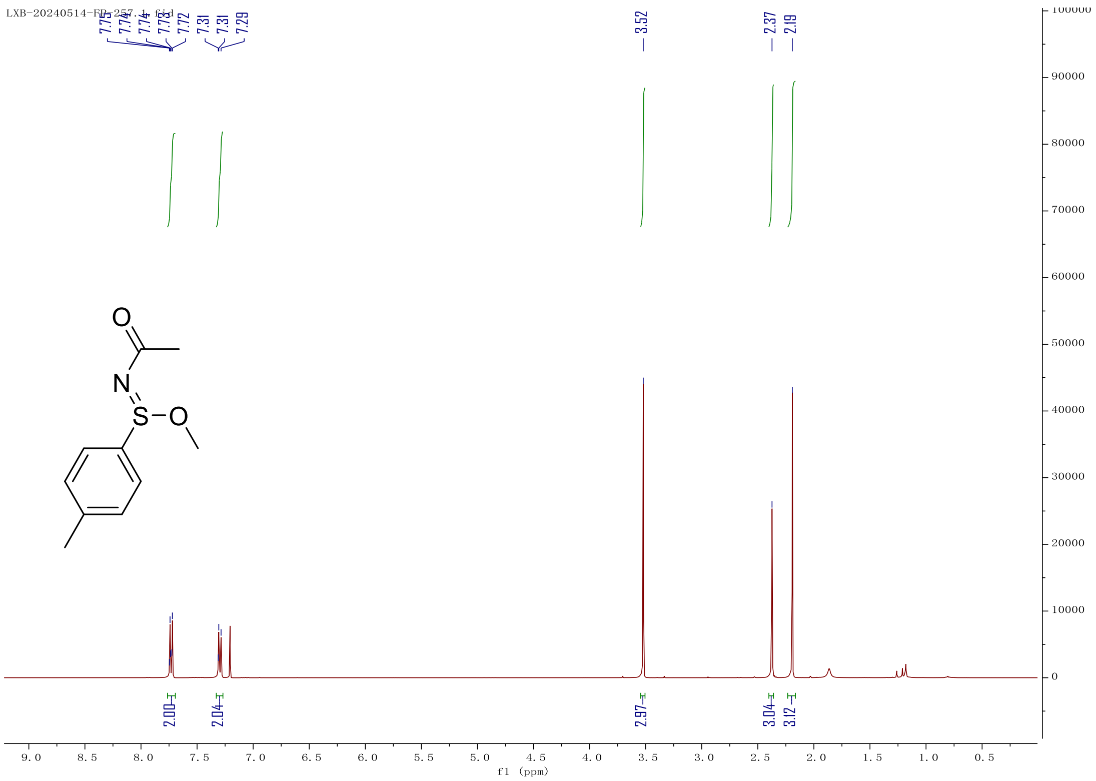

^{13}C NMR spectrum for compound **3t** (in $CDCl_3$, 101 MHz)

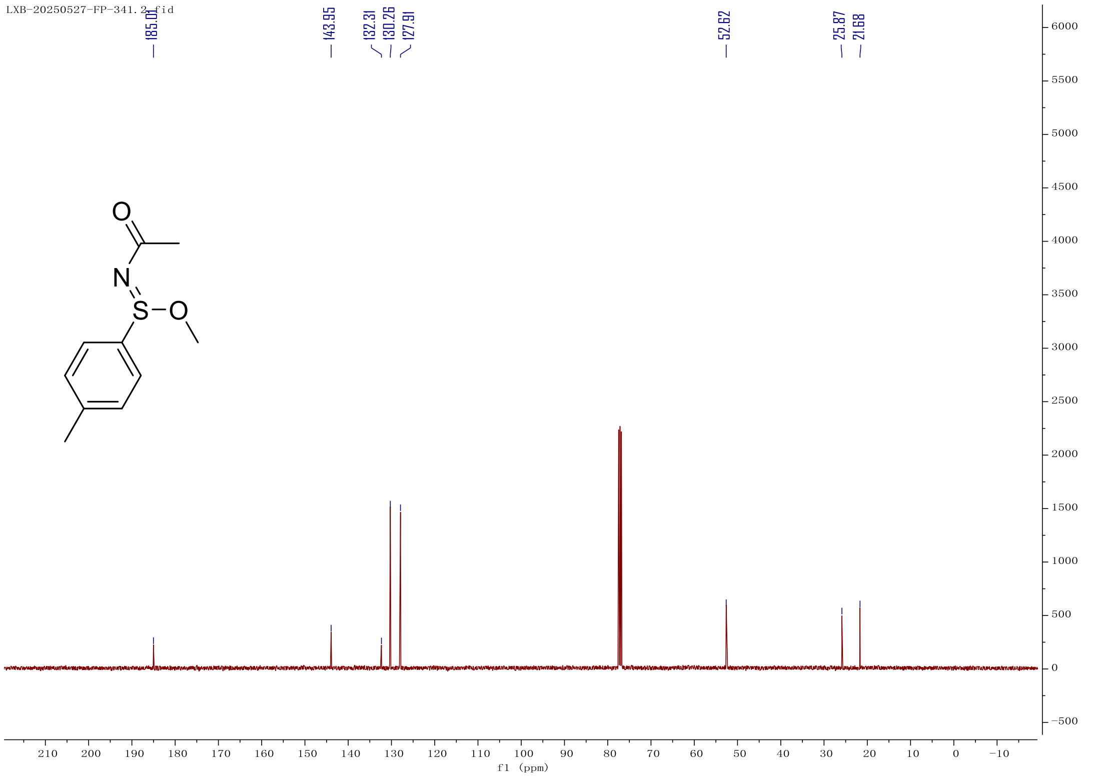

^{1}H NMR spectrum for compound **3u** (in CDCl3, 400 MHz)

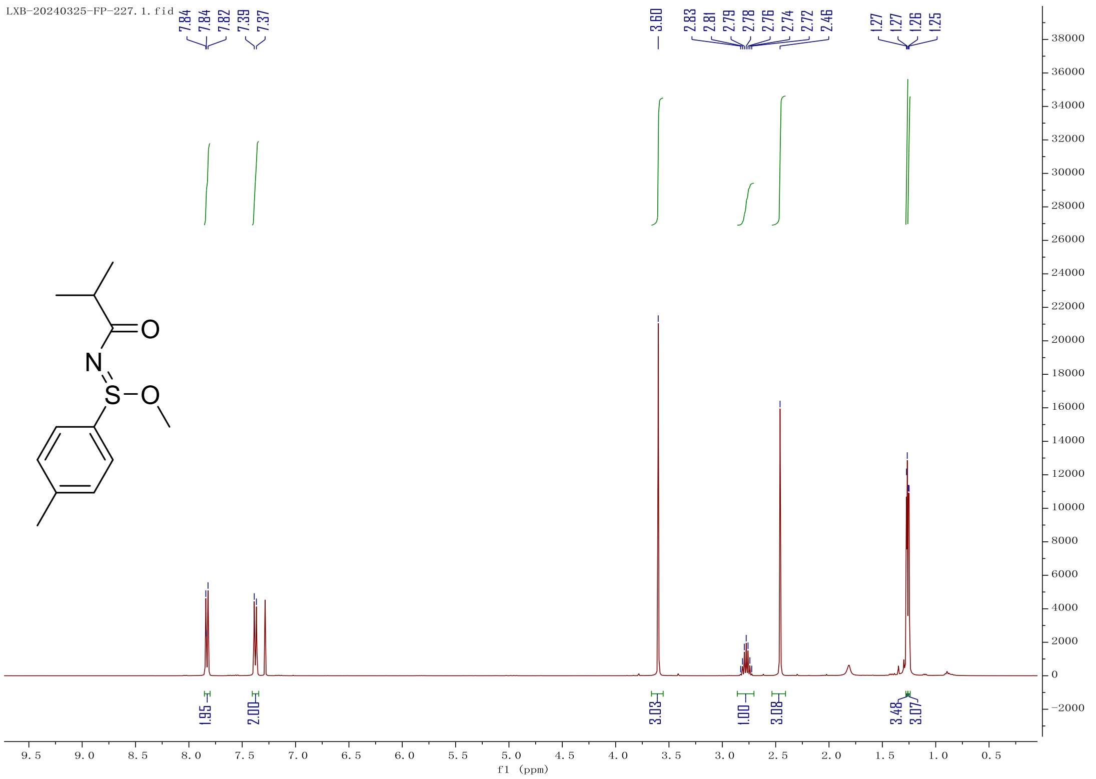

^{1}H NMR spectrum for compound **3v** (In CDCl3, 400 MHz)

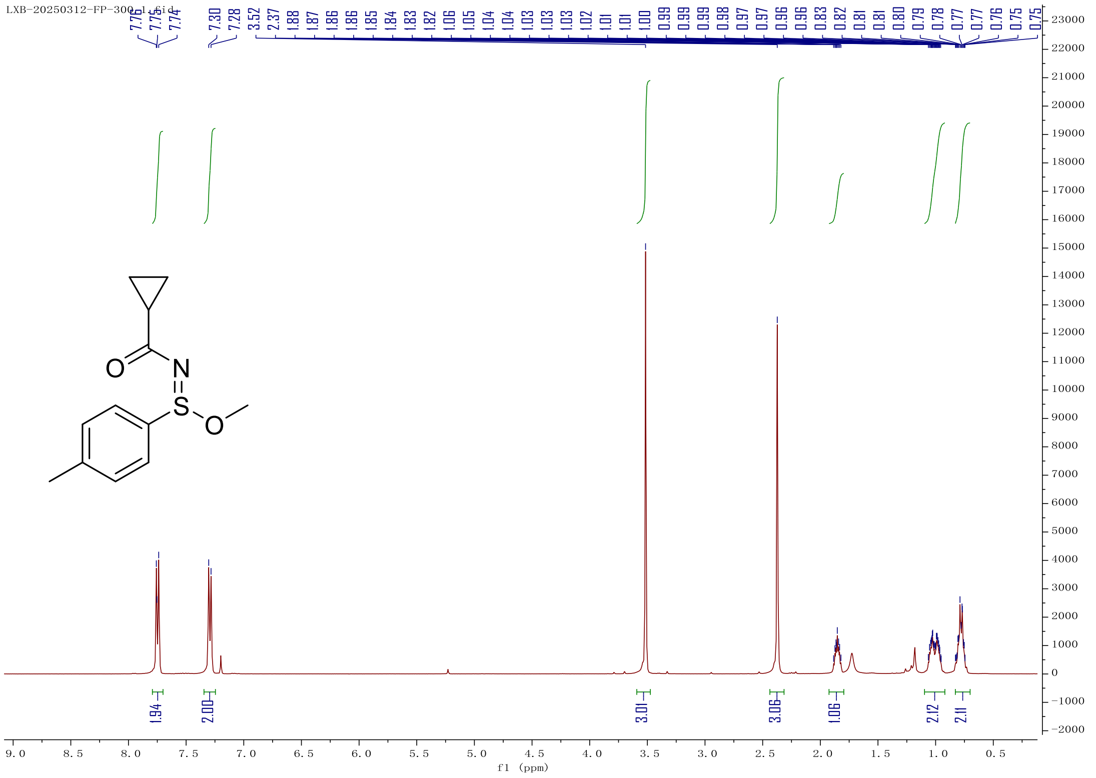

^{1}H NMR spectrum for compound **3w** (in $CDCl_3$, 400 MHz)

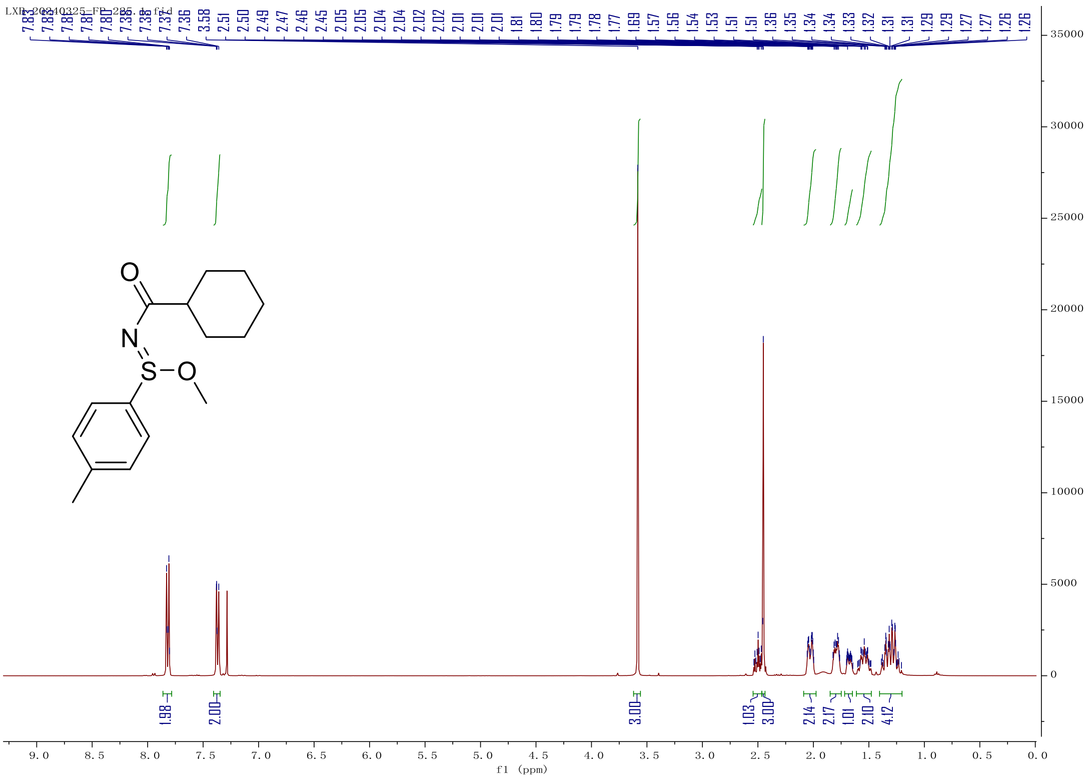

^{1}H NMR spectrum for compound **3x** (in $CDCl_3$, 400 MHz)

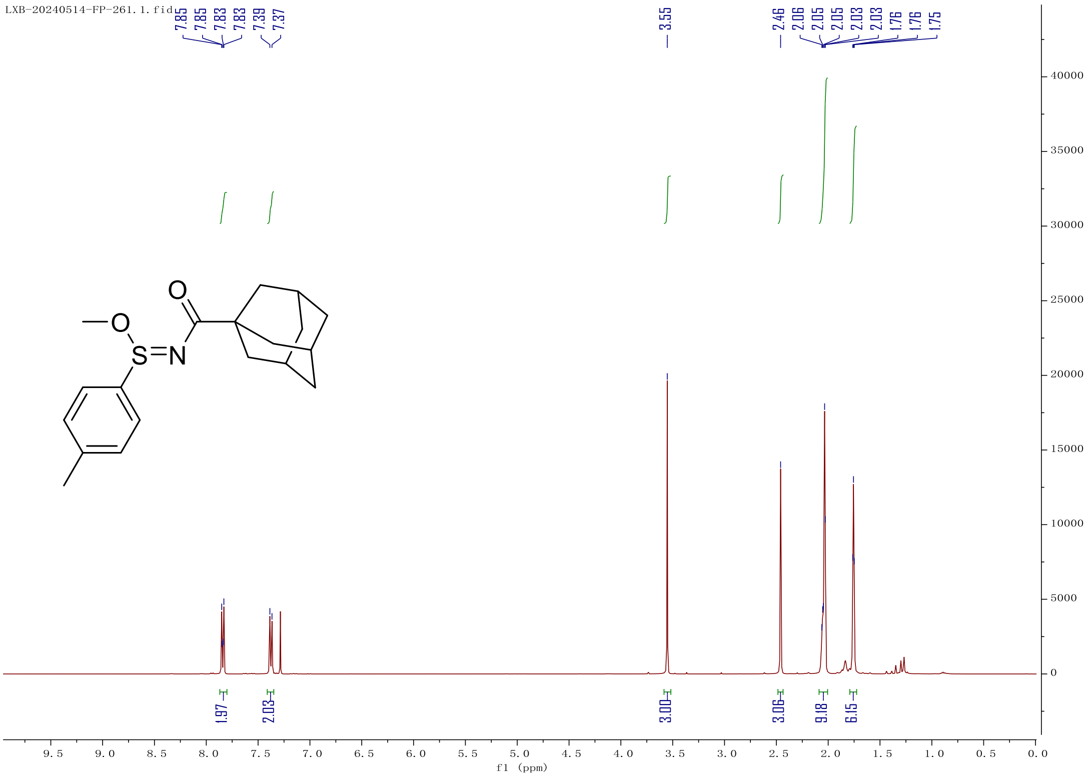

^{1}H NMR spectrum for compound **3y** (in $CDCl_3$, 400 MHz)

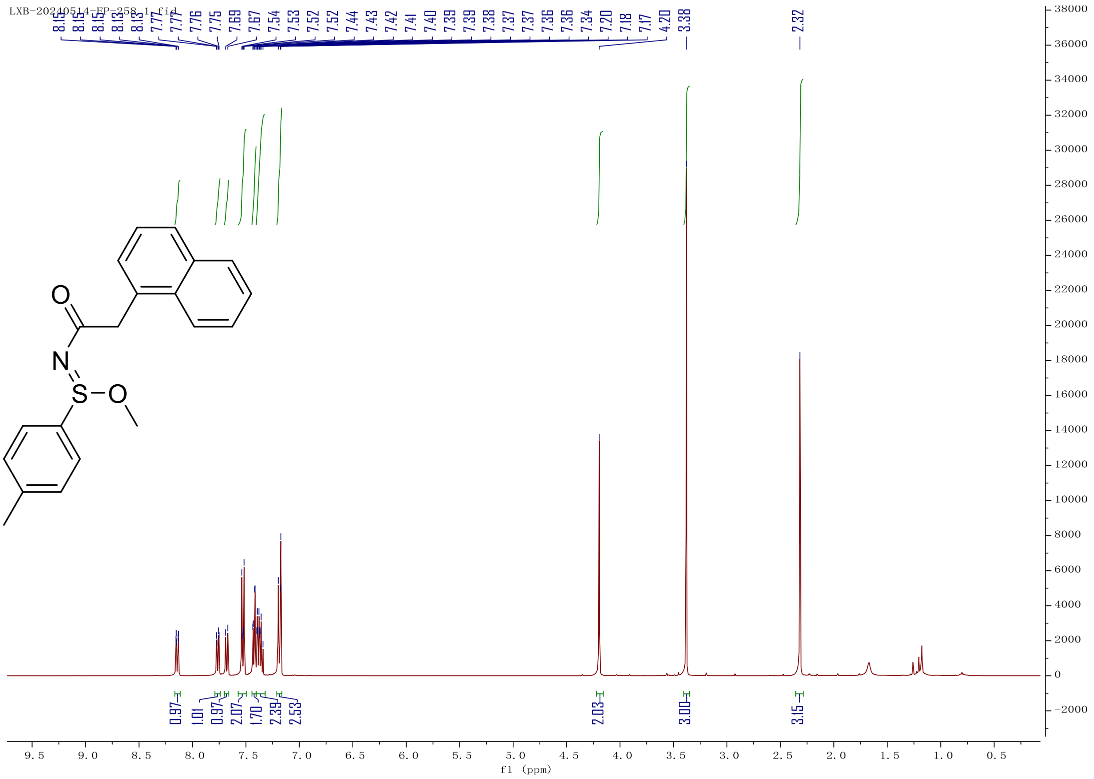

^{1}H NMR spectrum for compound **3z** (in $CDCl_3$, 400 MHz)

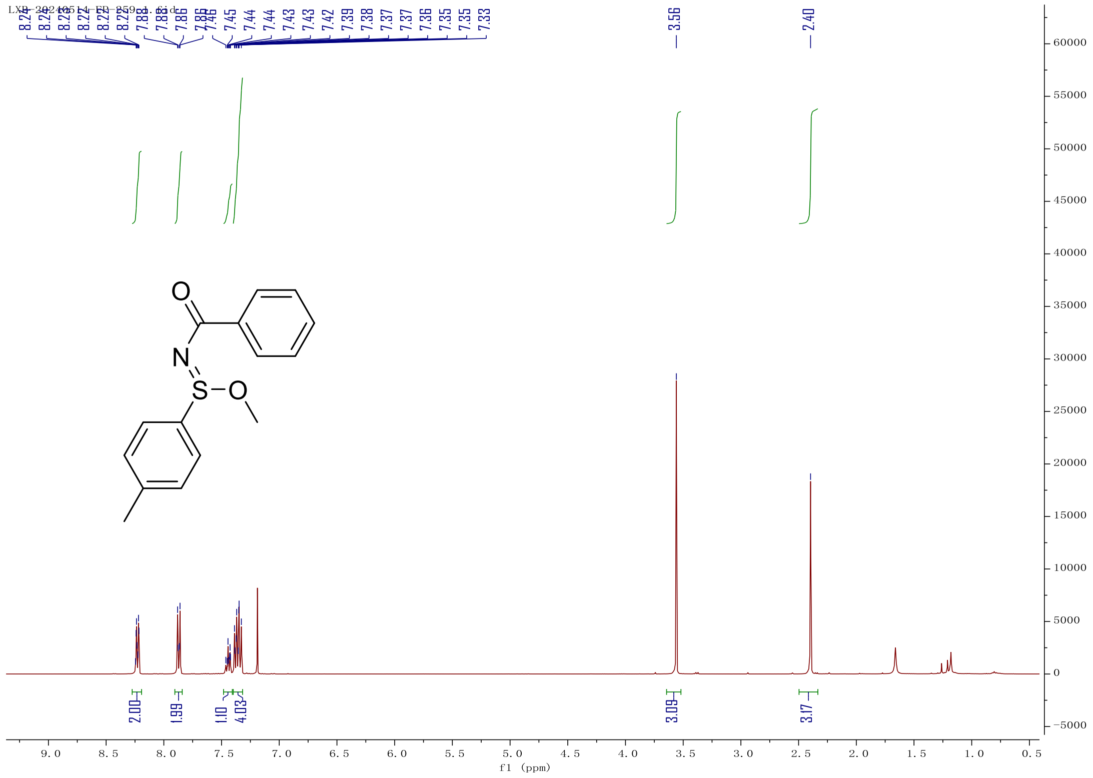

^{1}H NMR spectrum for compound **3a’** (in CDCl3, 400 MHz)

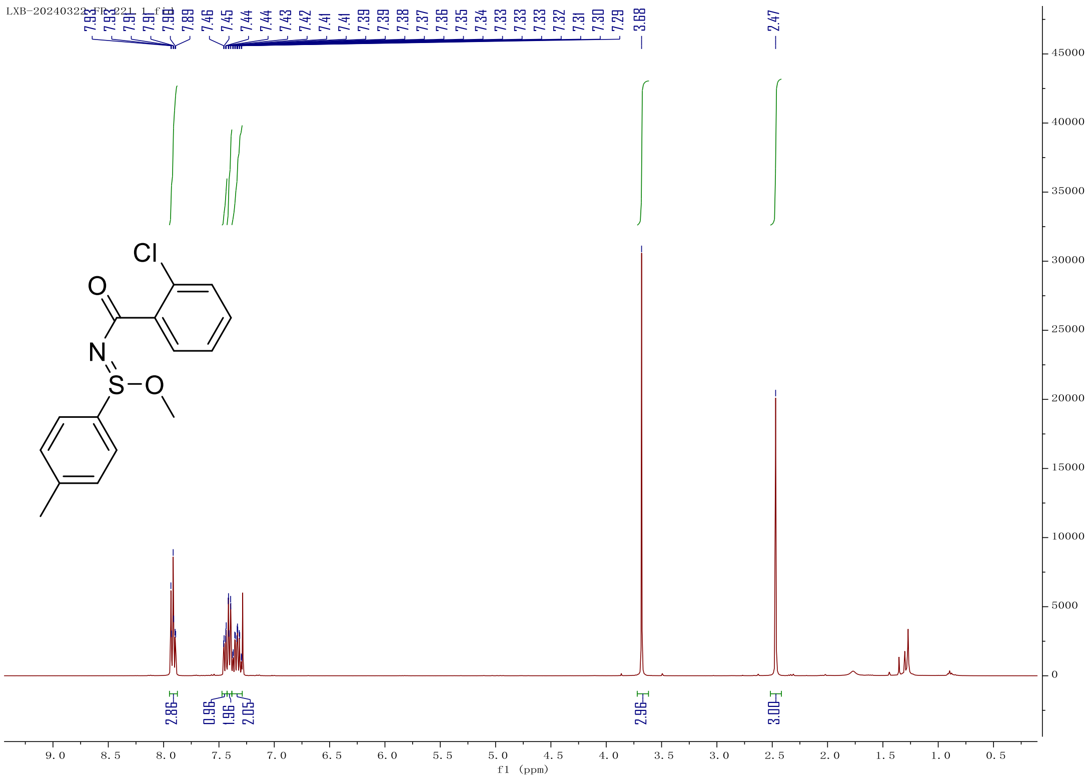

^{13}C NMR spectrum for compound **3a’** (in CDCl3, 101 MHz)

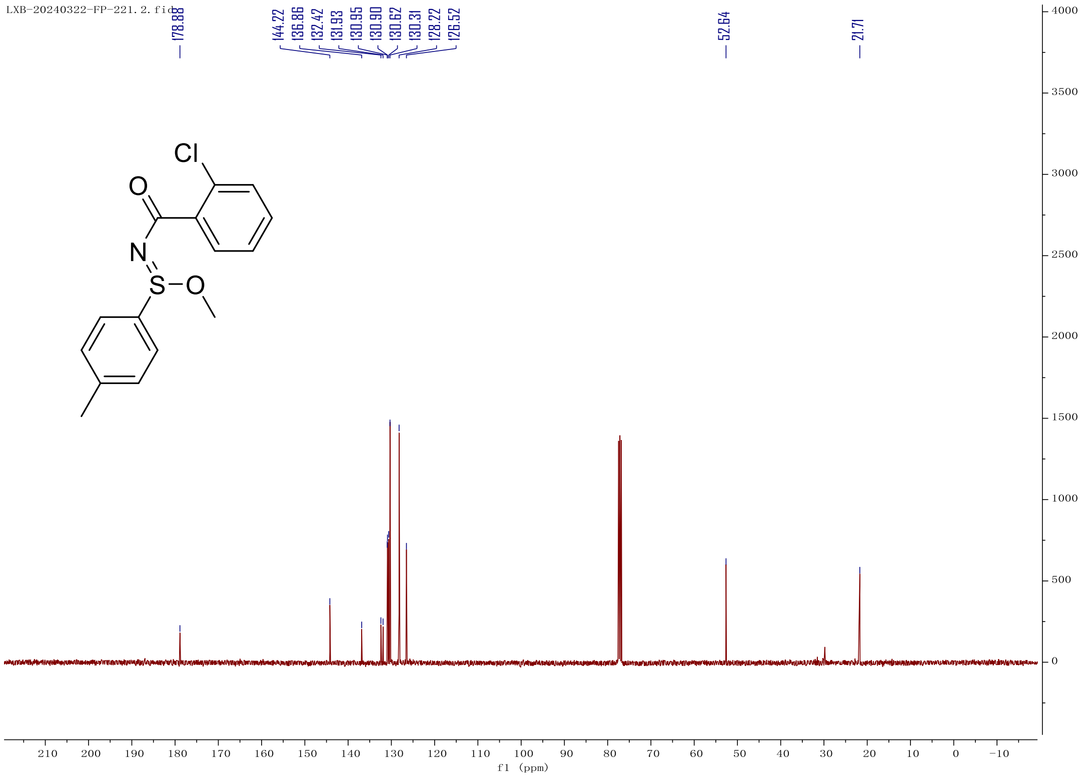

^{1}H NMR spectrum for compound **3b'** (in $CDCl_3$, 400 MHz)

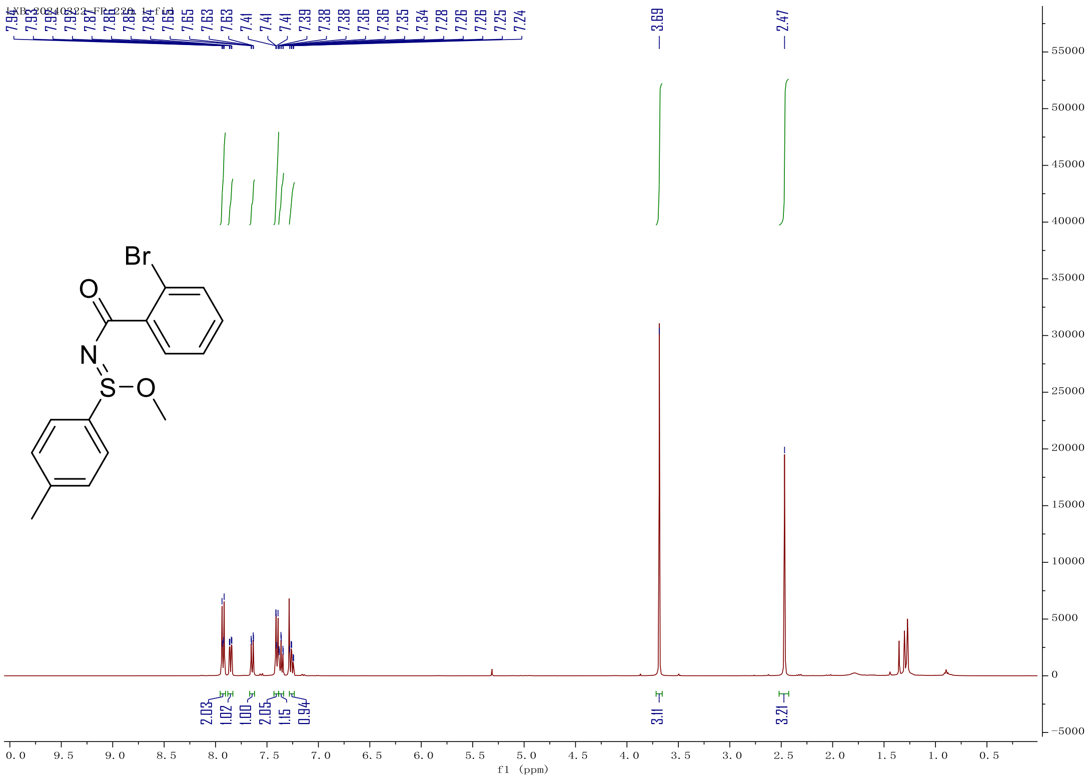

^{13}C NMR spectrum for compound **3b'** (in $CDCl_3$, 101 MHz)

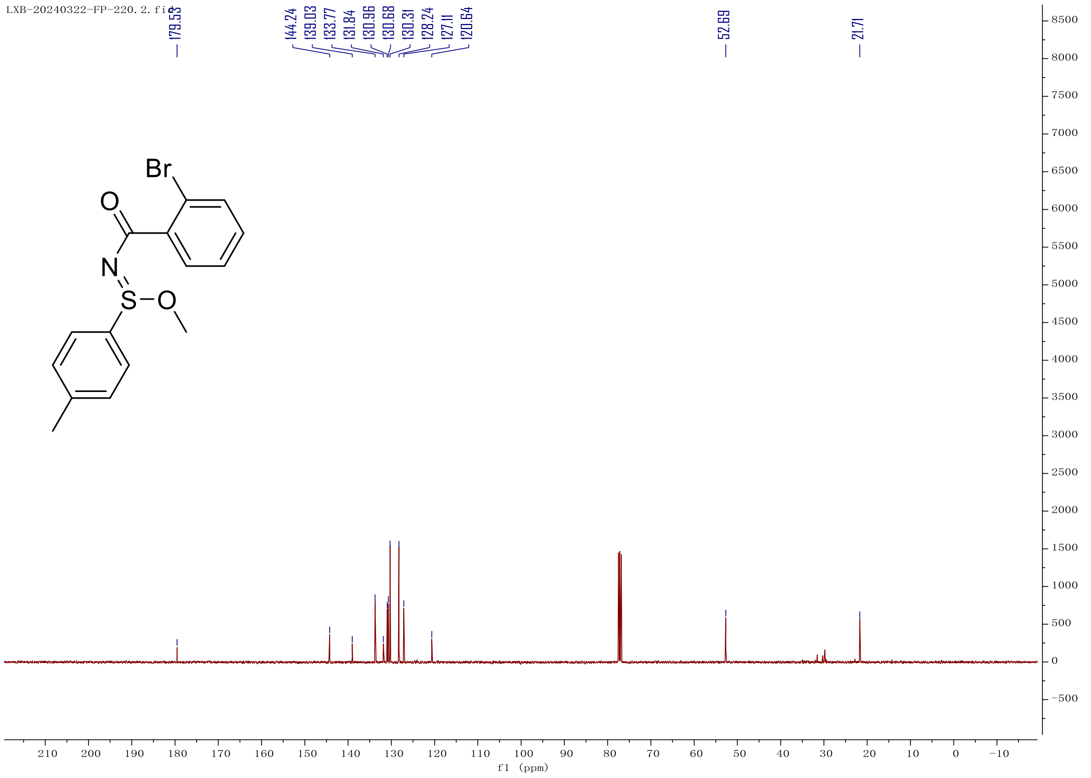

^{1}H NMR spectrum for compound **3c’** (in CDCl3, 400 MHz)

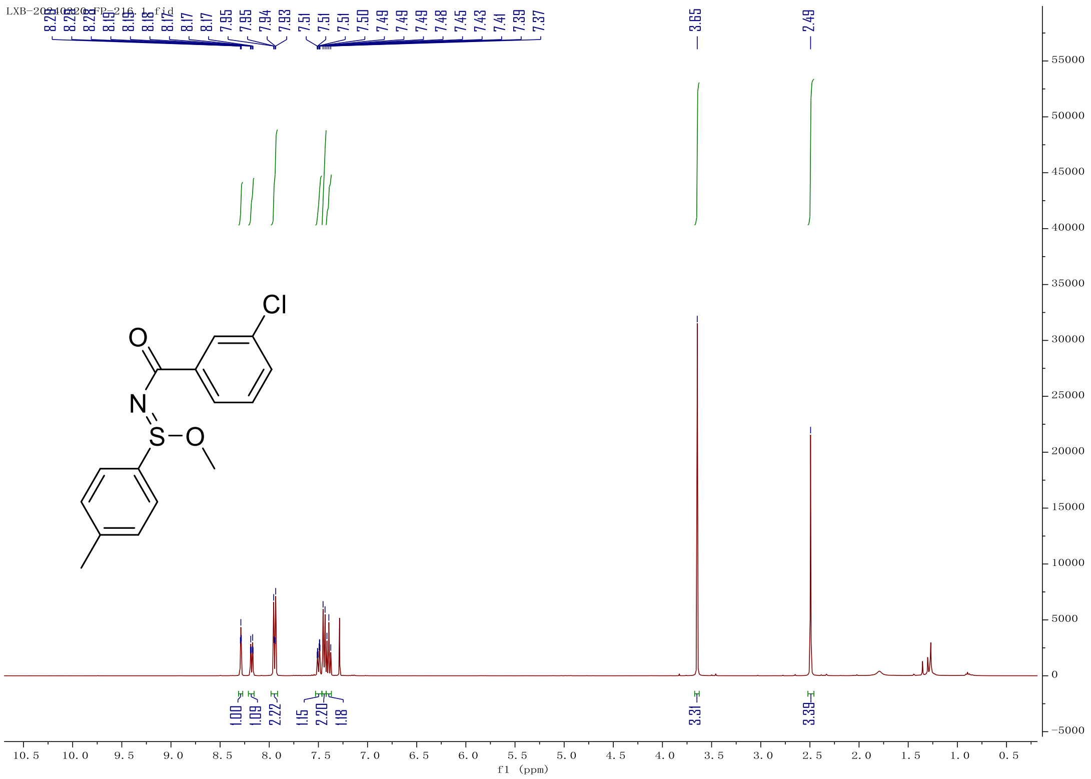

^{13}C NMR spectrum for compound **3c’** (in CDCl3, 101 MHz)

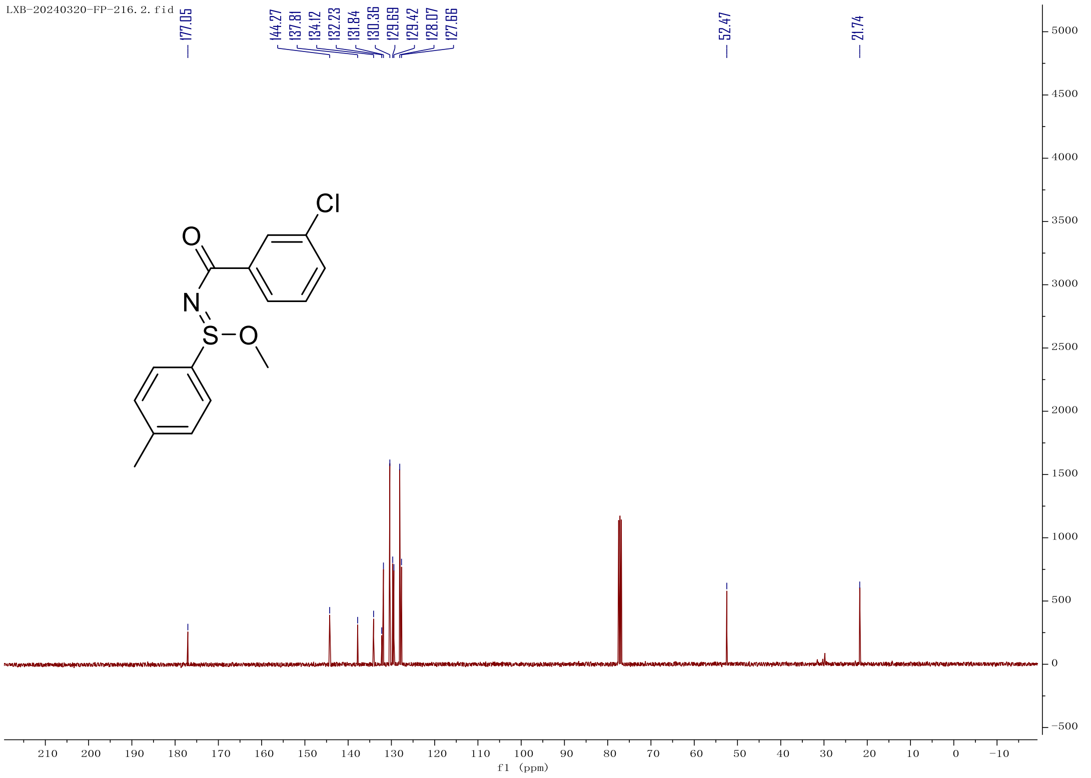

^{1}H NMR spectrum for compound **3d'** (in CDCl3, 400 MHz)

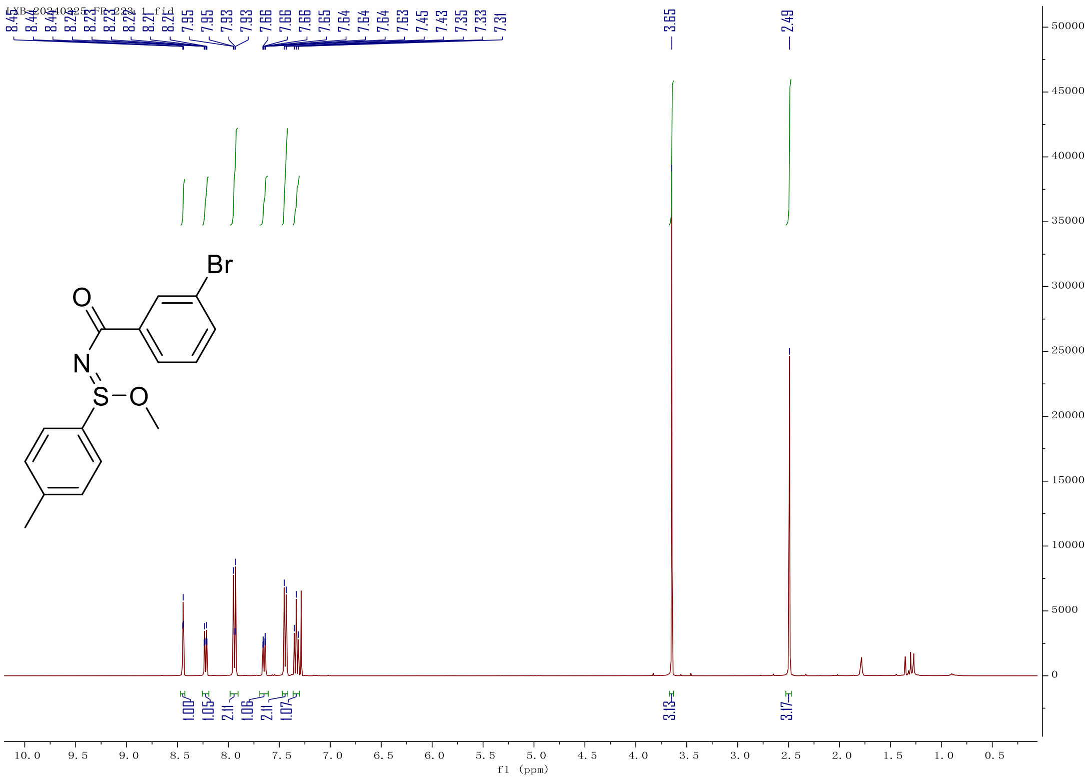

^{13}C NMR spectrum for compound **3d'** (in CDCl3, 101 MHz)

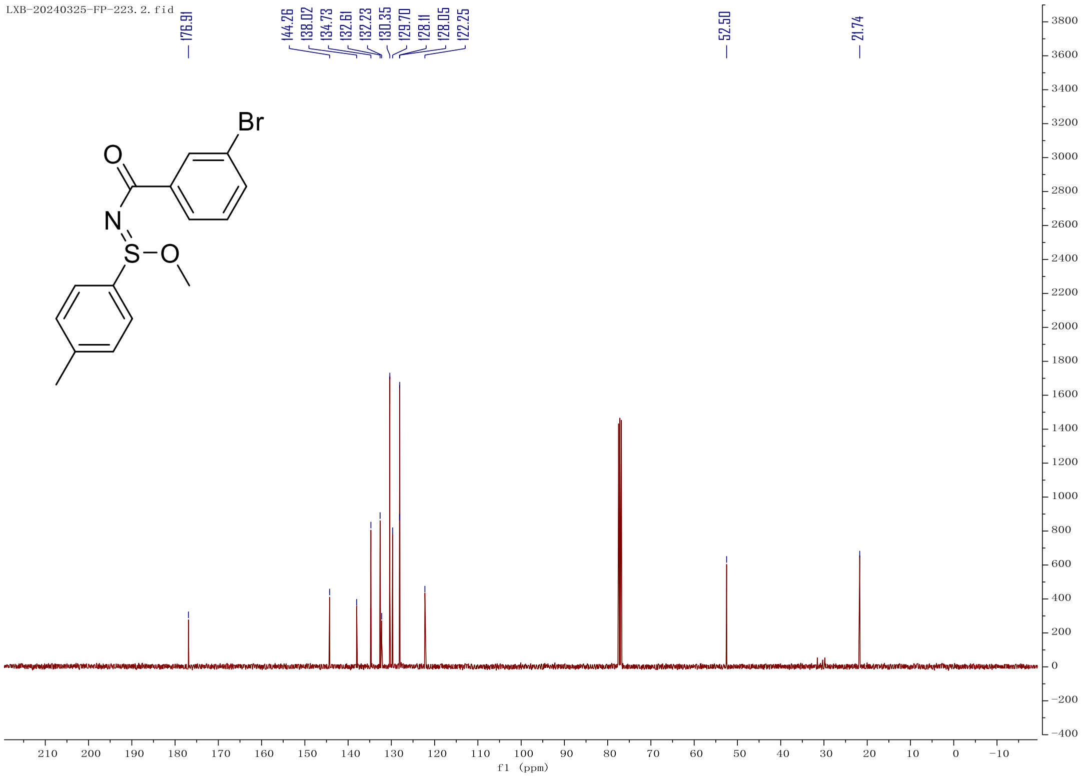

^{1}H NMR spectrum for compound **3e'** (in CDCl3, 400 MHz)

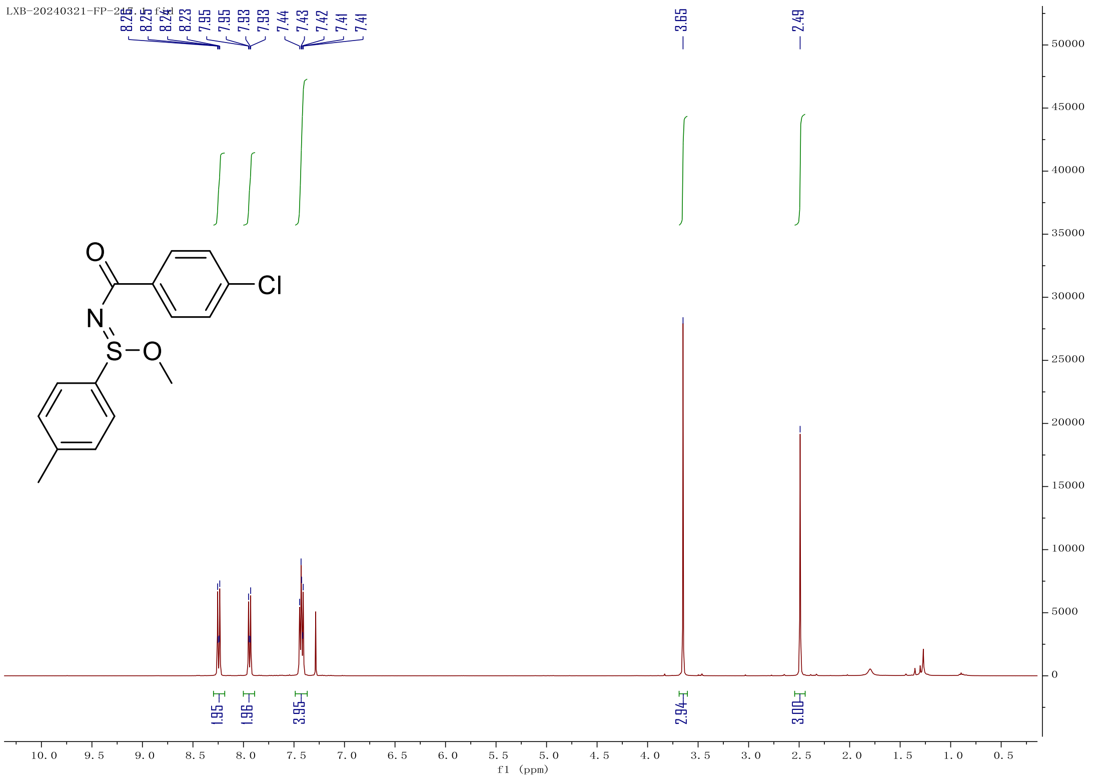

^{13}C NMR spectrum for compound **3e'** (in CDCl3, 101 MHz)

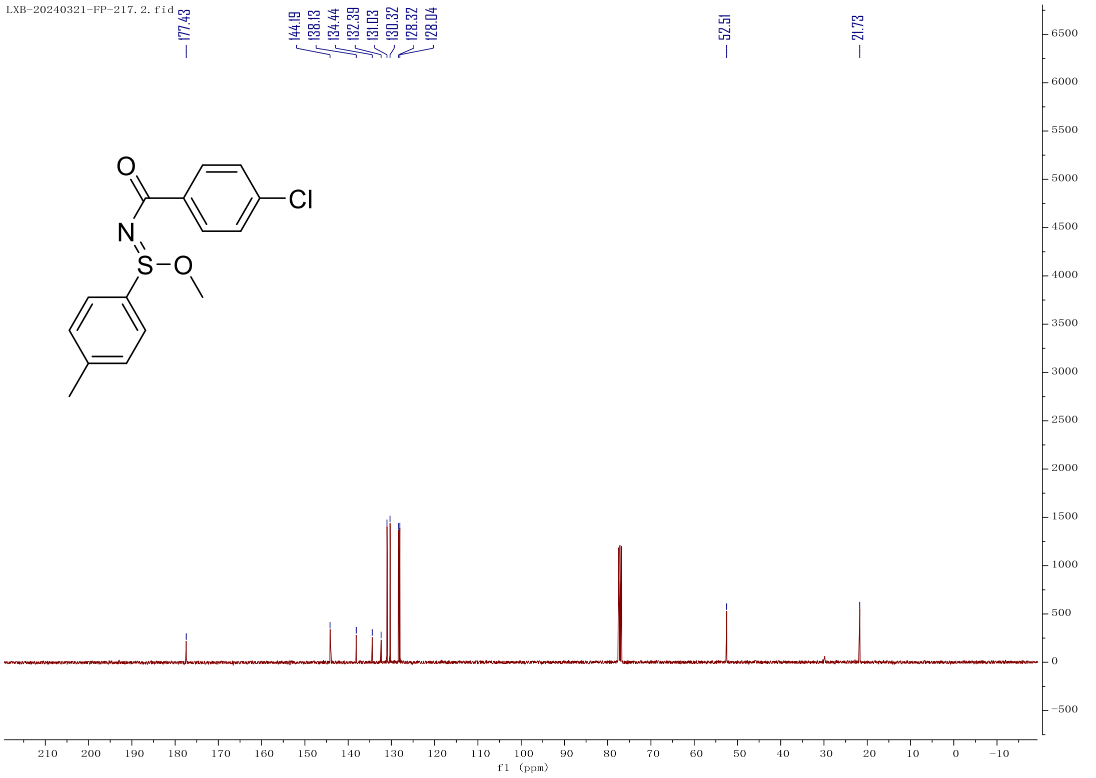

^{1}H NMR spectrum for compound **3f'** (in CDCl3, 400 MHz)

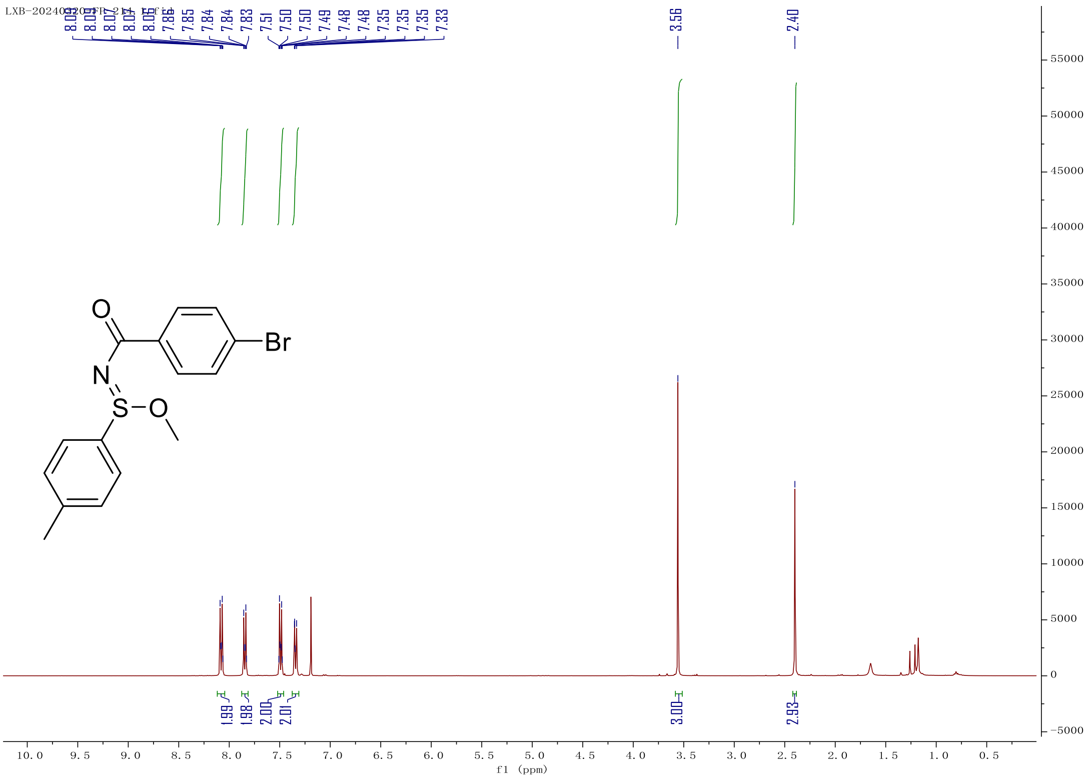

^{13}C NMR spectrum for compound **3f'** (in CDCl3, 101 MHz)

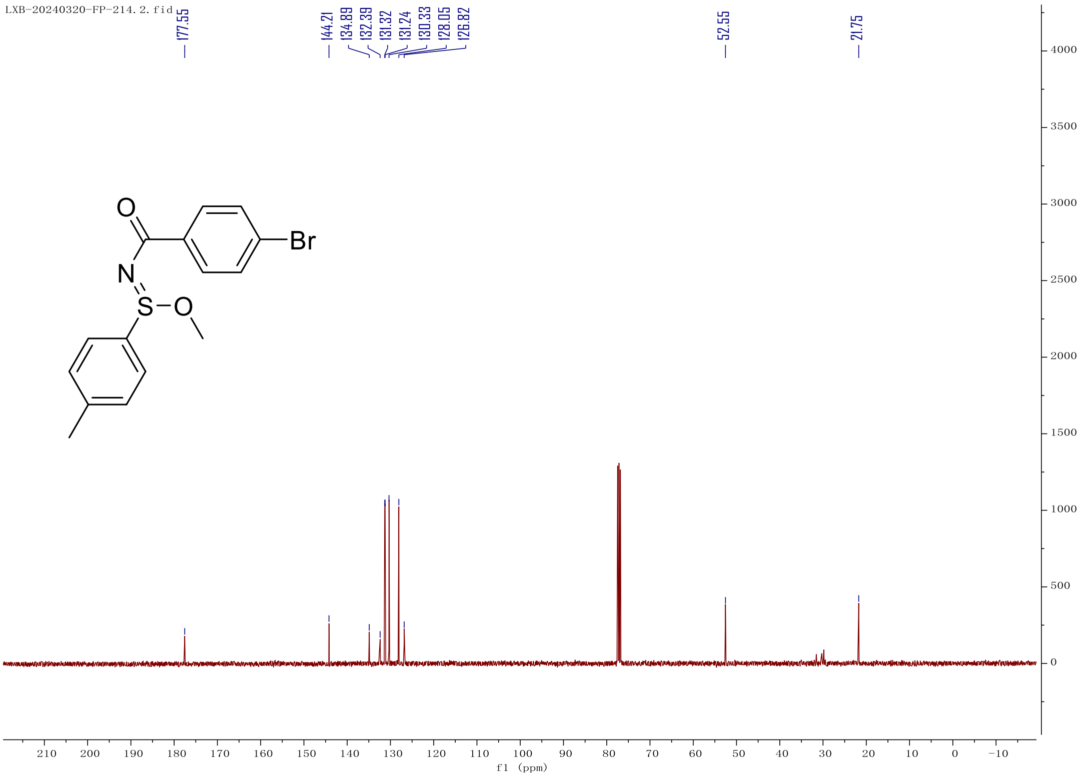

^{1}H NMR spectrum for compound **3g'** (in $CDCl_3$, 400 MHz)

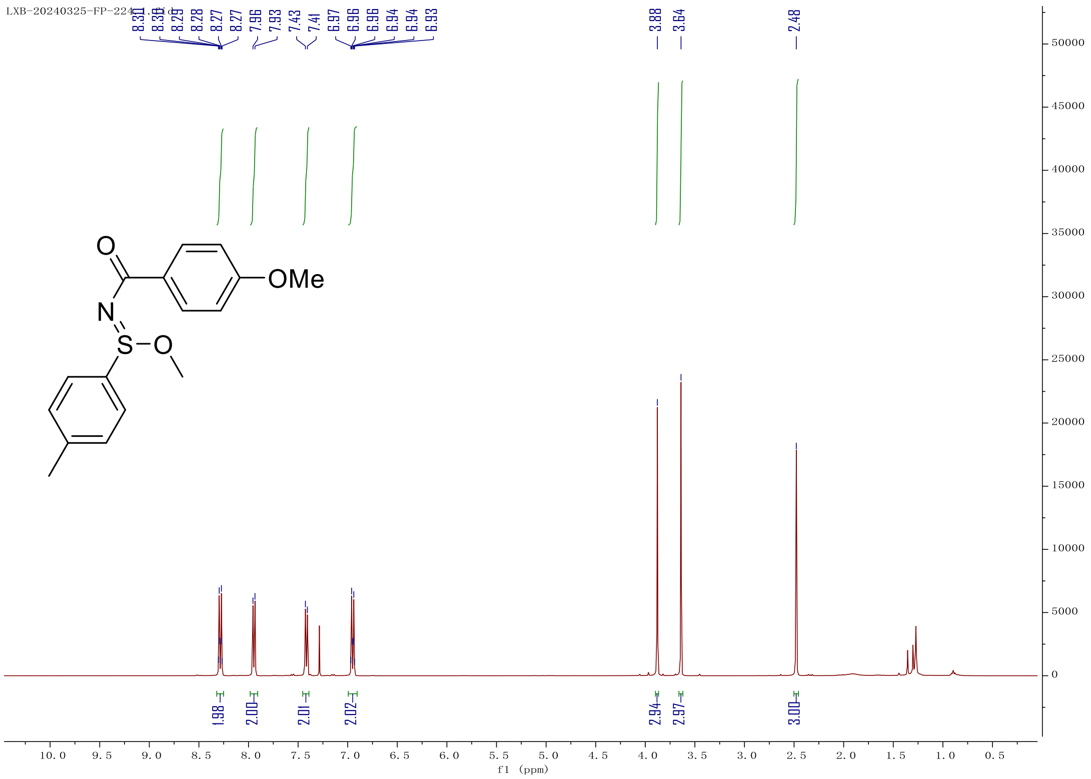

^{1}H NMR spectrum for compound **3h'** (in CDCl3, 400 MHz)

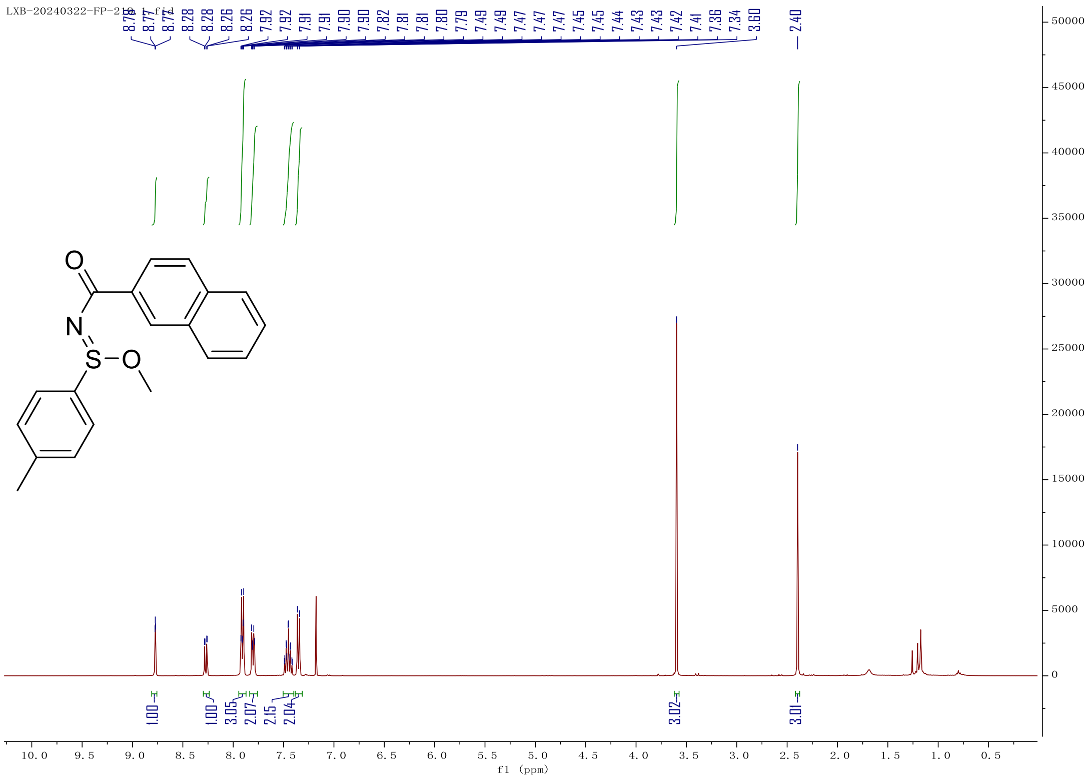

^{13}C NMR spectrum for compound **3h'** (in CDCl3, 101 MHz)

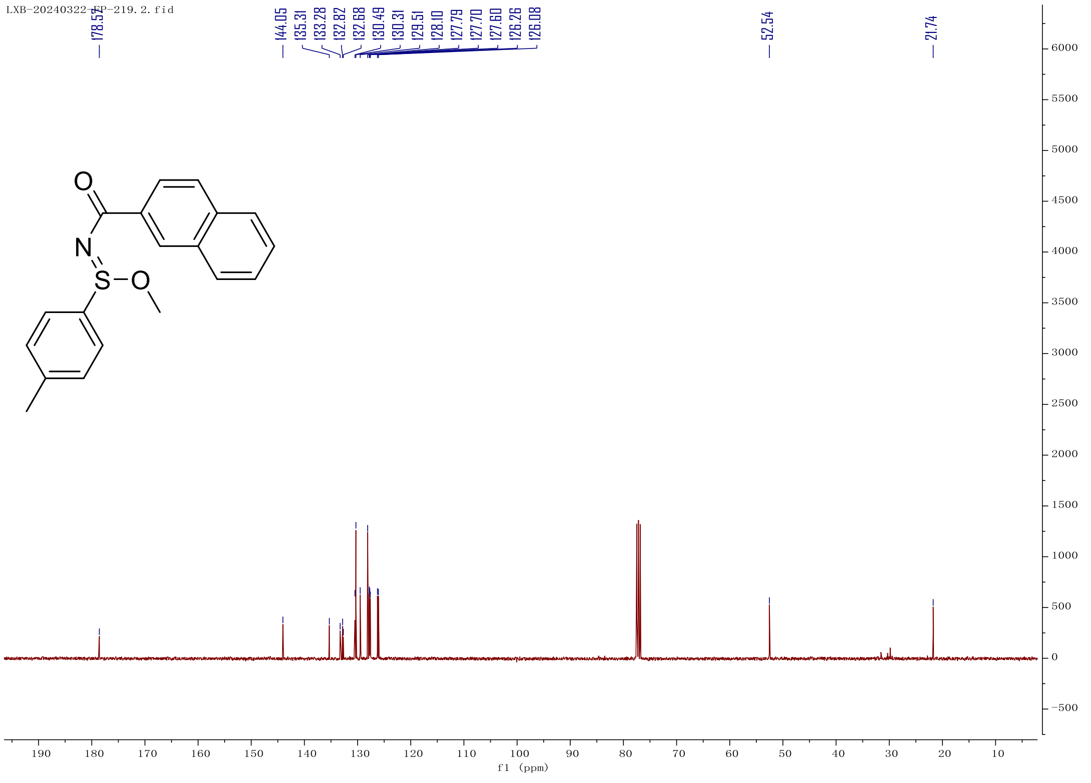

^{1}H NMR spectrum for compound **3i'** (in $CDCl_3$, 400 MHz)

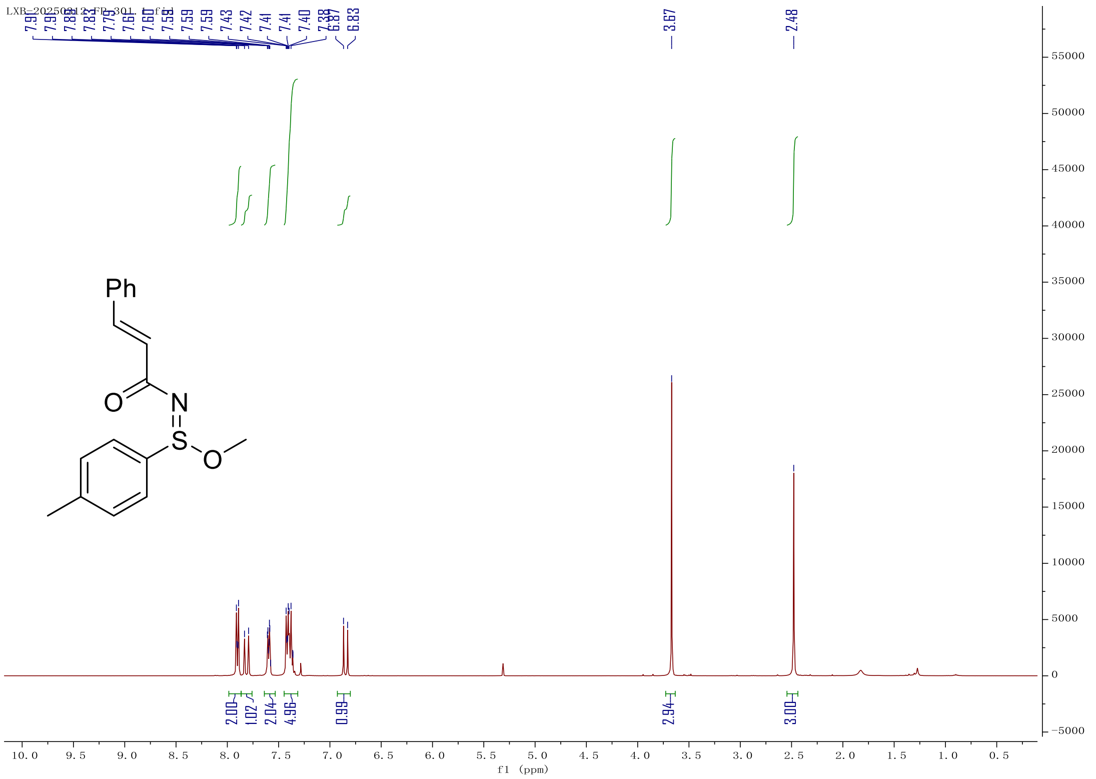

^1H NMR spectrum for compound **3j’** (in $CDCl_3$, 400 MHz)

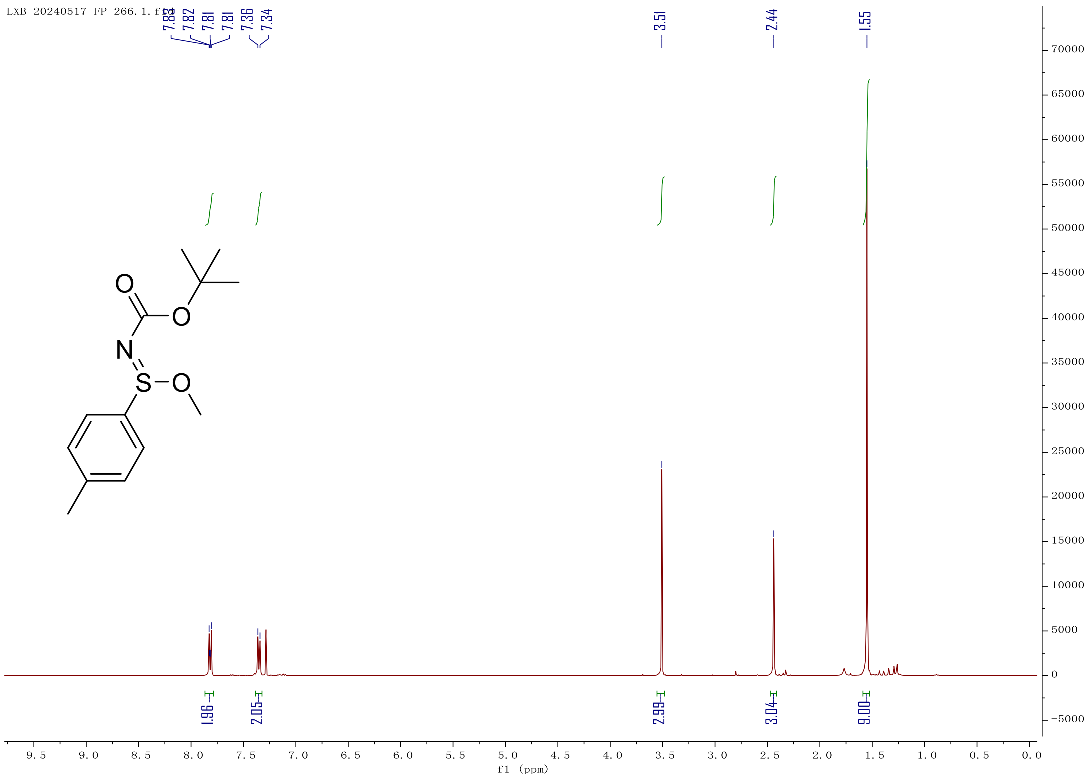

^{13}C NMR spectrum for compound **3j’** (in $CDCl_3$, 101 MHz)

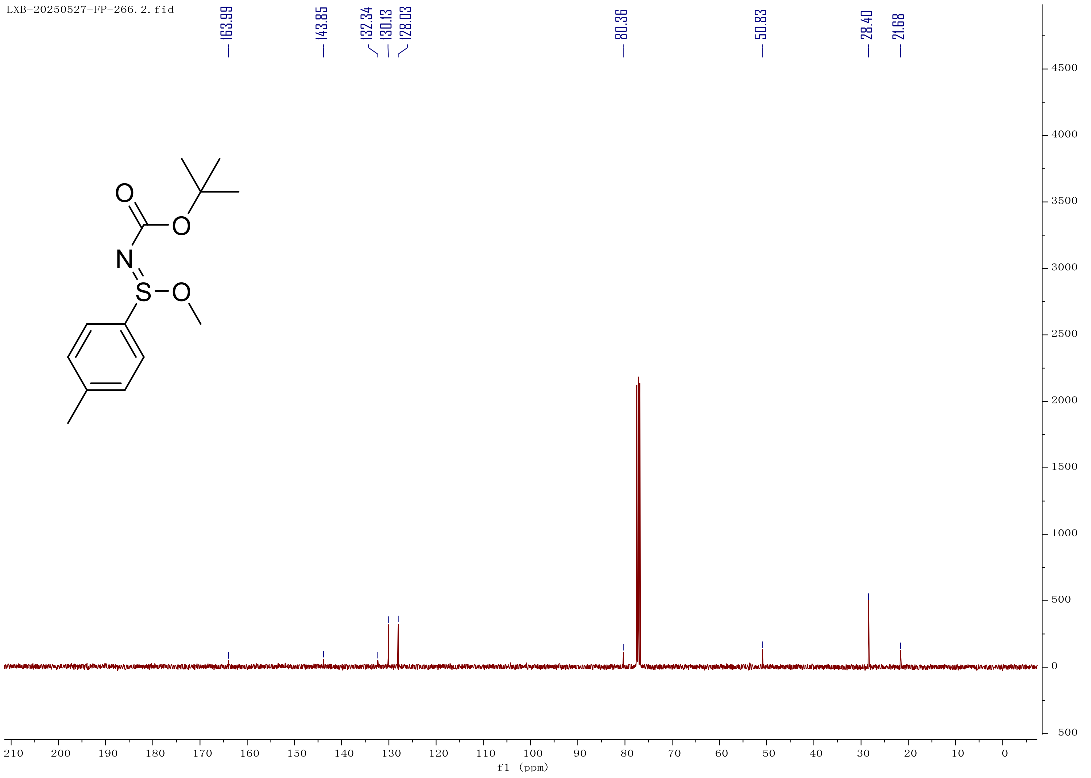

^{1}H NMR spectrum for compound **3k’** (in CDCl3, 400 MHz)

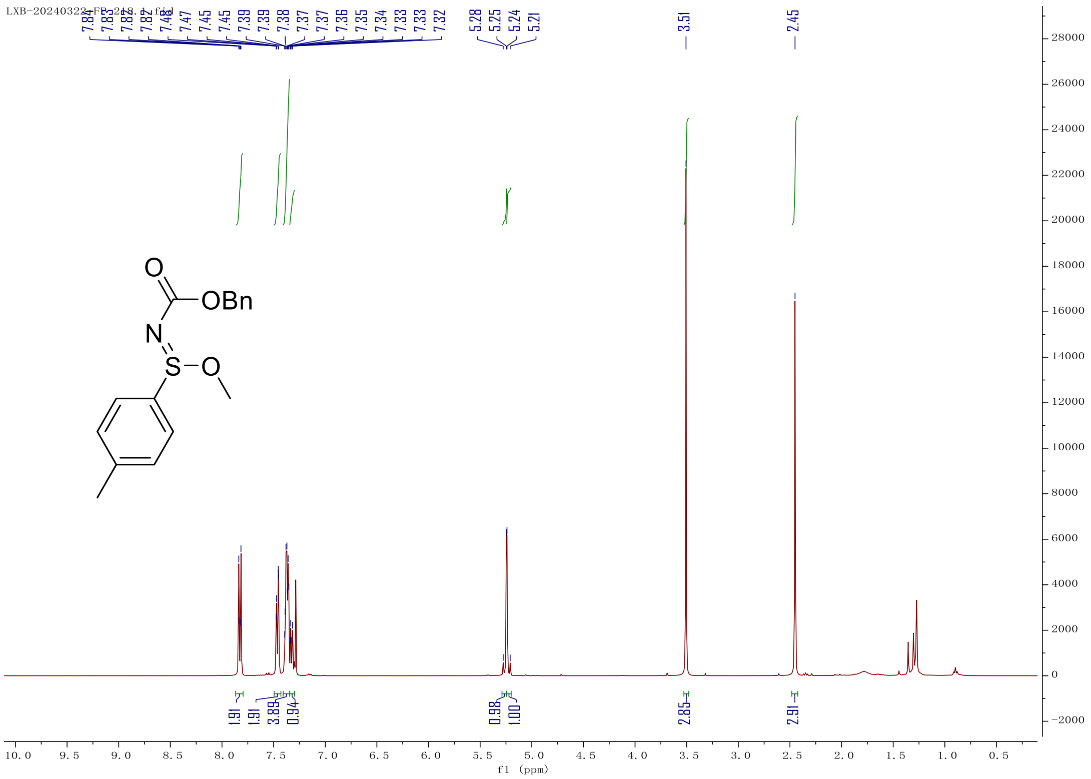

^{1}H NMR spectrum for compound **3l’** (in CDCl3, 400 MHz)

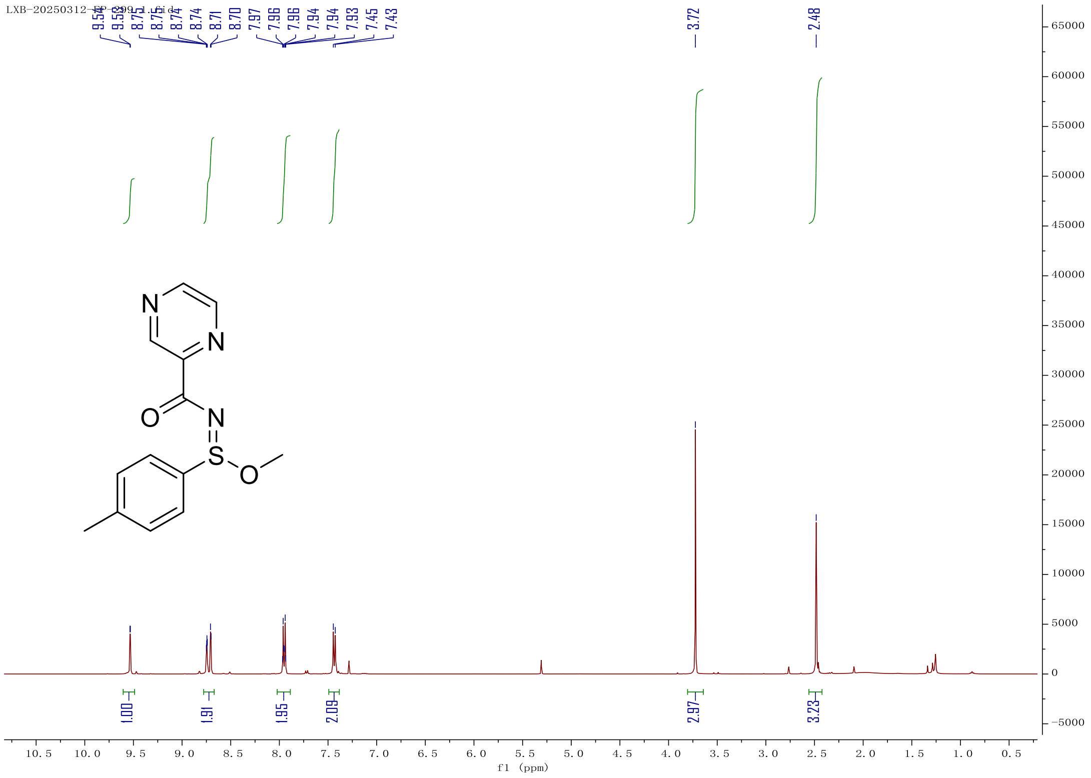

^{1}H NMR spectrum for compound **3m’** (in CDCl3, 400 MHz)

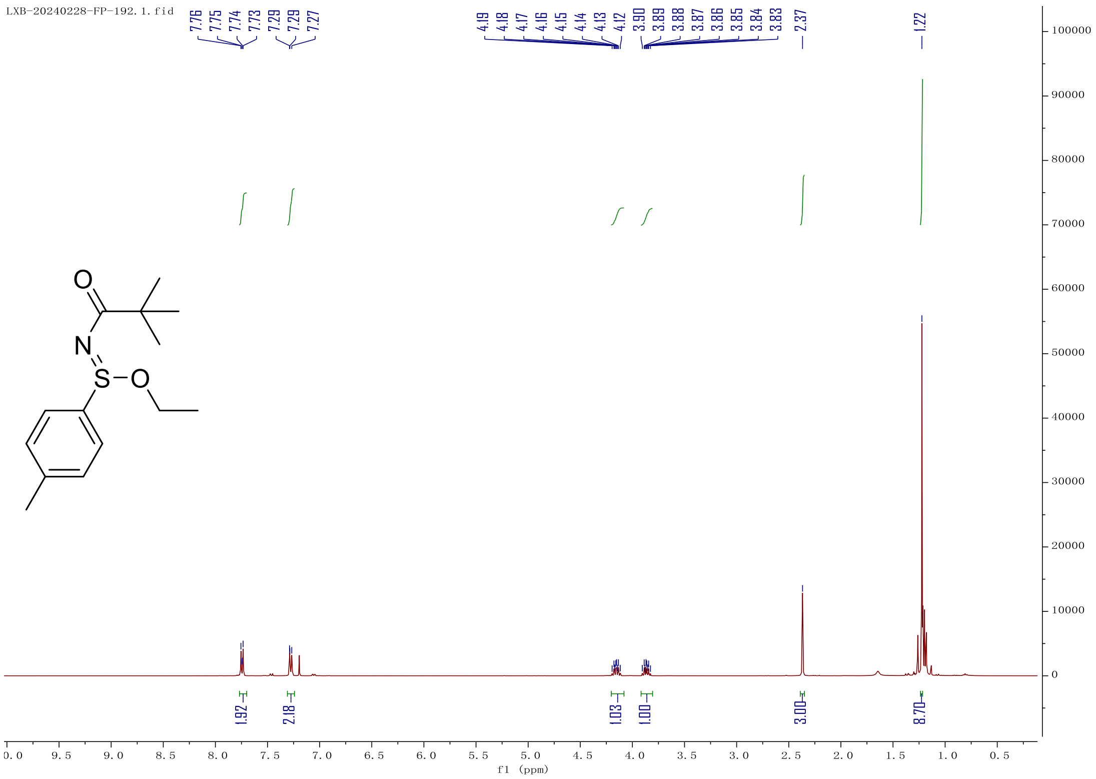

^{1}H NMR spectrum for compound **3n’** (in CDCl3, 400 MHz)

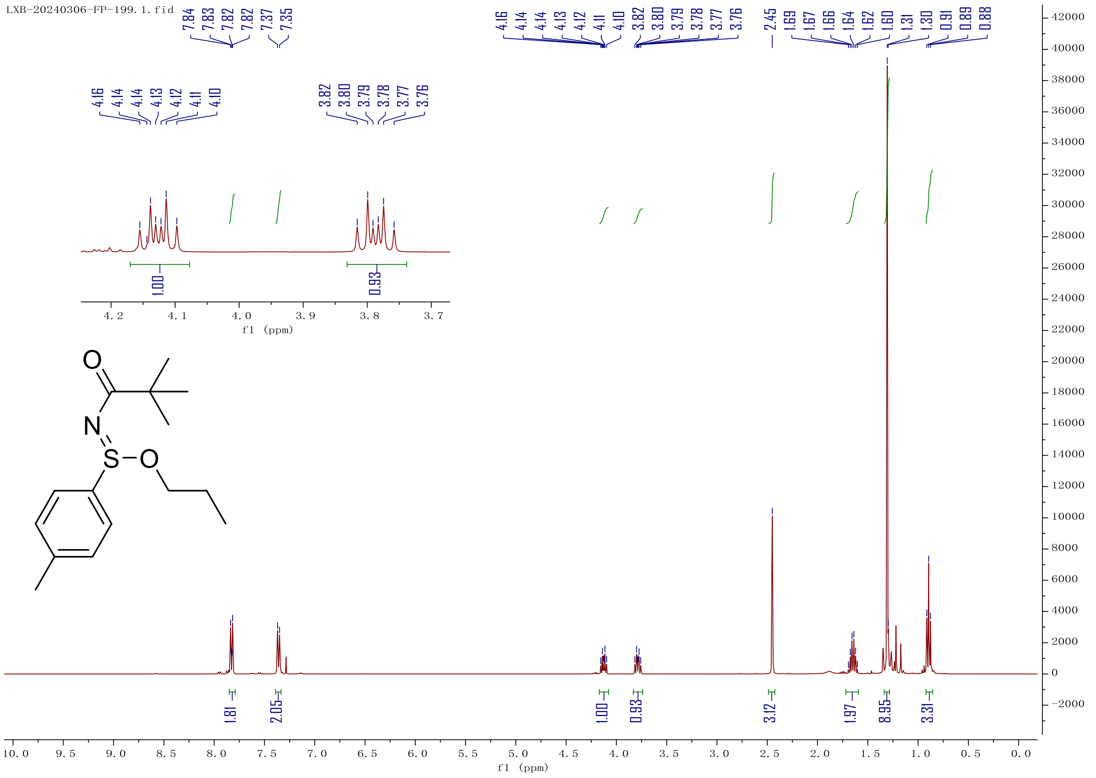

^{1}H NMR spectrum for compound **3o'** (in $CDCl_3$, 400 MHz)

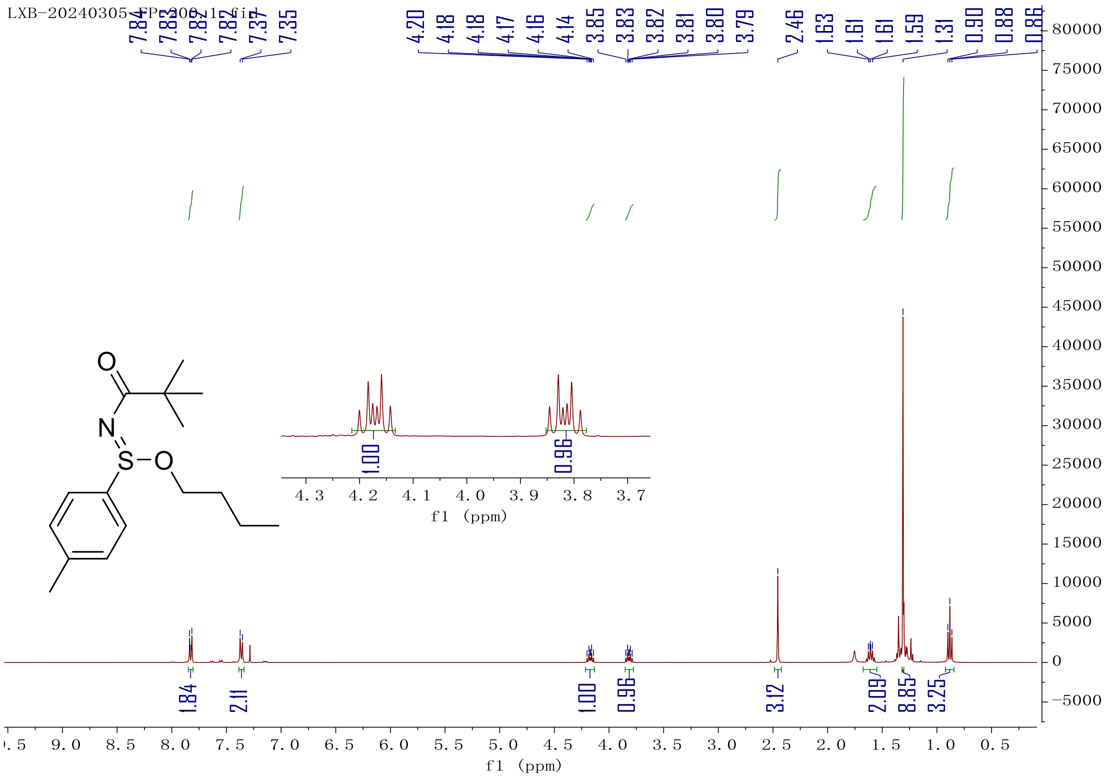

^{1}H NMR spectrum for compound **3p'** (in $CDCl_3$, 400 MHz)

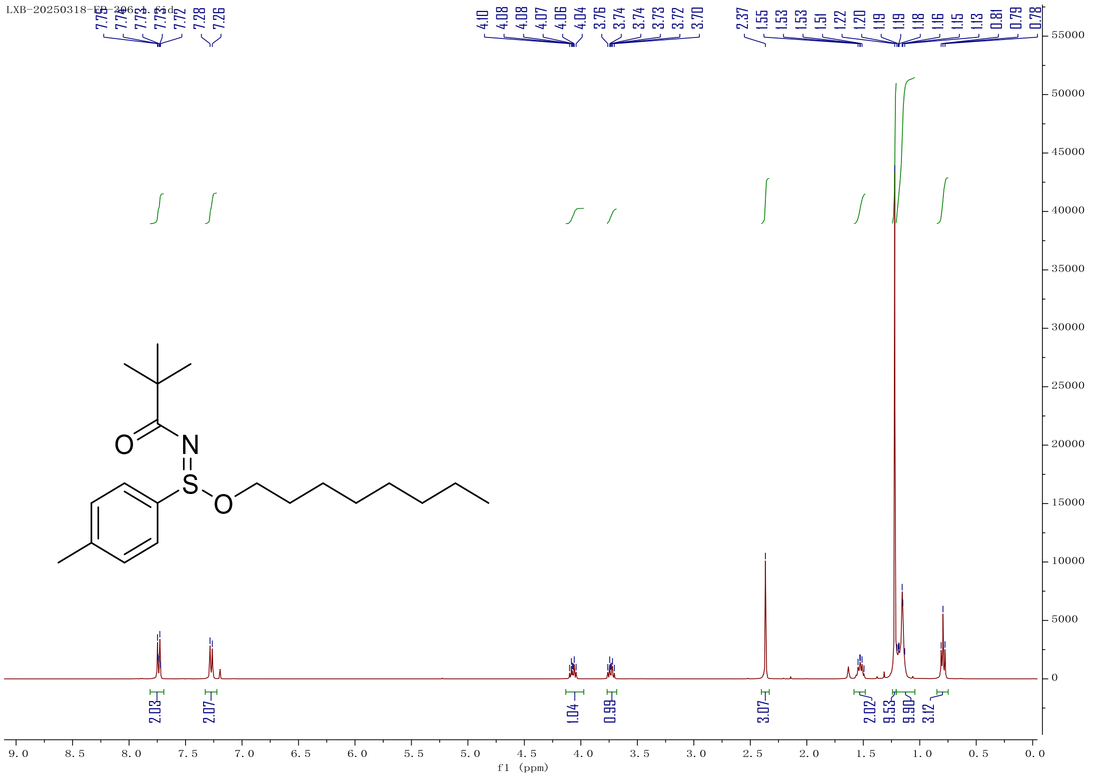

^{1}H NMR spectrum for compound **3q'** (in $CDCl_3$, 400 MHz)

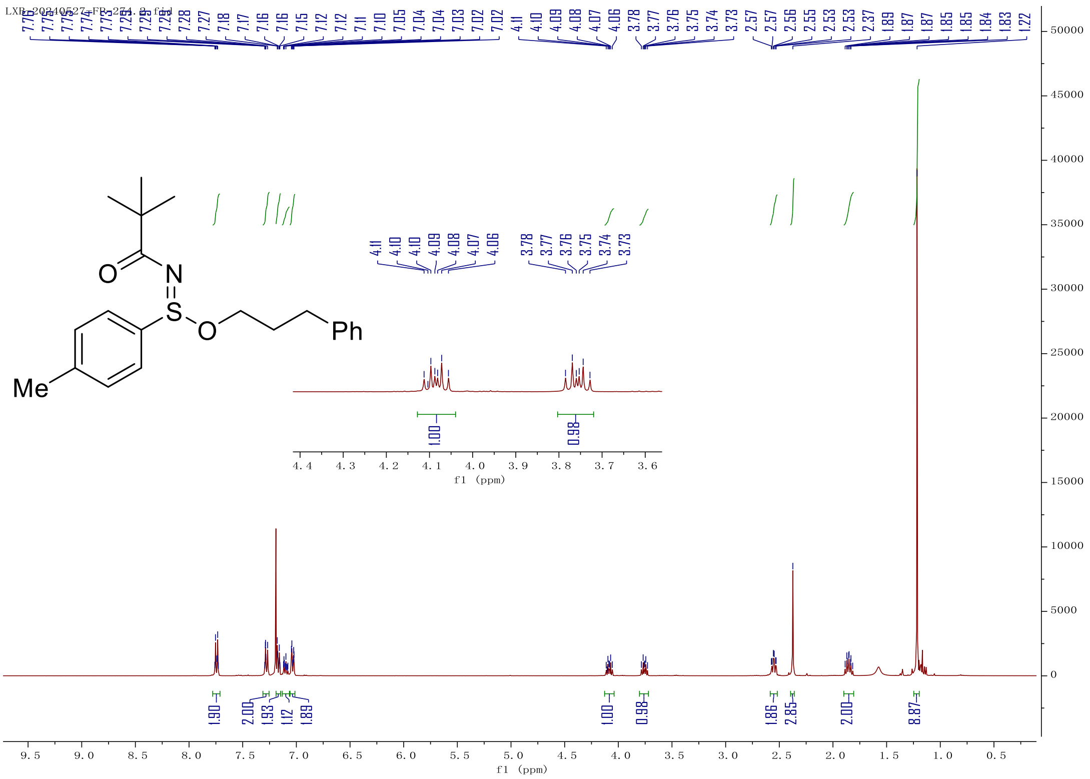

^{1}H NMR spectrum for compound **3r'** (in $CDCl_3$, 400 MHz)

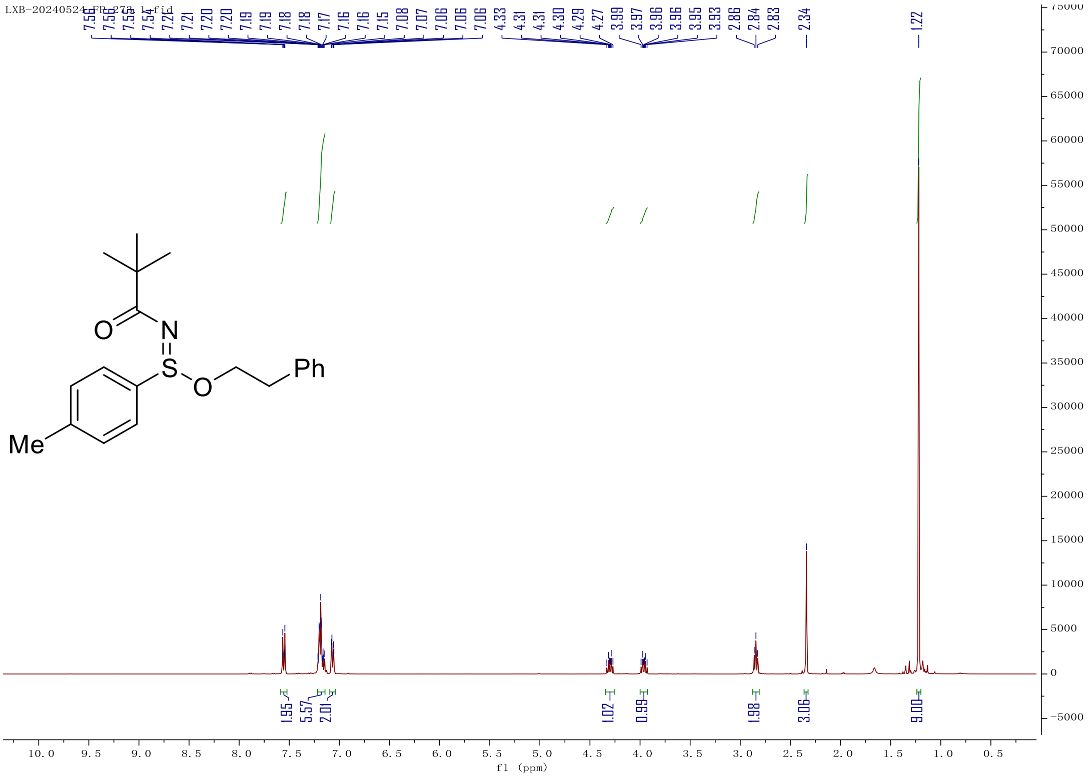

^{1}H NMR spectrum for compound **3s'** (in $CDCl_3$, 400 MHz)

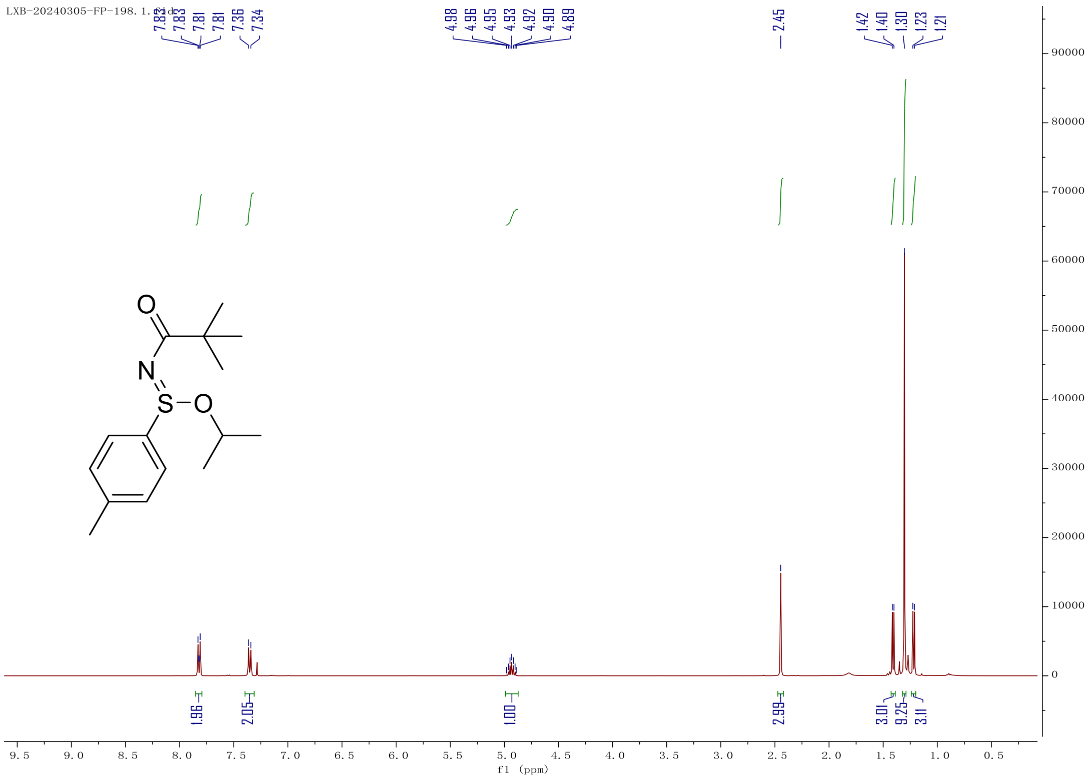

^{13}C NMR spectrum for compound **3s'** (in $CDCl_3$, 101 MHz)

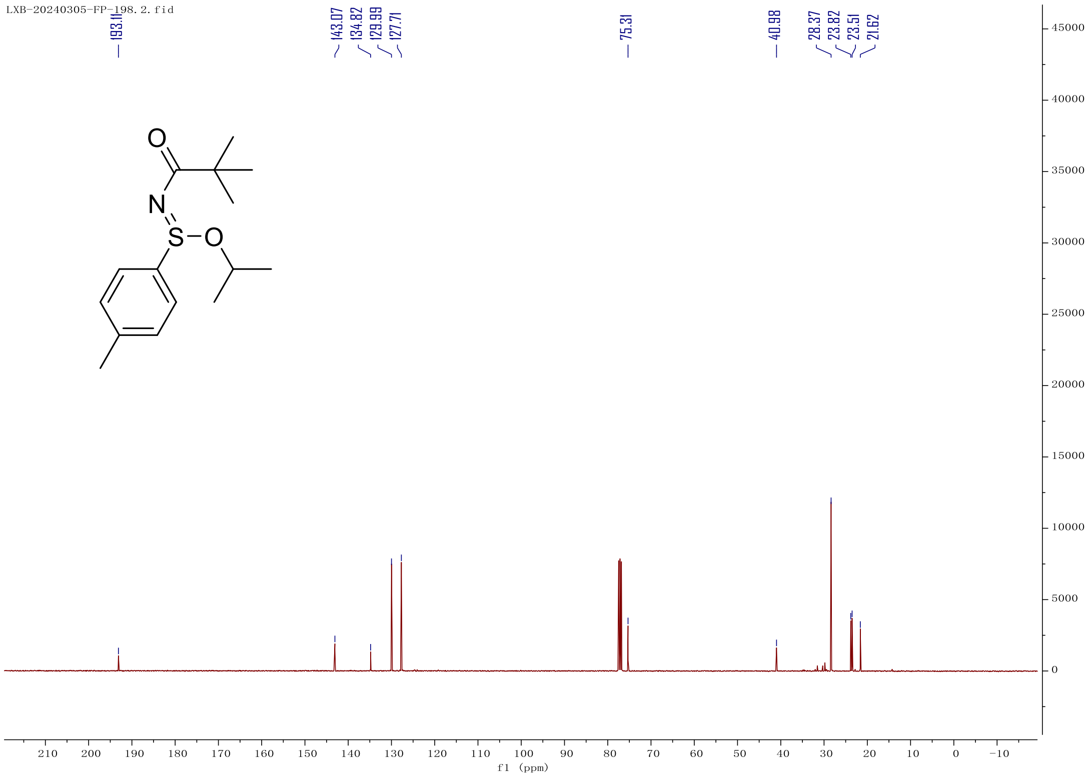

^{1}H NMR spectrum for compound **3t’** (in $CDCl_3$, 400 MHz)

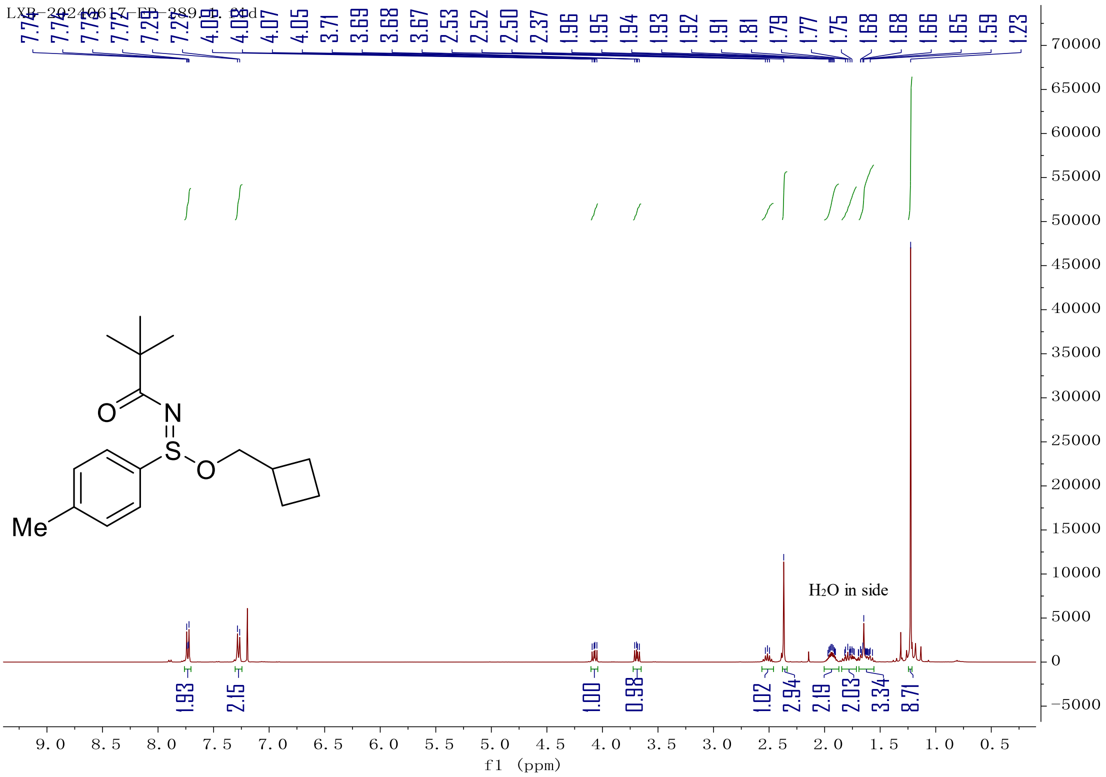

^{1}H NMR spectrum for compound **3u’** (in $CDCl_3$, 400 MHz)

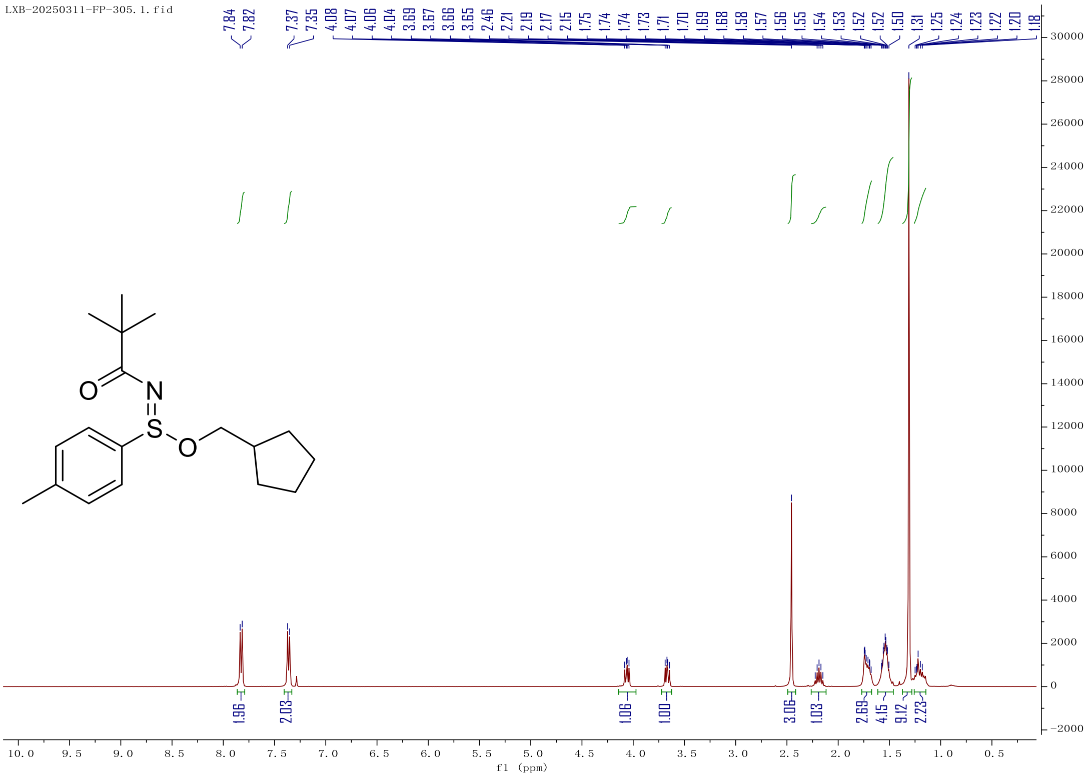

^{1}H NMR spectrum for compound **3v’** (in CDCl3, 400 MHz)

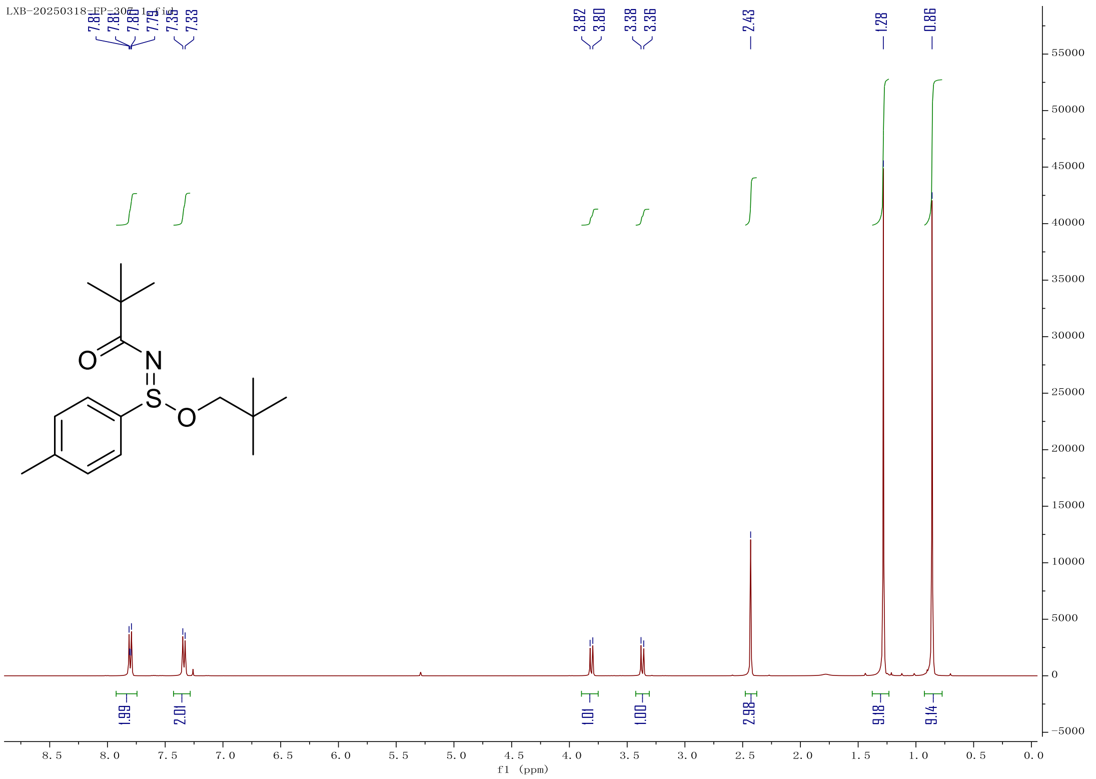

^{1}H NMR spectrum for compound **3w’** (in CDCl3, 400 MHz)

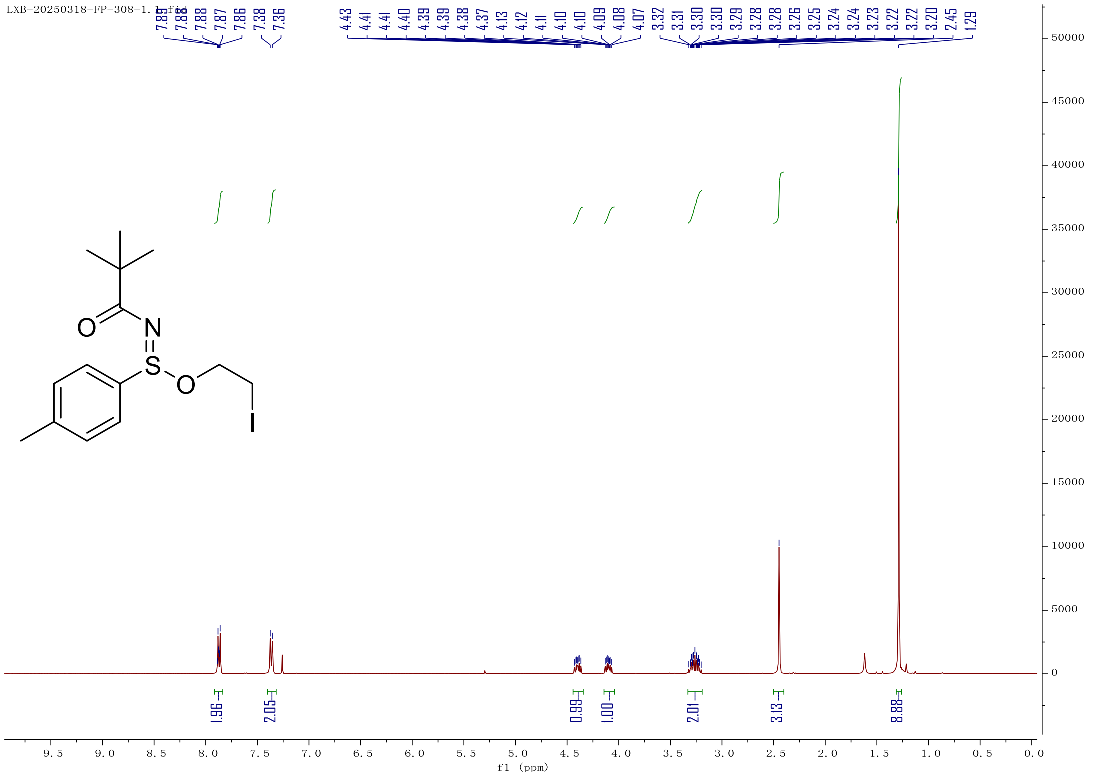

^{1}H NMR spectrum for compound **3x'** (in CDCl3, 400 MHz)

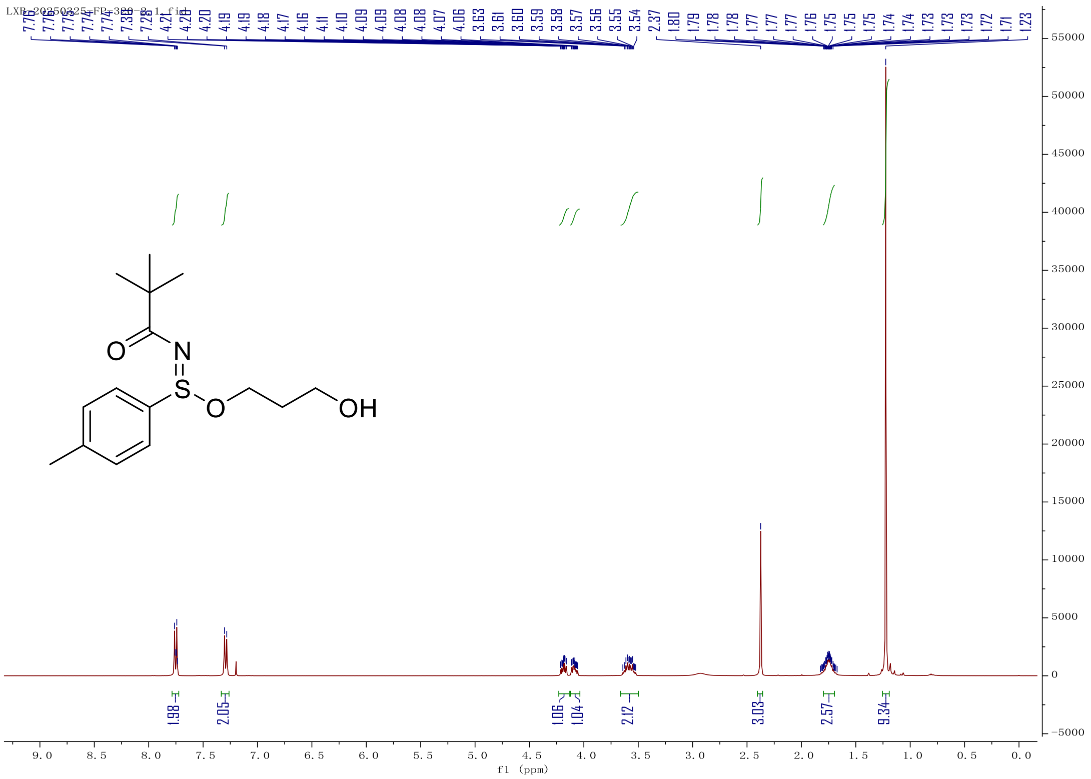

^{13}C NMR spectrum for compound **3x'** (in CDCl3, 101 MHz)

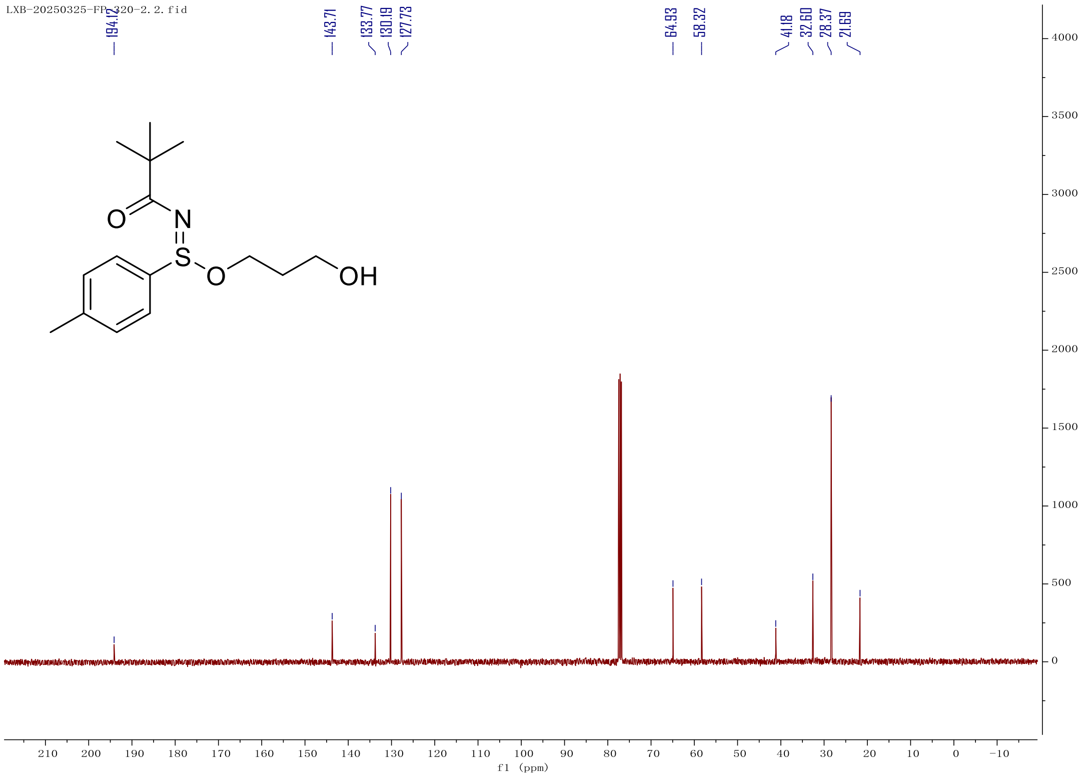

^{1}H NMR spectrum for compound **4** (in CDCl3, 400 MHz)

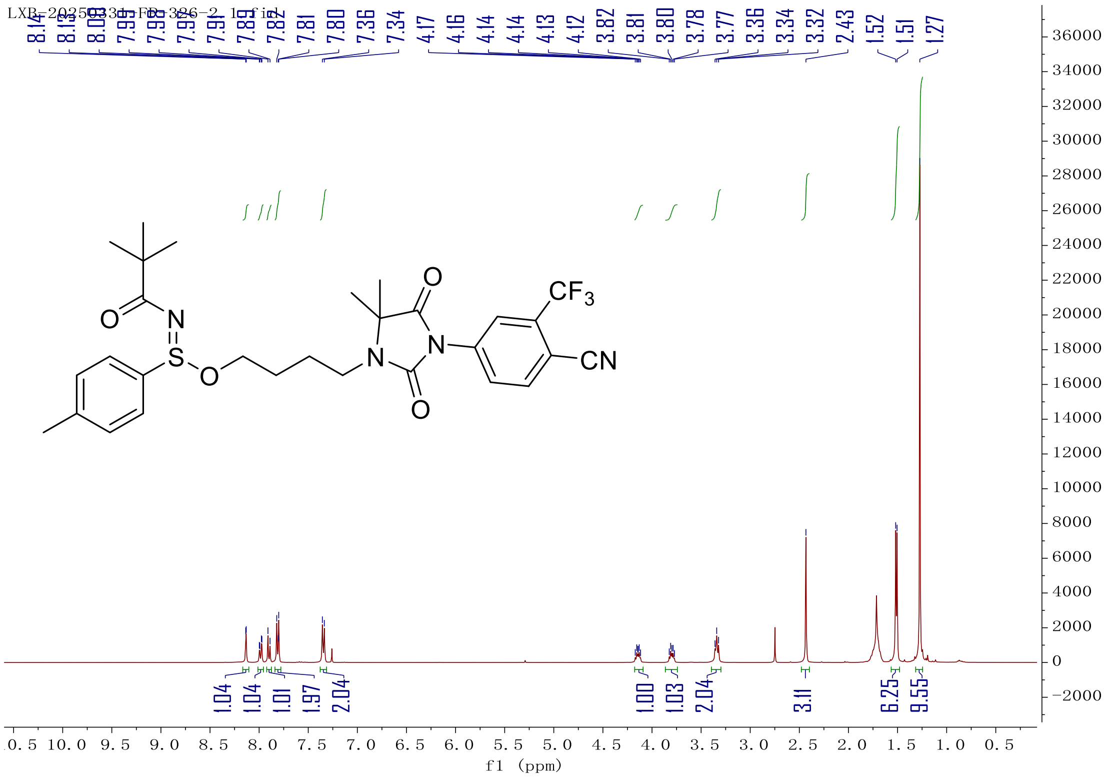

^{19}F NMR spectrum for compound **4** (in CDCl3, 376 MHz)

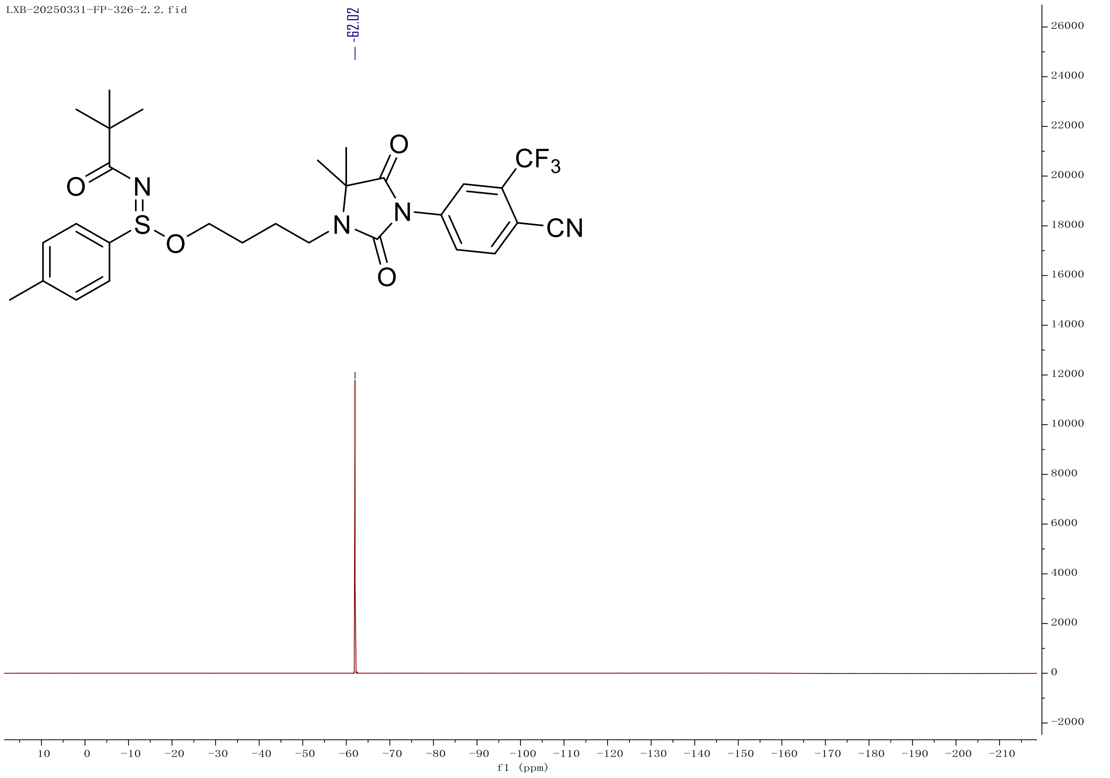

^{1}H NMR spectrum of the major diastereomer of compound **5** (in $CDCl_3$, 400 MHz).

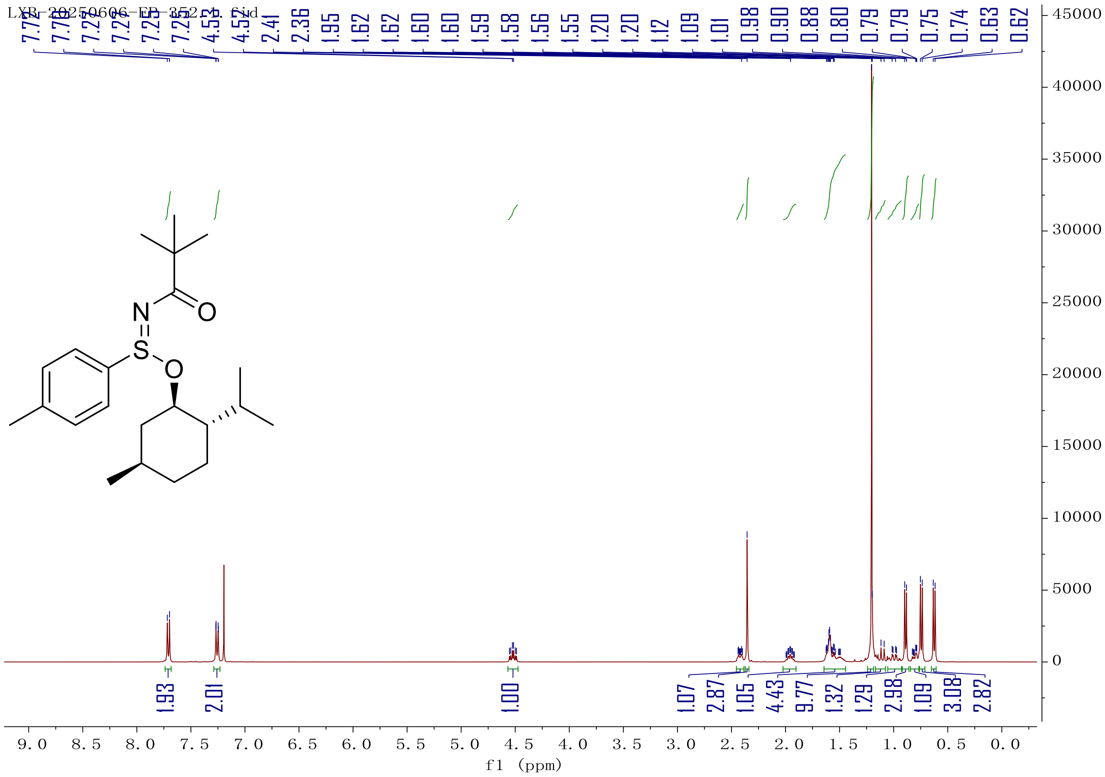

^{13}C NMR spectrum of the major diastereomer of compound **5** (in $CDCl_3$, 101 MHz)

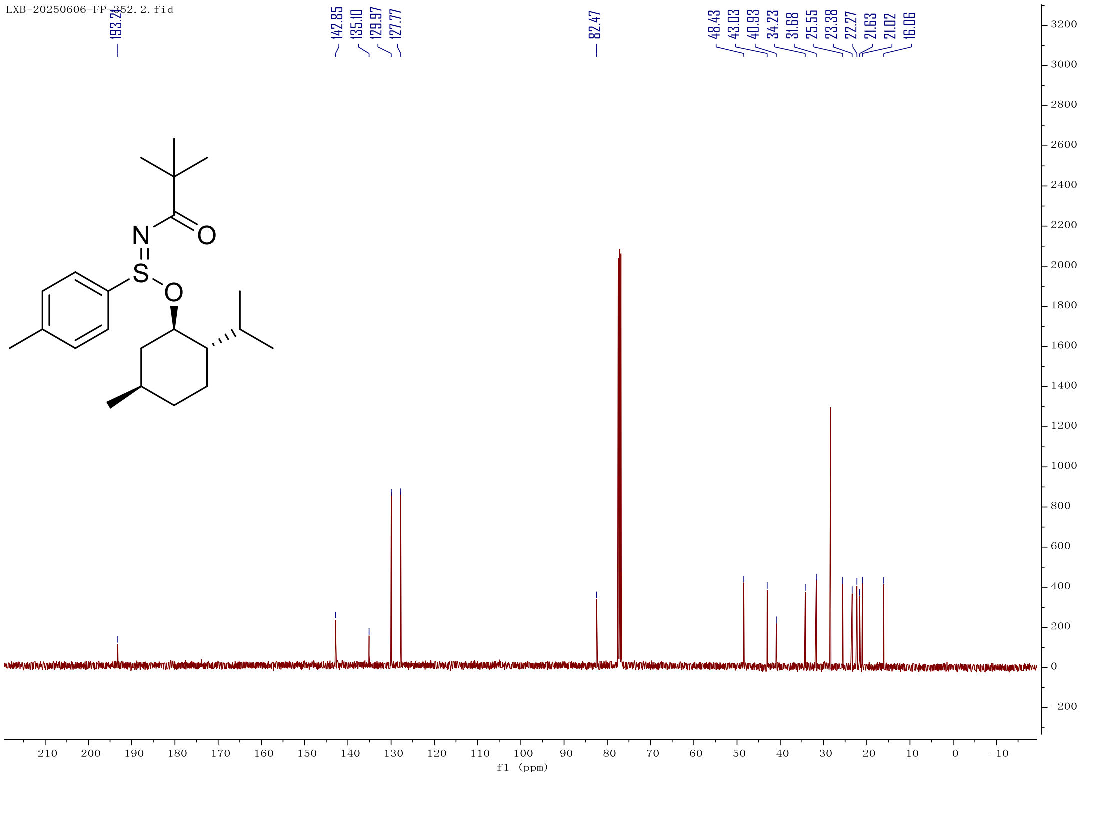

^{1}H NMR spectrum of the minor diastereomer of compound **5** (in $CDCl_3$, 400 MHz).

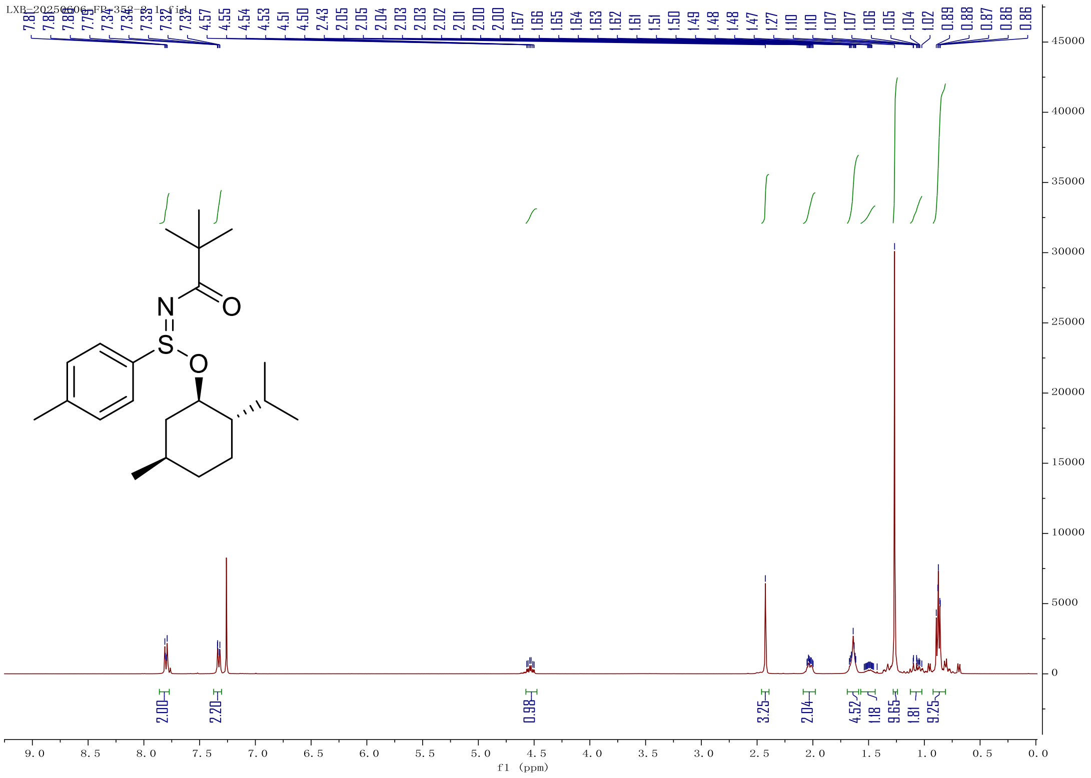

^{13}C NMR spectrum of the minor diastereomer of compound **5** (in $CDCl_3$, 101 MHz)

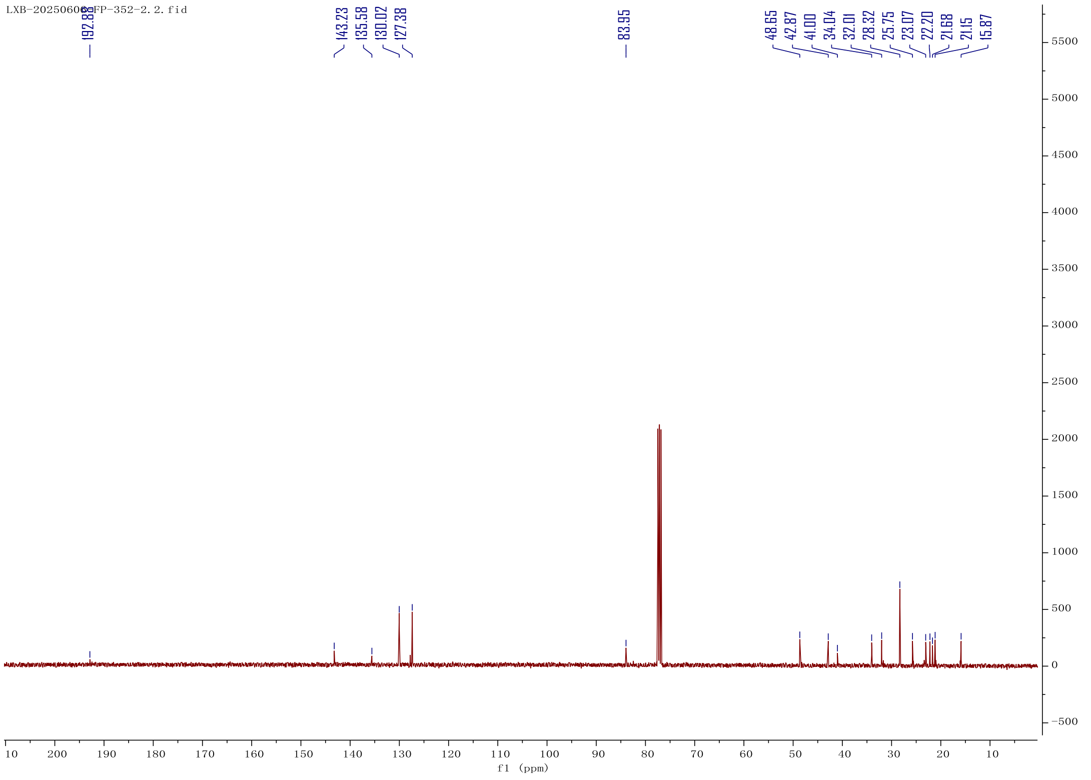

^{1}H NMR spectrum for compound **6a** (in $CDCl_3$, 400 MHz)

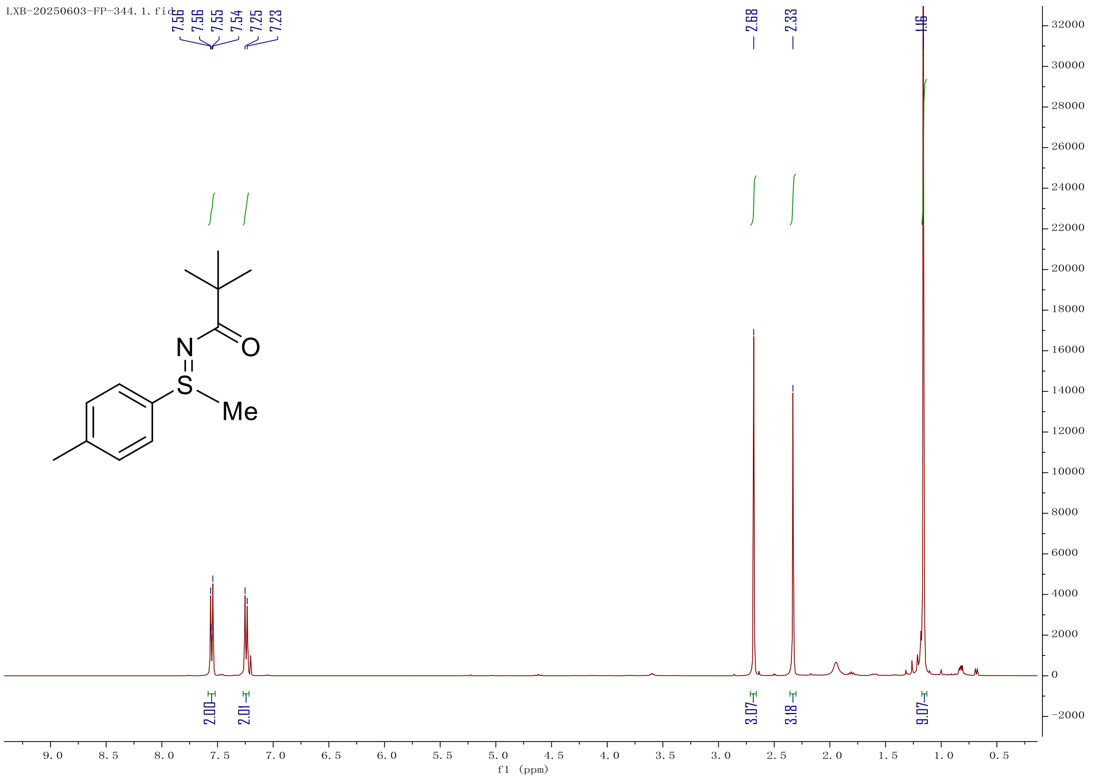

^{1}H NMR spectrum for compound **6b** (in $CDCl_3$, 400 MHz)

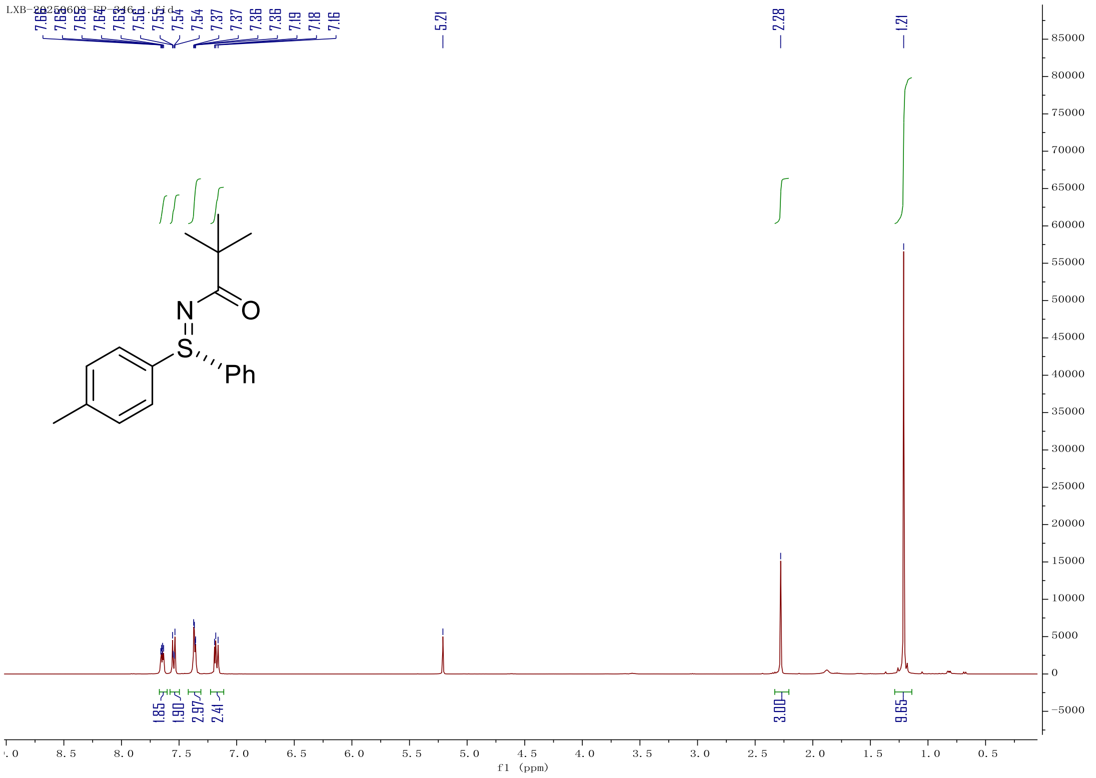

Supplement: File 1 — Experimental procedures, characterization data and copies of spectra. [file Beilstein_J_Org_Chem-22-158-s001.pdf]
